# Supplementary material for: A systematic review of the validity of patient derived xenograft (PDX) models: the implications for translational research and personalised medicine
Source: PeerJ. 2018 Nov 21;6:e5981. doi: 10.7717/peerj.5981 (PMC6252062; doi:10.7717/peerj.5981)
Supplement: Table S2 [file peerj-06-5981-s002.pdf]

Supplemental Table S2: **Search strategies and inclusion/exclusion criteria**

conducted on 12/7/17

Embase <1974 to 2017 July 11>

1 xenograft/ (40270)
2 (xenograft$ or explant$ or explants).ti,ab. (123979)
3 1 or 2 (133527)
4 ((patient$ or human$) adj2 derived).ti,ab. (44918)
5 3 and 4 (5520)
6 conference$.pt. (3369534)
7 5 not 6 (2608)

MEDLINE(R) <1946 to June Week 5 2017>

1 Heterografts/ (3905)
2 (xenograft$ or explant$ or explants).ti,ab. (79098)
3 1 or 2 (81226)
4 ((patient$ or human$) adj2 derived).ti,ab. (28090)
5 3 and 4 (1964)

MEDLINE(R) Epub Ahead of Print <July 11, 2017>,
MEDLINE(R) In-Process & Other Non-Indexed Citations <July 11, 2017>,
MEDLINE(R) Daily Update <July 10, 2017>

1 Heterografts/ (12)
2 (xenograft$ or explant$ or explants).ti,ab. (10387)
3 1 or 2 (10392)
4 ((patient$ or human$) adj2 derived).ti,ab. (4102)
5 3 and 4 (742)

Inclusion / Exclusion criteria

	**Include**	**Exclude**
**Population**	Murine xenografts derived from human carcinoma tissues of breast, colon, lung or prostate (fragments or freshly isolated primary cells at ≤3 passages)	Metastatic tumours<br><br>Lymphoma, leukaemia, sarcomas, melanoma, brain tumours (glioma, medullablastoma etc.), squamous cell carcinoma, cystic carcinoma, teratoma<br><br>Xenografts derived from cell lines or primary samples ≥4 passages (or unclear passage) or genetically manipulated cells<br><br>Rat, canine xenografts
**Outcomes**	Validation:<br><br>1. Xenograft and tumour histology<br>2. Confirmation of human tumour cells (exclusion of mouse cells and normal human cells for primary outgrowth only)<br>3. Confirmation of tissue type<br>4. Confirmation of cellular type<br>5. Confirmation of tumour markers<br>6. Confirmation of lymphoma or EBV (T cell, B cell, NK cell)	
**Outcomes**<br><br>**For extraction but not inclusion**	7. Engraftment rate (primary outgrowth); percentage of biopsies which develop xenografts<br>8. Latency<br>9. Percentage of biopsies resulting in stable lines<br>10. Percentage of biopsies resulting in lymphomas<br>11. Clinical characteristics of patient tumour tissue	
