# Supplementary material for: A systematic review of the validity of patient derived xenograft (PDX) models: the implications for translational research and personalised medicine
Source: PeerJ. 2018 Nov 21;6:e5981. doi: 10.7717/peerj.5981 (PMC6252062; doi:10.7717/peerj.5981)
Supplement: Table S3 [file peerj-06-5981-s003.pdf]

Supplemental Table S3. Model Validity Tool

	Signalling question	Project specific notes	Decision	Justification
REPORTING	**1. Ethical statement** (Was an ethical statement provided for animal/ human tissue handling?)	Details should be provided for mice and patients. Select partial if only one is reported.	Yes/ no/ partial/ NR	Free text to justify decision
	**2. Clear description of model details** (source, species, strain sex, developmental stage, age, passage number etc)	Provide details for mouse; age, strain and source for 'yes', anything else is partial	Yes/ no/ partial/ NR	Free text to justify decision
	**3. Is the model transgenic?** (Whether purchased or created)	Excluded from the review	NA	
	**4. Clear description of the routine maintenance of the model**	yes/no	Yes/ no/ partial/ NR	Free text to justify decision
	**5. Further preparation of model for experiment**	Include here additional factors inoculated with the tissue e.g. FB or Matrigel. Were the PDX tumours passaged?		Free text only
VALIDATION	**6. Stem cell authentication.** (Evidence that cells can divide and renew for long periods; are undifferentiated; multipotent.)	Not identified	NA	
	**7. Cell line authentication:** (source clearly stated, cell line authentication methods, routine checks for the absence of mycoplasma or other contaminants?)	Excluded from the review	NA	
	**8. Primary cultures/ xenografts authentication**	If primary cultures are not used state NA here and do not answer a-h. Risk of bias: High/ low /Unclear/NA. High = there was a concern for one or more PDX. Unclear = not all PDX analysed, or unclear methods		
	**a: was the tissue of origin tracked/ proven?**	Tissue specific markers required. Briefly list methods and mutations. Were all samples analysed?	High/ low /Unclear/NA	Free text to justify decision
	**b. confirmation that the culture or xenograft was derived from a given patient**	Genotyping. Briefly list methods and mutations. Were all samples analysed?	High/ low /Unclear/NA	Free text to justify decision

	**c. was the cell type of interest proven?**	e.g. epithelial or neuroendocrine. Briefly list methods and mutations. Were all samples analysed?	High/ low /Unclear/NA	Free text to justify decision
	**d. confirmation of tumour or normal cells**	Tumour markers or demonstration of serial transplantation. Either is required for yes. Exclusion of normal cells for primary outgrowth only. Briefly list methods and mutations. Were all samples analysed?	High/ low /Unclear/NA	Free text to justify decision
	**e. xenograft only: was the absence of mouse (host) cells proven?**	Mouse specific markers or genotyping (STR), mutations in agreement with patient. Briefly list methods and markers. Were all samples analysed?	High/ low /Unclear/NA	Free text to justify decision
	**f. xenograft only: was the xenograft comparable to the parent tumour by histology?**	Were the results confirmed by an independent pathologist. Were details of the quantitation provided (slides areas of slides). Briefly list methods. Were all samples analysed?	High/ low /Unclear/NA	Free text to justify decision
	**g. xenograft only: was there concordance between the PDX and the patient for response to standard of care/ treatment**	Briefly list methods. Were all samples analysed? correlations or R values	High/ low /Unclear/NA	Free text to justify decision
	**h. xenograft only: were EBV markers evaluated or the presence of lymphomas?**	Including B Cell, T cell, NK cell markers. Briefly list methods and markers. Were all samples analysed?	High/ low /Unclear/NA	Free text to justify decision
	**9. Additional comments/ concerns**		High/ low /Unclear/NA	Free text to justify decision
	**Overall rating/reporting of model**		**Low= all domains clearly reported, and there are no concerns with model. Unclear = Any domains are unclear, but not high risk. High risk = there is a concern of high risk**	**Text to justify why model was given unclear or high rating**

Assessments for Prostate PDX (question 8)

ID	a: was the tissue of origin tracked/ proven?		b. confirmation that the culture or xenograft was derived from a given patient		c. was the cell type of interest proven?		d. confirmation of tumour or normal cells	
Aparicio 2016	High	PSA- for 3/5; AR+ 3/5. Note 2/5 no evidence for prostate tissue	U/NR	No details	High	PSA- for 3/5; AR+ 3/5. Note 2/5 no evidence for epithelial origin	U/NR	It is alluded to in the manuscript that the PDX were serially transplantable lines, but the number of generations was not reported
Tzelepi 2012	High	PSA- for 8/17 (IHC), AR+ 10/17. Note 11/17 no evidence for prostate tissue	U/NR	No details	Low	PSA- for 8/17 (IHC); AR+ 10/17. Note 11/17 no evidence for epithelial tissue, but all + for neuroendocrine markers (chromogranin a and synaptophysin)	U/NR	14 of 19 PDX taken forward for validation and it is alluded to in manuscript that they were serially transplantable lines, but the number of generations was not reported
Aparicio 2011	High	PSA- for 4/4 (IHC); AR+ 2/4; 1 sample + TMPRSS2:ERG gene fusion (results not shown, other PDX not reported). Note 144-2 no evidence for prostate tissue	U/NR	No details	High	PSA using IHC was - for 4/4; AR+ 2/4; 144-4 positive for CK. Note 144-2 no evidence for epithelial origin	U/NR	It is alluded to in the manuscript that PDX were serially transplantable lines, but the number of generations was not reported. 144-4 has a TMPRSS2:ERG gene fusion (results not shown, other PDX not reported)
Chen 2013	High	All lines negative for PSA, by IHC (4 samples), RT-PCR (9 samples) and Western blotting (10 samples). Two samples shown, author reports all other lines were the same	U/NR	Cytogenic analysis confirmed loss in Ch10, in 2/11 PDX. 1 PDX similar to carrier Hs5 cells. Comparison to patient donor not carried out	High	Weak expression of P63 in 3 of 11 lines by IHC and WB. CK 18 + by WB (6 of 11). Positive controls overexposed and negative control (stromal Hs5 line) positive	Low	Serial transplantation demonstrated
Lawrence 2015	U/NR	No details	U/NR	No details	U/NR	IHC for CK8/18. Quantified tumour cells per graft. Defined cancer cells as CK8/18+/P63-. Evidence for 2 samples reported	U/NR	IHC for CK/18+/P63- (definition for prostate cancer). Primary grafts. No serial transplantation

**Li 2012**	Low	Both PDX secrete PSA (serum levels determined by ELISA)	U/NR	No details	Low	Serum PSA detected by ELISA. PSA and TMPRSS2 levels also determined by RT-PCR. RNA data demonstrated for 1 of 2 PDX	Low	Serial transplantation demonstrated
**Lin 2013**	Low	9 of 9 PDX express PSA (IHC): A representative image of 1 PDX shown. 6 of 8 express TMPRSS2:ERG (RNA seq; Microarray based gene expression.	U/NR	Chromosomal copy number profiles compared to original patient tissue. Only 3 of 9 analysed. CN profiling utilized the Agilent SurePrint G3 Human CGH microarray platforms	Low	9 of 9 PSA positive (IHC). Image of 1 PDX demonstrated. Remaining results tabulated.	Low	Serial transplantation demonstrated for all lines. Copy number changes and mutations for 9 of 9
**Presnell 2001**	U/NR	IHC image of 1 of 5 xenografts showed positivity for PSA	U/NR	No details	U/NR	1 of 5 PDX analysed for PSA/CKs by IHC	U/NR	5 of 13 PDX diagnosed as cancer by a uro-pathologist. One graft had areas of benign glands and squamous metaplasia. The absence of benign cells was confirmed in 1 graft by positivity for PSA and absence of basal cells using antibodies against high molecular weight keratins. All grafts were primary explants

**Risbridger 2015**	U/NR	a-methylacylcoenzymeA racemase (AMACR) was evaluated by IHC. One sample shown, results tabulated but the AMACR results were not clear. 78 of 106 xenografts scored as either intraductal carcinoma or adenocarcinoma. IHC for PSA or NKX3.1 was used in some grafts (Toivanen)	U/NR	4 of 16 patient tissues and corresponding pooled xenografts were screened for copy number alterations on Affymetrix OncoScan platform v.2 and v.3. Analysis included detection of common areas of gain–loss on each chromosome between the original specimen and the PDX	U/NR	Evaluated epithelial markers: P63 and cytokeratins 8/18. Only one sample reported. IHC for PSA, or NKX3.1 or CK8/18. Staining carried out for all grafts and results tabulated (Toivanen)	U/NR	Primary explants. Evaluated as cancer by 2 pathologists for adenocarcinoma or intraductal carcinoma. IDC-P reported using criteria established by Montironi and others, including the presence of basal cells, cribriform architecture, and comedonecrosis together with markers for p63, cytokeratins 8/18, AMACR, and ERG. 28/106 xenografts classified as containing non-malignant foci; Risbridger. AMACR or CK18 with P63 loss used to determine the number of tumour foci per graft. Quantitative results (used IHC); Toivanen
**Russell 2015**	High	1 of 3 PDX positive for PSA and PAP (IHC). 2/3 did not express prostate markers	U/NR	Karyotyped PDX but not patient tissue	High	2 of 3 PDX were + for CK7/8 and epithelial membrane antigen (IHC). No results for one PDX, presumed negative.	Low	serial transplantation demonstrated. 1 PDX positive for CEA
**Wetterauer 2015**	U/NR	Assessed by IHC. No prostate tissue-specific markers used, yet pathologist diagnosed 2 of 10 PDX as prostate. The remaining 7 of 10 PDX were + for human B and T lymphoid markers	U/NR	10 of 10 PDX matched their respective patient tissue using short tandem repeat profiling, stated in text, no details provided.	High	Epithelial origin validated for 2 of 10 PDX	U/NR	All primary explants assessed after 3 months. The remaining grafts were verified as human lymphoma using B and T cell markers
**Yoshikawa 2016**	Low	PDX positive for PSA by IHC. Sera of mice also positive for PSA using chemiluminescent immunoassay	Low	Identical AR substitution mutation H875Y (Sanger sequencing)	Low	Cytokeratin 18 positive by IHC	Low	Stable line reported. PDX also positive for the prostate tumour marker, AMACR

**van Weerden, 1996**	High	3 of 5 PDX express PSA and PAP. Both markers assessed by IHC but images not shown. PSA also analysed by Northern blot for RNA expression. Homogenates and plasma assessed by ELISA. samples were negative	High	DNA ploidy was undertaken but only for PDX. PSA expression was negative for 2 PDX, which was not in agreement with original tissue (positive) for PSA, PAP and AR.	High	PSA and PAP was detected in 3 of 5 lines. No other epithelial markers used. 2 PDX were negative	Low	5 of 5 PDX were capable of serial transplantation
**Pretlow 1993**	U/NR	1 of 4 stable lines express PSA. Did not report how the analysis was carried out for 3 lines. RNA expression and quantitative immunoassay used to measure plasma PSA for 1 line	U/NR	partial Karyotype analysis of primary outgrowths and some stable lines. But did not compare to patient	U/NR	1 of 4 confirmed EGFR expression by RT-PCR. RNA expression for PSA in 1 of 4 PDX	Low	confirmed by serial transplantation for 4 PDX. Others either regressed or were static. Chromosomal aberrations reported for some lines, but only for 2 of 4 stable lines
**Klein 1997**	High	1 of 2 PDX express PSA by RT-PCR using human specific primers	U/NR	Karyotyped PDX but not patient tissue	High	1 of 2 PDX express PSA	Low	both PDX capable of serial transplantation. 1 PDX has a tetraploid karyotype. 1 of 2 analysed
**Priolo 2010**	U/NR	13 PDX samples screened for AMACR and PSA by IHC and serum PSA levels also quantified by ELISA. Authors state all were positive but no results to confirm. Unclear how many tumours and mice were analysed per patient	U/NR	aCGH carried out on 7 of 13 PDX samples and corresponding donor tissue. Identical genetic alterations between pairs	U/NR	13 PDX samples screened for PSA expression by IHC	U/NR	All primary explants. 13 of 23 explants verified as prostate cancer by a pathologist and screened for AMACR. TMPRSS2:ERG also verified in 5 pairs by FISH and genetic alterations (using aCGH) verified in 7 of 13 pairs
**Wang 2005**	U/NR	Early passage line weakly positive for PSA with scant AR (IHC). RNA expression of high passage line suggestive of PSA expression, but protein expression not carried out at high passage	U/NR	SKY Karyotype of PDX was not compared to original patient tissue	U/NR	Weakly positive for PSA. However, at the F8 generation, PSA was very weak. No other epithelial markers were used to determine cell provenance	Low	Serially transplantable line, but karyotype is atypical of a prostate tumour. Diploid with few genetic alterations suggestive of proliferation of normal (lymphoid) cells

**Terada 2010**	Low	Western blotting analysis revealed that KUCaP-2 cells expressed AR and PSA	U/NR	Sequence analysis of AR in KUCaP-2 tumours before and after castration showed no AR mutation	Low	IHC for PSA had poor images but evidence that cell type is epithelial	Low	Stable line reported
**Toivanen 2011**	U/NR	PSA expression was evident in 2 patient and PDX models. AMACR expression presented for 6 patients and PDX	U/NR	No details	U/NR	PSA, CK8/18 expression was evident for 2 patients and PDX models	Low	AMACR expression presented in 6 patients and PDX. Prostate cancer confirmed by the presence of CK8/18 and loss of P63. IHC evidence presented for some xenografts, results were tabulated

**ID**	**e. xenograft only: was the absence of mouse (host) cells proven?**		**f. xenograft only: was the xenograft compared to the parent tumour by histology?**		**g. xenograft only: was there concordance between the PDX and the patient for response to standard of care/ treatment**		**h. xenograft only: were EBV markers evaluated or the presence of lymphomas?**		**Overall rating/reporting of model.**	
**Aparicio 2016**	Low	Human markers utilized (using a combination of IHC, WB RT-PCR, ELISA)	U/NR	Not reported	U/NR	No details	U/NR	No details	**High**	2/5 PDX not proven to be prostate
**Tzelepi 2012**	Low	Human markers utilized (using a combination of IHC, WB RT-PCR, ELISA). 17 PDX validated as human	U/NR	Pathologist scored slides, but only 7 PDX presented	U/NR	No details	U/NR	No details	**High**	11/17 PDX not proven to be prostate
**Aparicio 2011**	Low	Human markers utilized (using a combination of IHC, WB RT-PCR, ELISA). 4 of 4 PDX validated as human	U/NR	Pathologist scored slides, but only PDX 144-4 results presented	U/NR	No details	U/NR	No details	**High**	1/4 not proven to be prostate or epithelial

**Chen 2013**	U/NR	Human specific antibodies for mitochondria and Ki67, evidence for 4/11 PDX	High	4 of 11 compared to patient. Undifferentiated histology was observed in PDX lines which did not compare to the Gleason 7 patient tumours. Pathologist involvement not reported.	U/NR	No details	U/NR	No details	**High**	No evidence that the PDX were prostate derived, CK was positive in negative control (W Blot), therefore concern for epithelial origin. PDX and patient histology were not comparable
**Lawrence 2015**	U/NR	IHC for human keratin, but only 2 samples reported	U/NR	2 PDX compared to patient tissue	High	only 1 of 2 PDX explants responded to castration, yet both patients were hormone responsive at time of biopsy	U/NR	No details	**High**	Tissue origin not proven, only 1 of 2 PDX explants responded to castration from hormone responsive biopsies
**Li 2012**	Low	PSA detected in serum of mouse. TMPRSS2 is human and prostate cancer specific	U/NR	No details	U/NR	No details	U/NR	No details	**U/NR**	not all validation criteria were met
**Lin 2013**	Low	9 of 9 PDX demonstrated chromosomal changes indicative of prostate cancer. IHC for PSA 9 of 9	U/NR	3 of 9 PDX only	High	8 of 9 PDX androgen sensitive. 2 patients unresponsive to ADT	Low	2 PDX terminated due to development of B-cell lymphoma. Results not shown	**High**	high risk due to lack of concordance with ADT
**Presnell 2001**	Low	1 of 5 grafts expressed PSA by IHC. Pathologist diagnosed the correct Gleason Grade for 5 of 5	Low	5 of 5 explants compared with original patient tissue by IHC and assessed by a uro-pathologist	U/NR	No details	U/NR	No details	**U/NR**	not all validation criteria were met

**Risbridger 2015**	U/NR	Copy number changes for 5/16 xenografts and pathological examination confirming cancer in 67/106 xenografts. Used human specific antibodies, but did not control for the use of mouse SVM, which was used to support the grafts (Toivanen)	High	Data not shown, but stated xenografts were compared to patient tissue (Risbridger). Discordance between Gleason pattern of patient specimen and Gleason pattern of engrafted tissues. Only 2 of 12 patient's samples had identical Gleason grade and pattern. Authors state that 'The Gleason patterns of the index tumour reported at patient diagnosis and the tumour region acquired for the study were comparable to those in engrafted tissues' (Toivanen)	U/NR	No details	U/NR	No details	**High**	Discordance with histology of PT and PDX (Toivanen)
**Russell 2015**	U/NR	1 line overgrown by murine cells (Human DNA sequences not detectable by human Alu repeat sequence element probing and lack of staining for human histocompatibility antigens)	U/NR	Histology compared for 1 of 3 PDX. Unclear pathologist involvement	U/NR	One PDX showed testosterone responsiveness but it was unclear whether or not the original tumour was castrate-resistant	U/NR	1 of 3 PDX was a murine lymphoma/fibrosarcoma - looked for lack of expression of human markers and loss of human DNA sequences	**High**	prostate provenance not proven for 2 PDX

**Wetterauer 2015**	Low	Used human specific antibodies and demonstrated the presence of EBV in 7 of 10 grafts. EBV infects only human cells	U/NR	No details	U/NR	No details	Low	7 of 10 PDX EBV+. Used a combination of in situ hybridisation and IHC for EBV associated genes and proteins. 8/10 had lymphoma histology	**U/NR**	not all validation criteria were met
**Yoshikawa 2016**	Low	LC-MS/MS analysis of sera from mice and fluid from the tumours established that a high percentage of human proteins were secreted	U/NR	Unclear whether pathologist involved. PDX histology is papillary, but unclear if the patent tumour was similar as description not reported for patient	Low	PDX established from a CRPC patient. PDX showed a good response to ADT, but quickly relapsed. Patient also resistant to anti-androgens	U/NR	No details	**U/NR**	not all validation criteria were met
**van Weerden 1996**	U/NR	At each transplant generation PDX was examined for its human origin. Abisbenzimide (Hoechst H-33258) staining, which allows the discrimination between mouse (stromal) cells and human (prostate epithelial) cells, was performed. Results not shown	High	3 PDX (diagnosed as adenocarcinoma), 2 lines poorly differentiated, characterized by a small cell phenotype. Squamous differentiation observed in one PDX. Does not correspond with patient. Uropathologist co-author	U/NR	No details	U/NR	No details	**High**	2 PDX were not proven to be prostate or from an epithelial lineage. Both had characteristics which did not match the original tissue
**Pretlow 1993**	U/NR	1 of 4 lines express and secrete PSA. The remaining stable lines were not assessed for mouse content	U/NR	Results not shown. Histology confirmed in table 1 but few methodological details given	U/NR	No details	U/NR	No details	**U/NR**	not all validation criteria were met

**Klein 1997**	Low	Human beta globin primers used to determine human DNA content showed both PDX were human	U/NR	No details	U/NR	No details	Low	CD45 lymphoid marker used. 1 of 2 PDX verified as human lymphoma	**U/NR**	not all validation criteria were met. Not high risk as 1 PDX was verified as lymphoma
**Priolo 2010**	Low	Human specific antibodies used in IHC. PSA also human specific	U/NR	No details	U/NR	No details	U/NR	No details	**U/NR**	not all validation criteria were met
**Wang 2005**	Low	Human antibodies and mouse and human specific primers used suggest a human origin	High	Morphology of patient and PDX at low passage are similar, but small cells with little stroma was obvious at high passage	U/NR	No details	U/NR	No details	**High**	Weak staining, atypical morphology and karyotype suggest that this line is not prostate
**Terada 2010**	U/NR	Reported human PSA expression and secretion - unlikely to be murine	U/NR	Unclear whether pathologist involved. Unclear if histology of PDX matched patient's tissue.	U/NR	Unclear what the patient response to androgens was. The sample used to generate the PDX was from a radical prostatectomy, so unlikely that they would have had hormone therapy at the time of biopsy	U/NR	No details	**U/NR**	not all validation criteria were met
**Toivanen 2011**	U/NR	1 PDX reported. Used FISH of human telomeres and centromeres	U/NR	No images of original patient histology. No PDX histology was tabulated with the original tissue	U/NR	No details	U/NR	No details	**U/NR**	not all validation criteria were met
