# Supplementary material for: A systematic review of the validity of patient derived xenograft (PDX) models: the implications for translational research and personalised medicine
Source: PeerJ. 2018 Nov 21;6:e5981. doi: 10.7717/peerj.5981 (PMC6252062; doi:10.7717/peerj.5981)
Supplement: Table S4 [file peerj-06-5981-s004.pdf]

Supplemental Table S4: Excluded studies and reasons for exclusion

	Reference
Duplicate	Jin, K., et al. Antitumor effects of FP3 in combination with capecitabine on PDTT xenograft models of primary colon carcinoma and related lymphatic and hepatic metastases 2012 Cancer Biology & Therapy 13(9) 737-44
Exclude [≥3 passages]	Drews-Elger, K., et al. Primary breast tumor-derived cellular models: Characterization of tumorigenic, metastatic, and cancer-associated fibroblasts in dissociated tumor (DT) cultures 2014 Breast Cancer Research and Treatment 144(3) 503-517
	Gebauer, F., et al. Establishment and characterization of a pair of patient-derived human non-small cell lung cancer cell lines from a primary tumor and corresponding lymph node metastasis 2016 Anticancer Research 36(4) 1507-1518
	Timofeeva, O. A., et al. Conditionally reprogrammed normal and primary tumor prostate epithelial cells: A novel patient-derived cell model for studies of human prostate cancer 2017 Oncotarget 8(14) 22741-22758
	Amendt, C., et al. 2014 Clinical cancer research: an official journal of the American Association for Cancer Research 20(17) 4478-87
	Mather, J., et al. Isolation of cancer stem like cells from human adenosquamous carcinoma of the lung supports a monoclonal origin from a multipotential tissue stem cell. 2013 PloS one 8(12) e79456
	Silvers,C ., et al. A novel in vitro assay of tumor-initiating cells in xenograft prostate tumors. 2010 The Prostate 70(13) 1379-87
Exclude [cell line]	Adumeau, P., et al. A pretargeted approach for the multimodal PET/NIRF imaging of colorectal cancer 2016 Theranostics 6(12) 2267-2277
	Amir, S., et al. MSF-A interacts with hypoxia-lnducible factor-1alphaand augments hypoxia-inducible factor transcriptional activation to affect tumorigenicity and angiogenesis 2006 Cancer Research 66(2) 856-866
	Anderson, G. R., et al. PIK3CA mutations enable targeting of a breast tumor dependency through mTOR-mediated MCL-1 translation 2016 Science Translational Medicine 8: 369ra175
	Balko, J. M., et al. Triple-negative breast cancers with amplification of JAK2 at the 9p24 locus demonstrate JAK2-specific dependence 2016 Science Translational Medicine 8(334) doi:10.1126/scitranslmed.aad3001
	Bansal, N., et al. BMI-1 targeting interferes with patient-derived tumor-initiating cell survival and tumor growth in prostate cancer 2016 Clinical Cancer Research 22(24) 6176-6191
	Boisgerault, N., et al. Natural oncolytic activity of live-attenuated measles virus against human lung and colorectal adenocarcinomas 2013 BioMed Research International https://doi.org/10.1155/2013/387362.
	Calon, A., et al. Stromal gene expression defines poor-prognosis subtypes in colorectal cancer 2015 Nature Genetics 47(4) 320-329
	Cardo-Vila, M., et al. Interleukin-11 Receptor Is a Candidate Target for Ligand-Directed Therapy in Lung Cancer: Analysis of Clinical Samples and BMTP-11 Preclinical Activity 2016 American Journal of Pathology 186(8) 2162-2170
	Chatalic, K. L. S., et al. A novel ¹¹¹In-labeled anti-prostate-specific membrane antigen nanobody for targeted SPECT/CT imaging of prostate cancer 2015 Journal of Nuclear Medicine 56(7) 1094-1099
	Fox, E. M., et al. Autocrine IGF-I/insulin receptor axis compensates for inhibition of AKT in ER-positive breast cancer cells with resistance to estrogen deprivation 2013 Breast Cancer Research 15(4) R55
	Giedt, R. J., et al. Computational imaging reveals mitochondrial morphology as a biomarker of cancer phenotype and drug response 2016 Scientific Reports 6: 32985
	Gilani, R. A., et al. UM-164: A potent c-Src/p38 kinase inhibitor with in vivo activity against triple-negative breast cancer 2016 Clinical Cancer Research 22(20) 5087-5096

	Hong, S. N., et al. Epigenetic silencing of NDRG2 promotes colorectal cancer proliferation and invasion 2016 Journal of Gastroenterology and Hepatology (Australia) 31(1) 164-171
	Hultberg, A., et al. Depleting MET-expressing tumor cells by ADCC provides a therapeutic advantage over inhibiting HGF/MET signaling 2015 Cancer Research 75(16) 3373-3383
	Hydbring, P., et al. Cell-Cycle-Targeting MicroRNAs as Therapeutic Tools against Refractory Cancers 2017 Cancer Cell 31(4) 576-590.e8
	Jhan, J. R. and Andrechek, E. R. Effective personalized therapy for breast cancer based on predictions of cell signaling pathway activation from gene expression analysis 2017 Oncogene 36(25) 3553-3561
	Khurana, A., et al. Matrix detachment and proteasomal inhibitors diminish Sulf-2 expression in breast cancer cell lines and mouse xenografts 2013 Clinical and Experimental Metastasis 30(4) 407-415
	Kienast, Y., et al. Ang-2-VEGF-A crossmab, a novel bispecific human IgG1 antibody Blocking VEGF-A and Ang-2 functions simultaneously, mediates potent antitumor, antiangiogenic, and antimetastatic efficacy 2013 Clinical Cancer Research 19(24) 6730-6740
	Liu, H., et al. c-Myc alteration determines the therapeutic response to FGFR inhibitors 2017 Clinical Cancer Research 23(4) 974-984
	Lu, P. H., et al. Aqueous Oldenlandia diffusa extracts inhibits colorectal cancer cells via activating AMP-activated protein kinase signalings 2016 Oncotarget 7(29) 45889-45900
	Ma, R., et al. Estrogen Receptor B as a therapeutic target in breast cancer stem cells 2017 Journal of the National Cancer Institute 109(3) djw236. doi: 10.1093/jnci/djw236
	Marques, R. B., et al. High efficacy of combination therapy using PI3K/AKT inhibitors with androgen deprivation in prostate cancer preclinical models 2015 European Urology 67(6) 1177-1185
	Mittal, K., et al. Amplified centrosomes and mitotic index display poor concordance between patient tumors and cultured cancer cells 2017 Scientific Reports 7: 43984. doi:10.1038/srep43984.
	Nakahara, T., et al. YM155, a novel survivin suppressant, enhances taxane-induced apoptosis and tumor regression in a human Calu 6 lung cancer xenograft model 2011 Anti-Cancer Drugs 22(5) 454-462
	Navab, R., et al. Integrin alpha11beta1 regulates cancer stromal stiffness and promotes tumorigenicity and metastasis in non-small cell lung cancer 2016 Oncogene 35(15) 1899-1908
	Nylandsted, J., et al. Eradication of glioblastoma, and breast and colon carcinoma xenografts by Hsp70 depletion 2002 Cancer Research 62(24) 7139-7142
	Ogitani, Y., et al. Bystander killing effect of DS-8201a, a novel anti-human epidermal growth factor receptor 2 antibody-drug conjugate, in tumors with human epidermal growth factor receptor 2 heterogeneity 2016 Cancer Science 107: 1039–1046
	Oikonomou, E., et al. Newly established tumourigenic primary human colon cancer cell lines are sensitive to TRAIL-induced apoptosis in vitro and in vivo 2007 British Journal of Cancer 97(1) 73-84
	Pavet, V., et al. Multivalent DR5 Peptides Activate the TRAIL Death Pathway and Exert Tumoricidal Activity 2010 Cancer Research 70(3) 1101-1110
	Puvirajesinghe, T. M., et al. Identification of p62/SQSTM1 as a component of non-canonical Wnt VANGL2-JNK signalling in breast cancer 2016 Nature Communications 7(10318) https://dx.doi.org/10.1038%2Fncomms10318
	Romanelli, A., et al. Inhibiting aurora kinases reduces tumor growth and suppresses tumor recurrence after chemotherapy in patient-derived triple-negative breast cancer xenografts 2012 Molecular Cancer Therapeutics 11(12) 2693-2703
	Vallespi, M. G., et al. Antitumor efficacy, pharmacokinetic and biodistribution studies of the anticancer peptide CIGB-552 in mouse models 2014 Journal of Peptide Science 20: 10.1002/psc.2676.
	Wakeling, A. E., et al. ZD1839 (Iressa): An orally active inhibitor of epidermal growth factor signaling with potential for cancer therapy 2002 Cancer Research 62(20) 5749-5754
	Wang, F., et al. Selective cytotoxicity to HER2-positive tumor cells by a recombinant e23sFv-TD-tBID protein containing a furin cleavage sequence 2010 Clinical Cancer Research 16(8) 2284-2294

	Wang, J., et al. ABBV-399, a c-Met antibody-drug conjugate that targets both MET-amplified and c-Met-overexpressing tumors, irrespective of MET pathway dependence 2017 Clinical Cancer Research 23(4) 992-1000
	Wang, L., et al. Autophagy inhibition sensitizes WYE-354-induced anti-colon cancer activity in vitro and in vivo 2016 Tumor Biology 37(9) 11743-11752
	Weir, H. M., et al. AZD9496: An oral estrogen receptor inhibitor that blocks the growth of ER-positive and ESR1-mutant breast tumors in preclinical models 2016 Cancer Research 76(11) 3307-3318
	Zhao, Y., et al. Activation of P-TEFb by Androgen Receptor-Regulated Enhancer RNAs in Castration-Resistant Prostate Cancer 2016 Cell Reports 15(3) 599-610
**Exclude [conference abstract]**	Fiebig, H.H., et al. Do gene signatures predict effectiveness of bevacizumab and cetuximab? 2008 Journal of Clinical Oncology 26(15) 14519
**Exclude [manipulation]**	Francescangeli, F., et al. Dynamic regulation of the cancer stem cell compartment by Cripto-1 in colorectal cancer 2015 Cell Death and Differentiation 22(10) 1700-1713
	Green, J. L., et al. Paracrine Wnt signaling both promotes and inhibits human breast tumor growth 2013 Proceedings of the National Academy of Sciences of the United States of America 110(17) 6991-6996
	Hanna, C., et al. Labeling of Breast Cancer Patient-derived Xenografts with Traceable Reporters for Tumor Growth and Metastasis Studies 2016 Journal of Visualized Experiments 117: 10.3791/54944. doi:10.3791/54944.
	Picco, G., et al. A diphtheria toxin resistance marker for in vitro and in vivo selection of stably transduced human cells 2015 Scientific reports 5: 14721
	Powell, E., et al. p53 deficiency linked to B cell translocation gene 2 (BTG2) loss enhances metastatic potential by promoting tumor growth in primary and metastatic sites in patient-derived xenograft (PDX) models of triple-negative breast cancer 2016 Breast Cancer Research 18: 13. doi:10.1186/s13058-016-0673-9.
	Sflomos, G., et al. A Preclinical Model for ERalpha-Positive Breast Cancer Points to the Epithelial Microenvironment as Determinant of Luminal Phenotype and Hormone Response 2016 Cancer Cell 29(3) 407-422
	Shangguan, W., et al. Endothelium originated from colorectal cancer stem cells constitute cancer blood vessels 2017 Cancer Science 108: 1357–1367
	Thiagarajan, P. S., et al. Development of a fluorescent reporter system to delineate cancer stem cells in triple-negative breast cancer 2015 Stem Cells 33(7) 2114-2125
**Exclude [Non-English]**	Hu, Y. R., et al. Establishment of patient-derived xenotransplantation models for non-small cell lung cancer in immune deficient mice 2009 Chinese Journal of Cancer 28: 890-3
	Karpel-Massler, G., et al. Inhibition of Mitochondrial Matrix Chaperones and Antiapoptotic Bcl-2 Family Proteins Empower Antitumor Therapeutic Responses 2017 Cancer Research 77(13) 3513-3526
**Exclude [not mouse model]**	Dayanc, B. E., et al. Enhanced sensitivity of colon tumour cells to natural killer cell cytotoxicity after mild thermal stress is regulated through HSF1-mediated expression of MICA 2013 International Journal of Hyperthermia 29(5) 480-490
	Gelfo, V., et al. A module of inflammatory cytokines defines resistance of colorectal cancer to EGFR inhibitors 2016 Oncotarget 7(44) 72167-72183
	Jaworski, F. M., et al. In Vivo Hemin Conditioning Targets the Vascular and Immunologic Compartments and Restrains Prostate Tumor Development 2017 Clinical Cancer Research: DOI: 10.1158/1078-0432.CCR-17-0112
	Jensen, M. R., et al. NVP-AUY922: a small molecule HSP90 inhibitor with potent antitumor activity in preclinical breast cancer models 2008 Breast cancer research : BCR 10(2) R33
	Jhuraney, A., et al. PAXIP1 Potentiates the Combination of WEE1 Inhibitor AZD1775 and Platinum Agents in Lung Cancer 2016 Molecular Cancer Therapeutics 15(7) 1669-81
	Mascini, N. E., et al. The use of mass spectrometry imaging to predict treatment response of patient-derived xenograft models of triple-negative breast cancer 2015 Journal of Proteome Research 14(2) 1069-1075

	Roman-Roman, P., et al. Modeling tumor growth in the presence of a therapy with an effect on rate growth and variability by means of a modified Gompertz diffusion process 2016 Journal of Theoretical Biology 407: 1-17
	Shen, A., et al. C-Myc alterations confer therapeutic response and acquired resistance to c-Met inhibitors in MET-addicted cancers 2015 Cancer Research 75(21) 4548-4559
	Loning, T H and Mackenzie, I. C. Immunohistochemical studies of basal cell carcinomas transplanted into nude mice. 1986 Archives of dermatological research 279(1) 37-43
Exclude [PDX methods only]	Bagby, S., et al. Development and Maintenance of a Preclinical Patient Derived Tumor Xenograft Model for the Investigation of Novel Anti-Cancer Therapies 2016 Journal of Visualized Experiments 115: doi: 10.3791/54393
	DeRose, Y. S., et al. Patient-derived models of human breast cancer: Protocols for in vitro and in vivo applications in tumor biology and translational medicine 2013 Current Protocols in Pharmacology: 0 14: Unit14.23. doi:10.1002/0471141755.ph1423s60.
	Park, D., et al. Establishment of Patient-derived Xenografts in Mice 2016 Bio protocol 6(22) pii: e2008. doi: 10.21769/BioProtoc.2008.
	Williams, E. S., et al. Generation of Prostate Cancer Patient Derived Xenograft Models from Circulating Tumor Cells 2015 Journal of visualized experiments: JoVE 104: 10.3791/53182.
	Zhang, X. and Lewis, M. T. Establishment of Patient-Derived Xenograft (PDX) Models of Human Breast Cancer 2013 Current Protocols in Mouse Biology 3(1) 21-29
Exclude [PDX not derived in paper]	Isella, C., et al. Stromal contribution to the colorectal cancer transcriptome 2015 Nature Genetics 47(4) 312-319
	Li, H., et al. Integrated analysis of transcriptome in cancer patient-derived xenografts 2015 PLoS ONE 10(5) e0124780. https://doi.org/10.1371/journal.pone.0124780
	Maity, S. N., et al. Targeting of CYP17A1 Lyase by VT-464 Inhibits Adrenal and Intratumoral Androgen Biosynthesis and Tumor Growth of Castration Resistant Prostate Cancer 2016 Scientific Reports 6: 35354
Exclude [purchased or validated elsewhere]	Akamatsu, S., et al. The placental gene PEG10 promotes progression of neuroendocrine prostate cancer 2015 Cell Reports 12(6) 922-936
	Amendt, C., et al. Association of EGFR expression level and cetuximab activity in patient-derived xenograft models of human non-small cell lung cancer 2014 Clinical cancer research: an official journal of the American Association for Cancer Research 20(17) 4478-4487
	Arcaroli, J. J., et al. Common PIK3CA mutants and a novel 3' UTR mutation are associated with increased sensitivity to saracatinib 2012 Clinical Cancer Research 18(9) 2704-2714
	Arcaroli, J. J., et al. A NOTCH1 gene copy number gain is a prognostic indicator of worse survival and a predictive biomarker to a Notch1 targeting antibody in colorectal cancer 2016 International Journal of Cancer 138(1) 195-205
	Barbie, T. U., et al. Targeting an IKBKE cytokine network impairs triple-negative breast cancer growth 2014 Journal of Clinical Investigation 124(12) 5411-5423
	Belmont, P. J., et al. Resistance to dual blockade of the kinases PI3K and mTOR in KRAS-mutant colorectal cancer models results in combined sensitivity to inhibition of the receptor tyrosine kinase EGFR 2014 Science Signaling 7(351) ra107. DOI: 10.1126/scisignal.2005516
	Bharadwaj, U., et al. Drug-repositioning screening identified piperlongumine as a direct STAT3 inhibitor with potent activity against breast cancer 2015 Oncogene 34(11) 1341-53
	Bieniasz, M., et al. Preclinical Efficacy of Ron Kinase Inhibitors Alone and in Combination with PI3K Inhibitors for Treatment of sfRon-Expressing Breast Cancer Patient-Derived Xenografts 2015 Clinical Cancer Research 21(24) 5588-5600
	Bihani, T., et al. Elacestrant (RAD1901), a Selective Estrogen Receptor Degrader (SERD), Has Antitumor Activity in Multiple ER⁺ Breast Cancer Patient-derived Xenograft Models 2017 Clinical Cancer Research 23(16) 4793-804
	Bockhorn, J., et al. Differentiation and loss of malignant character of spontaneous pulmonary metastases in patient-derived breast cancer models 2014 Cancer Research 74(24) 7406-7417
	Bracker, T. U., et al. Efficacy of MS-275, a selective inhibitor of class I histone deacetylases, in human colon cancer models 2009 International Journal of Oncology 35(4) 909-920
	Brait, M., et al. Comparative mutational landscape analysis of patient-derived tumour xenografts 2017 British Journal of Cancer 116(4) 515-523

	Camarda, R., et al. Inhibition of fatty acid oxidation as a therapy for MYC-overexpressing triple-negative breast cancer 2016 Nature Medicine 22(4) 427-432
	Chartier, C., et al. Therapeutic targeting of tumor-derived r-spondin attenuates b-catenin signaling and tumorigenesis in multiple cancer types 2016 Cancer Research 76(3) 713-723
	Clermont, P. L., et al. Identification of the epigenetic reader CBX2 as a potential drug target in advanced prostate cancer 2016 Clinical Epigenetics 8(16) DOI 10.1186/s13148-016-0182-9
	Clermont, P. L., et al. Polycomb-mediated silencing in neuroendocrine prostate cancer 2015 Clinical Epigenetics 7(1) 40. doi:10.1186/s13148-015-0074-4.
	Cottu, P., et al. Acquired resistance to endocrine treatments is associated with tumor-specific molecular changes in patient-derived luminal breast cancer xenografts 2014 Clinical Cancer Research 20(16) 4314-4325
	da Silveira, W. A., et al. Transcription Factor Networks derived from Breast Cancer Stem Cells control the immune response in the Basal subtype 2017 Scientific Reports 7(1) 2851. doi:10.1038/s41598-017-02761-6.
	D'Amato, N. C., et al. Cooperative dynamics of AR and ER activity in breast cancer 2016 Molecular Cancer Research 14(11) 1054-1067
	de Cremoux, P., et al. HDAC inhibition does not induce estrogen receptor in human triple-negative breast cancer cell lines and patient-derived xenografts 2015 Breast Cancer Research and Treatment 149(1) 81-89
	de Morree, E., et al. Understanding taxanes in prostate cancer; importance of intratumoral drug accumulation 2016 Prostate 76(10) 927-936
	De Plater, L., et al. Predictive gene signature of response to the anti-TweakR mAb PDL192 in patient-derived breast cancer xenografts 2014 PLoS ONE 9(11) e104227. https://doi.org/10.1371/journal.pone.0104227
	Esber, N., et al. Anti-Tumoral Effects of Anti-Progestins in a Patient-Derived Breast Cancer Xenograft Model 2016 Hormones and Cancer 7(2) 137-147
	Euceda, L. R., et al. Metabolic Response to Everolimus in Patient-Derived Triple-Negative Breast Cancer Xenografts 2017 Journal of Proteome Research 16(5) 1868-1879
	Fichtner, I., et al. The experimental antitumor agents phortress and doxorubicin are equiactive against human-derived breast carcinoma xenograft models 2004 Breast Cancer Research and Treatment 87(1) 97-107
	Fischer, M., et al. Anti-DLL4 inhibits growth and reduces tumor-initiating cell frequency in colorectal tumors with oncogenic KRAS mutations 2011 Cancer Research 71(5) 1520-1525
	Gao, H., et al. High-throughput screening using patient-derived tumor xenografts to predict clinical trial drug response 2015 Nature Medicine 21(11) 1318-1325
	Garcia-Garcia, C., et al. Dual mTORC1/2 and HER2 blockade results in antitumor activity in preclinical models of breast cancer resistant to anti-HER2 therapy 2012 Clinical Cancer Research 18(9) 2603-2612
	Garcia-Garcia, C., et al. MEK plus PI3K/mTORC1/2 Therapeutic Efficacy Is Impacted by TP53 Mutation in Preclinical Models of Colorectal Cancer 2015 Clinical Cancer Research 21(24) 5499-5510
	Gardner, E. E., et al. Rapamycin rescues ABT-737 efficacy in small cell lung cancer 2014 Cancer Research 74(10) 2846-2856
	Gardner, E. E., et al. Chemosensitive Relapse in Small Cell Lung Cancer Proceeds through an EZH2-SLFN11 Axis 2017 Cancer Cell 31(2) 286-299
	Giuliano, M., et al. Circulating and disseminated tumor cells from breast cancer patient-derived xenograft-bearing mice as a novel model to study metastasis 2015 Breast Cancer Research 17(3) https://doi.org/10.1186/s13058-014-0508-5
	Grinde, M.T., et al. Interplay of choline metabolites and genes in patient-derived breast cancer xenografts 2014 Breast Cancer Research 16(1) R5
	Gross, M. I., et al. Antitumor activity of the glutaminase inhibitor CB-839 in triple-negative breast cancer 2014 Molecular Cancer Therapeutics 13(4) 890-901
	Gurard-Levin, Z. A., et al. Chromatin Regulators as a Guide for Cancer Treatment Choice 2016 Molecular Cancer Therapeutics 15(7) 1768-77

	Hai, J., et al. Inhibiting MDM2-p53 interaction suppresses tumor growth in patient-derived non-small cell lung cancer xenograft models 2015 Journal of Thoracic Oncology 10(8) 1172-1180
	Hammer, S., et al. Comparative profiling of the novel epothilone, sagopilone, in xenografts derived from primary non-small cell lung cancer 2010 Clinical Cancer Research 16(5) 1452-1465
	Hatem, R., et al. Targeting mTOR pathway inhibits tumor growth in different molecular subtypes of triple-negative breast cancers 2016 Oncotarget 7(30) 48206-48219
	Hatem, R., et al. Vandetanib as a potential new treatment for estrogen receptor-negative breast cancers 2016 International Journal of Cancer 138(10) 2510-2521
	Haukaas, T. H., et al. Impact of freezing delay time on tissue samples for metabolomic studies 2016 Frontiers in Oncology 6(17)<br>doi:10.3389/fonc.2016.00017.
	Hayes, G. M., et al. Antitumor activity of an anti-CD98 antibody 2015 International Journal of Cancer 137(3) 710-720
	Horiuchi, D., et al. PIM1 kinase inhibition as a targeted therapy against triple-negative breast tumors with elevated MYC expression 2016 Nature Medicine 22(11) 1321-1329
	Hylander, B. L., et al. Tumor priming by Apo2L/TRAIL reduces interstitial fluid pressure and enhances efficacy of liposomal gemcitabine in a patient derived xenograft tumor model 2015 Journal of Controlled Release 217: 160-169
	Ibrahim, Y. H., et al. PI3K inhibition impairs BRCA1/2 expression and sensitizes BRCA-proficient triple-negative breast cancer to PARP inhibition 2012 Cancer Discovery 2(11) 1036-1047
	Jdey, W., et al. Micronuclei frequency in tumors is a predictive biomarker for genetic instability and sensitivity to the DNA repair inhibitor AsiDNA 2017 Cancer Research 77(16) 4207-4216
	Kavuri, S. M., et al. HER2 activating mutations are targets for colorectal cancer treatment 2015 Cancer Discovery 5(8) 832-841
	Kim, E., et al. Anti-angiogenic therapy affects the relationship between tumor vascular structure and function: A correlation study between micro-computed tomography angiography and dynamic contrast enhanced MRI 2016 Magnetic Resonance in Medicine: https://doi.org/10.1002/mrm.26547
	Kim, E. Anti-vascular effects of the cytosolic phospholipase A2 inhibitor AVX235 in a patient-derived basal-like breast cancer model 2016 BMC Cancer 16(191) doi:10.1186/s12885-016-2225-1.
	Kirouac, D. C., et al. Clinical responses to ERK inhibition in BRAF^{V600E}-mutant colorectal cancer predicted using a computational model 2017 Npj Systems Biology & Applications 3: 14
	Kopf-Maier, P. and Kestenbach, U. Influence of dispase and angiogenesis factors on the growth of human xenografts 1988 Journal of Cancer Research and Clinical Oncology 114(6) 547-552
	Kristian, A., et al. Positron emission tomography and pharmacokinetics of 2-[18F]-fluoroethyl choline for metabolic studies in breast cancer xenografts 2014 Acta Oncologica 53(8) 1086-1092
	Leconet, W., et al. Therapeutic activity of anti-AXL antibody against triple-negative breast cancer patient-derived xenografts and metastasis 2017 Clinical Cancer Research 23(11) 2806-2816
	Leelatian, N., et al. Single cell analysis of human tissues and solid tumors with mass cytometry 2017 Cytometry Part B - Clinical Cytometry 92(1) 68-78
	Lefort, S., et al. CXCR4 inhibitors could benefit to HER2 but not to triple-negative breast cancer patients 2017 Oncogene 36(9) 1211-1222
	Legrier, M. E., et al. Activation of IFN/STAT1 signalling predicts response to chemotherapy in oestrogen receptor-negative breast cancer 2016 British Journal of Cancer 114(2) 177-187
	Leung, K. M., et al. HER2-specific Modified Fc Fragment (Fcab) induces antitumor effects through degradation of HER2 and apoptosis 2015 Molecular Therapy 23(11) 1722-1733

	Li, G. G., et al. Antitumor activity of RXDX-105 in multiple cancer types with RET rearrangements or mutations 2017 Clinical Cancer Research 23(12) 2981-2990
	Li, Y., et al. SRRM4 Drives Neuroendocrine Transdifferentiation of Prostate Adenocarcinoma Under Androgen Receptor Pathway Inhibition 2017 European Urology 71(1) 68-78
	Lillo, M. A., et al. Bisphenol A Induces Sox2 in ER⁺ Breast Cancer Stem-Like Cells 2017 Hormones and Cancer 8(2) 90-99
	Lin, D., et al. Metabolic heterogeneity signature of primary treatment-naive prostate cancer 2017 Oncotarget 8(16) 25928-25941
	Lo, J. C. Y., et al. Obesity does not promote tumorigenesis of localized patientderived prostate cancer xenografts 2016 Oncotarget 7(30) 47650-47662
	Lok, B. H., et al. PARP Inhibitor activity correlates with slfn11 expression and demonstrates synergy with temozolomide in small cell lung cancer 2017 Clinical Cancer Research 23(2) 523-535
	M, M. Rabet., et al. Nectin-4: A new prognostic biomarker for efficient therapeutic targeting of primary and metastatic triple-negative breast cancer 2017 Annals of Oncology 28(4) 769-776
	Maier, A., et al. Anti-tumor activity of the TGF-beta receptor kinase inhibitor galunisertib (LY2157299 monohydrate) in patient-derived tumor xenografts 2015 Cellular Oncology 38(2) 131–144
	Martin, P., et al. Cetuximab Inhibits T790M-Mediated Resistance to Epidermal Growth Factor Receptor Tyrosine Kinase Inhibitor in a Lung Adenocarcinoma Patient-Derived Xenograft Mouse Model 2016 Clinical Lung Cancer 17(5) 375-383.e2
	McGrail, D. J., et al. Improved prediction of PARP inhibitor response and identification of synergizing agents through use of a novel gene expression signature generation algorithm 2017 Npj Systems Biology & Applications 3: 8 https://doi.org/10.1038/s41540-017-0011-6
	Menghi, F., et al. The tandem duplicator phenotype as a distinct genomic configuration in cancer 2016 Proceedings of the National Academy of Sciences of the United States of America 113(17) E2373-E2382
	Merk, J., et al. Chemoresistance in non-small-cell lung cancer: Can multidrug resistance markers predict the response of xenograft lung cancer models to chemotherapy? 2011 European Journal of Cardio-thoracic Surgery 40(1) e29-e33
	Michaelson, J. S., et al. The anti-Fn14 antibody BIIB036 inhibits tumor growth in xenografts and patient derived primary tumor models and enhances efficacy of chemotherapeutic agents in multiple xenograft models 2012 Cancer Biology and Therapy 13(9) 812-821
	Min, J. W., et al. Identification of distinct tumor subpopulations in lung adenocarcinoma via single-cell RNA-seq 2015 PLoS ONE 10(8) e0135817. https://doi.org/10.1371/journal.pone.0135817
	Mo, F., et al. Stromal Gene Expression is Predictive for Metastatic Primary Prostate Cancer 2017 European Urology: https://doi.org/10.1016/j.eururo.2017.02.038
	Nabavi, N., et al. miR-100-5p inhibition induces apoptosis in dormant prostate cancer cells and prevents the emergence of castration-resistant prostate cancer 2017 Scientific Reports 7: 4079 https://dx.doi.org/10.1038%2Fs41598-017-03731-8
	Nakamura, H., et al. Genistein increases epidermal growth factor receptor signaling and promotes tumor progression in advanced human prostate cancer 2011 PLoS ONE 6(5) e20034. https://doi.org/10.1371/journal.pone.0020034
	Nakamura, H., et al. Genistein versus ICI 182, 780: An ally or enemy in metastatic progression of prostate cancer 2013 Prostate 73(16) 1747-1760
	Neijzen, R., et al. Irinophore CTM, a lipid nanoparticulate formulation of irinotecan, improves vascular function, increases the delivery of sequentially administered 5-FU in HT-29 tumors, and controls tumor growth in patient derived xenografts of colon cancer 2015 Journal of Controlled Release 199: 72-83
	Niazi, F., et al. AMT: Preclinical pharmacology studies 2009 International Journal of Oncology 34(5) 1341-1352
	Ntai, I., et al. Integrated bottom-up and top-down proteomics of patient-derived breast tumor xenografts 2016 Molecular and Cellular Proteomics 15(1) 45-56

	Nunes, M., et al. Evaluating patient-derived colorectal cancer xenografts as preclinical models by comparison with patient clinical data 2015 Cancer Research 75(8) 1560-1566
	Nyquist, M. D., et al. Exploiting AR-regulated drug transport to induce sensitivity to the survivin inhibitor YM155 2017 Molecular Cancer Research 15(5) 521-531
	Ogitani, Y., et al. DS-8201a, a novel HER2-targeting ADC with a novel DNA topoisomerase I inhibitor, demonstrates a promising antitumor efficacy with differentiation from T-DM1 2016 Clinical Cancer Research 22(20) 5097-5108
	Omarjee, S., et al. The molecular mechanisms underlying the ERalpha-36-mediated signaling in breast cancer 2017 Oncogene 36(18) 2503-2514
	Paez-Ribes, M., et al. Development of patient derived xenograft models of overt spontaneous breast cancer metastasis: A cautionary note 2016 PLoS ONE 11(6) e0158034. https://doi.org/10.1371/journal.pone.0158034
	Paez-Ribes, M., et al. Potential proinvasive or metastatic effects of preclinical antiangiogenic therapy are prevented by concurrent chemotherapy 2015 Clinical Cancer Research 21(24) 5488-5498
	Park, J. H., et al. Fatty Acid Oxidation-Driven Src Links Mitochondrial Energy Reprogramming and Oncogenic Properties in Triple-Negative Breast Cancer 2016 Cell Reports 14(9) 2154-2165
	Parolia, A., et al. The long non-coding RNA PCGEM1 is regulated by androgen receptor activity in vivo 2015 Molecular Cancer 14(46) https://doi.org/10.1186/s12943-015-0314-4
	Parra-Palau, J. L., et al. Effect of p95HER2/611CTF on the response to trastuzumab and chemotherapy 2014 Journal of the National Cancer Institute 106(11) https://doi.org/10.1093/jnci/dju291
	Paul, P. J., et al. Restoration of tumor suppression in prostate cancer by targeting the E3 ligase E6AP 2016 Oncogene 35(48) 6235-6245
	Pinthus, J. H., et al. Immuno-gene therapy of established prostate tumors using chimeric receptor-redirected human lymphocytes 2003 Cancer Research 63(10) 2470-2476
	Poirier, J. T., et al. DNA methylation in small cell lung cancer defines distinct disease subtypes and correlates with high expression of EZH2 2015 Oncogene 34(48) 5869-5878
	Qin, X., et al. Gankyrin activates mTORC1 signaling by accelerating TSC2 degradation in colorectal cancer 2016 Cancer Letters 376(1) 83-94
	Qu, S., et al. Enhanced anticancer activity of a combination of docetaxel and Aneustat (OMN54) in a patient-derived, advanced prostate cancer tissue xenograft model 2014 Molecular Oncology 8(2) 311-322
	Rajput, S., et al. Inhibition of cyclin dependent kinase 9 by dinaciclib suppresses cyclin B1 expression and tumor growth in triple negative breast cancer 2016 Oncotarget 7(35) 56864-56875
	Ribas, R., et al. AKT antagonist AZD5363 influences estrogen receptor function in endocrine-resistant breast cancer and synergizes with fulvestrant (ICI182780) in vivo 2015 Molecular Cancer Therapeutics 14(9) 2035-2048
	Rolff, J., et al. Response of patient-derived non-small cell lung cancer xenografts to classical and targeted therapies is not related to multidrug resistance markers 2009 Journal of Oncology: https://doi.org/10.1155/2009/814140
	Rosenberg, L. H., et al. Therapeutic targeting of casein kinase 1delta in breast cancer 2015 Science Translational Medicine 7(318) 318ra202. doi:10.1126/scitranslmed.aac8773.
	Ross, S. J., et al. Targeting KRAS-dependent tumors with AZD4785, a high-affinity therapeutic antisense oligonucleotide inhibitor of KRAS 2017 Science Translational Medicine 9(394) eaal5253 DOI: 10.1126/scitranslmed.aal5253
	Schneeberger, V. E., et al. Quantitation of murine stroma and selective purification of the human tumor component of patient-derived xenografts for genomic analysis 2016 PLoS ONE 11(9) e0160587. https://doi.org/10.1371/journal.pone.0160587
	Serra, V., et al. RSK3/4 mediate resistance to PI3K pathway inhibitors in breast cancer 2013 Journal of Clinical Investigation 123(6) 2551-2563
	Shao, C., et al. Inhibition of polo-like kinase 1 (Plk1) enhances the antineoplastic activity of metformin in prostate cancer 2015 Journal of Biological Chemistry 290(4) 2024-2033

	Shenouda, M. M., et al. Ex vivo expanded natural killer cells from breast cancer patients and healthy donors are highly cytotoxic against breast cancer cell lines and patient-derived tumours 2017 Breast Cancer Research 19(76) https://doi.org/10.1186/s13058-017-0867-9
	Simoes, B. M., et al. Anti-estrogen Resistance in Human Breast Tumors Is Driven by JAG1-NOTCH4-Dependent Cancer Stem Cell Activity 2015 Cell Reports 12(12) 1968-1977
	Skrbo, N., et al. Differential in vivo tumorigenicity of distinct subpopulations from a luminal-like breast cancer xenograft 2014 PLoS ONE 9(11) e113278. https://doi.org/10.1371/journal.pone.0113278
	Smith, M. A., et al. Annotation of human cancers with EGFR signaling-associated protein complexes using proximity ligation assays 2015 Science Signaling 8(359) ra4. doi:10.1126/scisignal.2005906.
	Sommer, A., et al. Preclinical efficacy of the auristatin-based antibody-drug conjugate BAY 1187982 for the treatment of FGFR2-positive solid tumors 2016 Cancer Research 76(21) 6331-6339
	Song, E. K., et al. Potent antitumor activity of cabozantinib, a c-MET and VEGFR2 inhibitor, in a colorectal cancer patient-derived tumor explant model 2015 International Journal of Cancer 136(8) 1967-1975
	Song, W., et al. Targeting EphA2 impairs cell cycle progression and growth of basal-like/triple-negative breast cancers 2017 Oncogene 36: 5620–5630
	Stewart, C. A., et al. Dynamic variations in epithelial-to-mesenchymal transition (EMT), ATM, and SLFN11 govern response to PARP inhibitors and cisplatin in small cell lung cancer 2017 Oncotarget 8(17) 28575-28587
	Stimmer, L., et al. Human breast cancer and lymph node metastases express Gb3 and can be targeted by STxB-vectorized chemotherapeutic compounds 2014 BMC Cancer 14(916) https://doi.org/10.1186/1471-2407-14-916
	Strop, P., et al. RN927C, a site-specific trop-2 antibody-drug conjugate (ADC) with enhanced stability, is highly efficacious in preclinical solid tumor models 2016 Molecular Cancer Therapeutics 15(11) 2698-2708
	Takai, K., et al. Targeting the cancer-associated fibroblasts as a treatment in triple-negative breast cancer 2016 Oncotarget 7(50) 82889-82901
	Tan, W. L., et al. Low cytosine triphosphate synthase 2 expression renders resistance to 5-fluorouracil in colorectal cancer 2011 Cancer Biology and Therapy 11(6) 599-608
	Tentler, J. J., et al. Assessment of the in vivo antitumor effects of ENMD-2076, a novel multitargeted kinase inhibitor, against primary and cell line-derived human colorectal cancer xenograft models 2010 Clinical Cancer Research 16(11) 2989-2998
	Ter Brugge, P., et al. Mechanisms of therapy resistance in patient-derived xenograft models of brca1-deficient breast cancer 2016 Journal of the National Cancer Institute 108(11) https://doi.org/10.1093/jnci/djw148
	Thomas, S. N., et al. Application of quantitative proteomics to the integrated analysis of the ubiquitylated and global proteomes of xenograft tumor tissues 2015 Clinical Proteomics 12(14) https://doi.org/10.1186/s12014-015-9086-5
	Tian, L., et al. Mutual regulation of tumour vessel normalization and immunostimulatory reprogramming 2017 Nature 544: 250–254
	Tripathi, S. C., et al. MCAM mediates chemoresistance in small cell lung cancer via the PI3K/AKT/SOX2 signaling pathway 2017 Cancer Research 77(16) 4414-4425
	Tury, S., et al. Combination of COX-2 expression and PIK3CA mutation as prognostic and predictive markers for celecoxib treatment in breast cancer 2016 Oncotarget 7(51) 85124-85141
	Ue Luk, I. S., et al. BIRC6 targeting as potential therapy for advanced, enzalutamide-resistant prostate cancer 2017 Clinical Cancer Research 23(6) 1542-1551
	Valtorta, S., et al. Metabolic evaluation of non-small cell lung cancer patient-derived xenograft models using ¹⁸F-FDG PET: A potential tool for early therapy response 2017 Journal of Nuclear Medicine 58(1) 42-47
	van der Lee, M. M., et al. The Preclinical Profile of the Duocarmycin-Based HER2-Targeting ADC SYD985 Predicts for Clinical Benefit in Low HER2-Expressing Breast Cancers 2015 Molecular Cancer Therapeutics 14(3) 692-703

	Varkaris, A., et al. Integrating murine and clinical trials with cabozantinib to understand roles of MET and VEGFR2 as targets for growth inhibition of prostate cancer 2016 Clinical Cancer Research 22(1) 107-121
	Vendetti, F. P., et al. Evaluation of azacitidine and entinostat as sensitization agents to cytotoxic chemotherapy in preclinical models of non-small cell lung cancer 2015 Oncotarget 6(1) 56-70
	Vijayaraghavan, S., et al. CDK4/6 and autophagy inhibitors synergistically induce senescence in Rb positive cytoplasmic cyclin e negative cancers 2017 Nature Communications 8: 15916. doi:10.1038/ncomms15916.
	Wallin, J. J., et al. GDC-0941, a novel class I selective PI3K inhibitor, enhances the efficacy of docetaxel in human breast cancer models by increasing cell death In Vitro and In Vivo 2012 Clinical Cancer Research 18(14) 3901-3911
	Wang, D., et al. LncRNA MALAT1 enhances oncogenic activities of EZH2 in castrationresistant prostate cancer 2015 Oncotarget 6(38) 41045-41055
	Wardell, S. E., et al. Efficacy of SERD/SERM Hybrid-CDK4/6 Inhibitor Combinations in Models of Endocrine Therapy-Resistant Breast Cancer 2015 Clinical Cancer Research 21(22) 5121-5130
	Weiswald, L. B., et al. A short-term colorectal cancer sphere culture as a relevant tool for human cancer biology investigation 2013 British Journal of Cancer 108(8) 1720-31
	Welte, T., et al. Oncogenic mTOR signalling recruits myeloid-derived suppressor cells to promote tumour initiation 2016 Nature Cell Biology 18(6) 632-644
	Wu, W., et al. Inhibition of tumor growth and metastasis in non-small cell lung cancer by LY2801653, an inhibitor of several oncokinases, including MET 2013 Clinical Cancer Research 19(20) 5699-5710
	Xie, B. Y. and Wu, A. W. Organoid culture of isolated cells from patient-derived tissues with colorectal cancer 2016 Chinese Medical Journal 129(20) 2469-2475
	Xu, S., et al. Combined targeting of mTOR and AKT Is an effective strategy for basal-like breast cancer in patient-derived xenograft models 2013 Molecular Cancer Therapeutics 12(8) 1665-1675
	Yamamoto, Y., et al. Generation 2.5 antisense oligonucleotides targeting the androgen receptor and its splice variants suppress enzalutamide-resistant prostate cancer cell growth 2015 Clinical Cancer Research 21(7) 1675-1687
	Zacarias-Fluck, M. F., et al. Effect of cellular senescence on the growth of HER2-positive breast cancers 2015 Journal of the National Cancer Institute 107(5) djv020, https://doi.org/10.1093/jnci/djv020
	Zhang, H., et al. Tumor-selective proteotoxicity of verteporfin inhibits colon cancer progression independently of YAP1 2015 Science Signaling 8(397) ra98
	Zhang, X., et al. Breast Cancer Neoantigens Can Induce CD8⁺ T-Cell Responses and Antitumor Immunity 2017 Cancer Immunology Research 5(7) 516-523
	Zhang, Z., et al. PIk1 inhibition enhances the efficacy of androgen signaling blockade in castration-resistant prostate cancer 2014 Cancer Research 74(22) 6635-6647
	Zheng, J., et al. Differential expression of the 18 kDa translocator protein (TSPO) by neoplastic and inflammatory cells in mouse tumors of breast cancer 2011 Molecular Pharmaceutics 8(3) 823-832
	Zhu, X., et al. Epithelial derived CTGF promotes breast tumor progression via inducing EMT and collagen I fibers deposition 2015 Oncotarget 6(28) 25320-25338
	Zinn, R. L., et al. Combination treatment with ABT-737 and chloroquine in preclinical models of small cell lung cancer 2013 Molecular Cancer 12(1) no pagination
	Asundi, J., et al. An Antibody-Drug Conjugate Directed against Lymphocyte Antigen 6 Complex, Locus E (LY6E) Provides Robust Tumor Killing in a Wide Range of Solid Tumor Malignancies. 2015 Clinical cancer research: an official journal of the American Association for Cancer Research 21(14) 3252-62
	Breij, E.C.W., et al. An antibody-drug conjugate that targets tissue factor exhibits potent therapeutic activity against a broad range of solid tumors. 2014 Cancer research 74(4) 1214-26

	Kolev, V. N., et al. PI3K/mTOR dual inhibitor VS-5584 preferentially targets cancer stem cells. 2015 Cancer research 75(2) 446-55
	Snowden, E., et al. Immunophenotyping and Transcriptomic Outcomes in PDX-Derived TNBC Tissue. 2017 Molecular cancer research: MCR 15(4) 429-438
	Sommer, A., et al. Preclinical Efficacy of the Auristatin-Based Antibody-Drug Conjugate BAY 1187982 for the Treatment of FGFR2-Positive Solid Tumors. 2016 Cancer research 76(21) 6331-6339
	Strop, P., et al. RN927C, a Site-Specific Trop-2 Antibody-Drug Conjugate (ADC) with Enhanced Stability, Is Highly Efficacious in Preclinical Solid Tumor Models. 2016 Molecular cancer therapeutics 15(11) 2698-2708
**Exclude [review]**	Brown, K. M., et al. Patient-derived xenograft models of colorectal cancer in preclinical research: A systematic review 2016 Oncotarget 7(40) 66212-66225
	Bruna, A., et al. Modeling breast cancer intertumor and intratumor heterogeneity using xenografts 2016 Cold Spring Harbor Symposia on Quantitative Biology 81(1) 227-230
	Gandara, D. R., et al. Patient-derived xenografts for investigation of acquired resistance in oncogene-driven cancers: Building a better mousetrap 2015 Journal of Clinical Oncology 33(26) 2839-2840
	Grossenbacher, S. K., et al. Natural killer cell immunotherapy to target stem-like tumor cells 2016 Journal for ImmunoTherapy of Cancer 4(19) https://doi.org/10.1186/s40425-016-0124-2
	Jin, K., et al. Patient-derived human tumour tissue xenografts in immunodeficient mice: A systematic review 2010 Clinical and Translational Oncology 12(7) 473-480
	Kitai, H. and Ebi, H. Key roles of EMT for adaptive resistance to MEK inhibitor in KRAS mutant lung cancer 2016 Small GTPases 8(3) 172-176
	Koh, T. S., et al. Assessment of tumor necrotic fraction by dynamic contrast-enhanced MRI: A preclinical study of human tumor xenografts with histopathologic correlation 2014 NMR in Biomedicine 27(4) 486-494
	Merk, J., et al. Patient-derived xenografts of non-small-cell lung cancer: a pre-clinical model to evaluate adjuvant chemotherapy? 2009 European Journal of Cardio-thoracic Surgery 36(3) 454-459
	Brognaro, E. "The development tumor model" to study and monitor the entire progression of both primary and metastatic tumors. 2014 Tumour biology: the journal of the International Society for Oncodevelopmental Biology and Medicine 35(3) 2219-30
	Hoffman, R. M. Patient-derived orthotopic xenografts: better mimic of metastasis than subcutaneous xenografts. 2015 Nature reviews. Cancer 15(8) 451-2
**Exclude [wrong tissue]**	Ab, O., et al. IMGN853, a folate receptor-alpha (FRalpha)-targeting antibody-drug conjugate, exhibits potent targeted antitumor activity against FRalpha-expressing tumors 2015 Molecular Cancer Therapeutics 14(7) 1605-1613
	Astudillo, L., et al. The small molecule IMR-1 inhibits the notch transcriptional activation complex to suppress tumorigenesis 2016 Cancer Research 76(12) 3593-3603
	Bardelli, A., et al. Amplification of the MET receptor drives resistance to anti-EGFR therapies in colorectal cancer 2013 Cancer Discovery 3(6) 658-673
	Bofill-De Ros, X., et al. Late-phase miRNA-controlled oncolytic adenovirus for selective killing of cancer cells 2015 Oncotarget 6(8) 6179-6190
	Bousquet, G., et al. Targeting autophagic cancer stem-cells to reverse chemoresistance in human triple negative breast cancer 2017 Oncotarget 8(21) 35205-35221
	Brand T.M., et al. AXL mediates resistance to cetuximab therapy 2014 Cancer research 74(18) 5152-5164
	Chdiwa, T., et al. Establishment of patient-derived cancer xenografts in immunodeficient NOG mice 2015 International Journal of Oncology 47(1) 61-70
	Cho, Y. B., et al. Colorectal cancer patient-derived xenografted tumors maintain characteristic features of the original tumors 2014 Journal of Surgical Research 187(2) 502-509
	Collins, C. C., et al. Next generation sequencing of prostate cancer from a patient identifies a deficiency of methylthioadenosine phosphorylase, an exploitable tumor target 2012 Molecular Cancer Therapeutics 11(3) 775-781

	Dela Cruz, F. S., et al. A case study of an integrative genomic and experimental therapeutic approach for rare tumors: Identification of vulnerabilities in a pediatric poorly differentiated carcinoma 2016 Genome Medicine 8(116) doi:10.1186/s13073-016-0366-0.
	Ducker, G. S., et al. Incomplete inhibition of phosphorylation of 4E-BP1 as a mechanism of primary resistance to ATP-competitive mTOR inhibitors 2014 Oncogene 33(12) 1590-1600
	Fischer, M. M., et al. WNT antagonists exhibit unique combinatorial antitumor activity with taxanes by potentiating mitotic cell death 2017 Science Advances 3(6) e1700090
	Fleming, J. M., et al. Local regulation of human breast xenograft models 2010 Journal of Cellular Physiology 224(3) 795-806
	Fong, E. L. S., et al. Hydrogel-based 3D model of patient-derived prostate xenograft tumors suitable for drug screening 2014 Molecular Pharmaceutics 11(7) 2040-2050
	Fong, E. L. S., et al. 3D in vitro model of patient-derived prostate cancer xenograft for controlled interrogation of in vivo tumor-stromal interactions 2016 Biomaterials 77: 164-172
	Gad, H., et al. MTH1 inhibition eradicates cancer by preventing sanitation of the dNTP pool 2014 Nature 508(7495) 215-221
	Gan, G. N., et al., A. Radiation dose uncertainty and correction for a mouse orthotopic and xenograft irradiation model 2016 International Journal of Radiation Biology 92(1) 50-56
	Garcia-Heredia, J. M., et al. The Cargo Protein MAP17 (PDZK1IP1) Regulates the Cancer Stem Cell Pool Activating the Notch Pathway by Abducting NUMB 2017 Clinical Cancer Research: DOI: 10.1158/1078-0432.CCR-16-2358
	Goel, S., et al. Overcoming Therapeutic Resistance in HER2-Positive Breast Cancers with CDK4/6 Inhibitors 2016 Cancer Cell 29(3) 255-269
	Goldstein, J. B., et al. Can Microsatellite Status of Colorectal Cancer Be Reliably Assessed after Neoadjuvant Therapy? 2017 Clinical Cancer Research 23(17) 5246–54
	Golfier, S., et al. Anetumab ravtansine: A novel mesothelin-targeting antibody-drug conjugate cures tumors with heterogeneous target expression favored by bystander effect 2014 Molecular Cancer Therapeutics 13(6) 1537-1548
	Goncalves, A., et al. Targeted NGS, array-CGH, and patient-derived tumor xenografts for precision medicine in advanced breast cancer: A single-center prospective study 2016 Oncotarget 7(48) 79428-79441
	Guan, Z., et al. Synergistic effects and mechanism of Danshen injection in combination with chemotherapy and antiangiogenic therapy for colon cancer 2017 International Journal of Clinical and Experimental Medicine 10(4) 6437-6444
	Gujral, T. S. and Kirschner, M. W. Hippo pathway mediates resistance to cytotoxic drugs 2017 Proceedings of the National Academy of Sciences of the United States of America 114(18) E3729-E3738
	Harrison, H., et al. Contrasting hypoxic effects on breast cancer stem cell hierarchy is dependent on er-a status 2013 Cancer Research 73(4) 1420-1433
	Kim, M. K., et al. Characterization of an oxaliplatin sensitivity predictor in a preclinical murine model of colorectal cancer 2012 Molecular Cancer Therapeutics 11(7) 1500-1509
	Kleb, B., et al. Differentially methylated genes and androgen receptor re-expression in small cell prostate carcinomas 2016 Epigenetics 11(3) 184-193
	Ladd, B., et al. Effective combination therapies in preclinical endocrine resistant breast cancer models harboring ER mutations 2016 Oncotarget 7(34) 54120-54136
	LeBeau, A. M., et al. Imaging a functional tumorigenic biomarker in the transformed epithelium 2013 Proceedings of the National Academy of Sciences of the United States of America 110(1) 93-98
	Lillo, M. A., et al. Methylparaben stimulates tumor initiating cells in ER+ breast cancer models 2017 Journal of Applied Toxicology 37(4) 417-425
	Lin, D., et al. Identification of DEK as a potential therapeutic target for neuroendocrine prostate cancer 2015 Oncotarget 6(3) 1806-1820
	Madan, B., et al. Wnt addiction of genetically defined cancers reversed by PORCN inhibition 2016 Oncogene 35(17) 2197-2207
	Nakajima, T., et al. Patient-derived tumor xenograft models established from samples obtained by endobronchial ultrasound-guided transbronchial needle aspiration 2015 Lung Cancer 89(2) 110-114

	Nguyen, A., et al. PKLR promotes colorectal cancer liver colonization through induction of glutathione synthesis 2016 Journal of Clinical Investigation 126(2) 681-694
	Ni, J., et al. Combination inhibition of PI3K and mTORC1 yields durable remissions in mice bearing orthotopic patient-derived xenografts of HER2-positive breast cancer brain metastases 2016 Nature Medicine 22(7) 723-726
	O'Brien, T., et al. Supplementation of nicotinic acid with NAMPT inhibitors results in loss of in vivo efficacy in NAPRT1-deficient tumor models 2013 Neoplasia (United States) 15(12) 1314-1329
	Owonikoko, T. K., et al. Patient-derived xenografts faithfully replicated clinical outcome in a phase II co-clinical trial of arsenic trioxide in relapsed small cell lung cancer 2016 Journal of Translational Medicine 14(111) https://doi.org/10.1186/s12967-016-0861-5
	Pahle, J., et al. Rapid eradication of colon carcinoma by Clostridium perfringens Enterotoxin suicidal gene therapy 2017 BMC Cancer 17(129) https://doi.org/10.1186/s12885-017-3123-x
	Pan, W., et al. Heterogeneity-related anticancer therapy response differences in metastatic colon carcinoma: New hints to tumor-site-based personalized cancer therapy 2013 Hepato-Gastroenterology 60(128) 1927-1934
	Patsialou, A., et al. Intravital multiphoton imaging reveals multicellular streaming as a crucial component of in vivo cell migration in human breast tumors 2013 IntraVital 2(2) e25294, DOI: 10.4161/intv.25294
	Perez, M., et al. Dasatinib, a Src inhibitor, sensitizes liver metastatic colorectal carcinoma to oxaliplatin in tumors with high levels of phospho-Src 2016 Oncotarget 7(22) 33111-33124
	Picco, G., et al. Efficacy of NEDD8 pathway inhibition in preclinical models of poorly differentiated, clinically aggressive colorectal cancer 2017 Journal of the National Cancer Institute 109(2) https://doi.org/10.1093/jnci/djw209
	Porru, M., et al. Targeting G-quadruplex DNA structures by EMICORON has a strong antitumor efficacy against advanced models of human colon cancer 2015 Molecular Cancer Therapeutics 14(11) 2541-2551
	Potter, D. S., et al. Inhibition of PI3K/BMX cell survival pathway sensitizes to BH3 mimetics in SCLC 2016 Molecular Cancer Therapeutics 15(6) 1248-1260
	Proia, D. A., et al. HSP90 inhibitor-SN-38 conjugate strategy for targeted delivery of topoisomerase i inhibitor to tumors 2015 Molecular Cancer Therapeutics 14(11) 2422-2432
	Smoot, R. L., et al. Platelet-Derived Growth Factor Regulates YAP Transcriptional Activity via Src Family Kinase Dependent Tyrosine Phosphorylation 2017 Journal of Cellular Biochemistry 119(1) 10.1002/jcb.26246
	Sun, S., et al. Targeting and regulating of an oncogene via nanovector delivery of MicroRNA using patient-derived tumor xenografts 2017 Theranostics 7(3) 677-693
	Tateishi, K., et al. The alkylating chemotherapeutic temozolomide induces metabolic stress in IDH1-mutant cancers and potentiates NAD+ depletion-mediated cytotoxicity 2017 Cancer Research 77(15) canres.2263.2016. 10.1158/0008-5472.CAN-16-2263.
	Tsai, H., et al. Cyclin D1 Loss Distinguishes Prostatic Small-Cell Carcinoma from Most Prostatic Adenocarcinomas 2015 Clinical Cancer Research 21(24) 5619-5629
	Tung, W. L., et al. Use of irinotecan for treatment of small cell carcinoma of the prostate 2011 Prostate 71(7) 675-681
	Wang, H., et al. The metabolic function of cyclin D3-CDK6 kinase in cancer cell survival 2017 Nature 546(7658) 426-430
	Zanella, E. R., et al. IGF2 is an actionable target that identifies a distinct subpopulation of colorectal cancer patients with marginal response to anti-EGFR therapies 2015 Science Translational Medicine 7(272) 272ra12
	Abelson, S., et al. Niche-dependent gene expression profile of intratumoral heterogeneous ovarian cancer stem cell populations. 2013 PloS one 8(12) e83651
	Alvero, A. B., et al. Stem-like ovarian cancer cells can serve as tumor vascular progenitors. 2009 Stem cells (Dayton, Ohio) 27(10) 2405-13
	Antunes, L., et al. Analysis of tissue chimerism in nude mouse brain and abdominal xenograft models of human glioblastoma multiforme: what does it tell us about the models and about glioblastoma biology and therapy? 2000 The journal of histochemistry and cytochemistry: 48(6) 847-58

	Bankert, R.B., et al. Humanized mouse model of ovarian cancer recapitulates patient solid tumor progression, ascites formation, and metastasis. 2011 PloS one 6(9) e24420
	Boult, J.K.R., et al. Preclinical transgenic and patient-derived xenograft models recapitulate the radiological features of human adamantinomatous craniopharyngioma. 2017 Brain pathology (Zurich, Switzerland) 28(4) 475-483
	Calabuig-FariÃ±as, S., et al. Characterization of a new human cell line (CH-3573) derived from a grade II chondrosarcoma with matrix production. 2012 Pathology oncology research: POR 18(4) 793-802
	Emura, F., et al. Establishment and characterization of novel xenograft models of human biliary tract carcinomas. 2003 International journal of oncology 23(5) 1293-300
	Friel, A. M., et al. Epigenetic regulation of CD133 and tumorigenicity of CD133 positive and negative endometrial cancer cells. 2010 Reproductive biology and endocrinology: RB&E 8: 147
	Garcia, P.L., et al. Development and histopathological characterization of tumorgraft models of pancreatic ductal adenocarcinoma. 2013 PloS one 8(10) e78183
	He, X., et al. Differentiation of a highly tumorigenic basal cell compartment in urothelial carcinoma. 2009 Stem cells (Dayton, Ohio) 27(7) 1487-95
	Holmberg, J., et al. Activation of neural and pluripotent stem cell signatures correlates with increased malignancy in human glioma. 2011 PloS one 6(3) e18454
	Huynh, H., et al. Sunitinib (SUTENT, SU11248) suppresses tumor growth and induces apoptosis in xenograft models of human hepatocellular carcinoma. 2009 Current cancer drug targets 9(6) 738-47
	Isobe, N., et al. Experimental studies of the antitumor effect of TNP-470 on malignant brain tumors. Antitumor effect of TNP-470 on a human medulloblastoma xenograft line. 1996 Neuropediatrics 27(3) 136-42
	Jacobsen, Britta M., et al. Spontaneous fusion with, and transformation of mouse stroma by, malignant human breast cancer epithelium. 2006 Cancer research 66(16) 8274-9
	Jambal, P., et al. Estrogen switches pure mucinous breast cancer to invasive lobular carcinoma with mucinous features. 2013 Breast cancer research and treatment 137(2) 431-48
	Jensen, R L., et al. Matrigel augments xenograft transplantation of meningioma cells into athymic mice. 1998 Neurosurgery 42(1) 130-5; discussion 135-6
	Krasagakis, K., et al. Growth and characterization of a cell line from a human primary neuroendocrine carcinoma of the skin (Merkel cell carcinoma) in culture and as xenograft. 2001 Journal of cellular physiology 187(3) 386-91
	Lewko, W M., et al. Cultured cell lines from human breast cancer biopsies and xenografts. 1990 Breast cancer research and treatment 17(2) 121-9
	Luo, Y., et al. Side population cells from human melanoma tumors reveal diverse mechanisms for chemoresistance. 2012 The Journal of investigative dermatology 132(10) 2440-50
	Moserle, L., et al. The side population of ovarian cancer cells is a primary target of IFN-alpha antitumor effects. 2008 Cancer research 68(14) 5658-68
	Nolte, Sara M., et al. A cancer stem cell model for studying brain metastases from primary lung cancer. 2013 Journal of the National Cancer Institute 105(8) 551-62
	Orecchia, P., et al. Targeting Syndecan-1, a molecule implicated in the process of vasculogenic mimicry, enhances the therapeutic efficacy of the L19-IL2 immunocytokine in human melanoma xenografts. 2015 Oncotarget 6(35) 37426-42
	Pode-Shakked, N. et al., The isolation and characterization of renal cancer initiating cells from human Wilms' tumour xenografts unveils new therapeutic targets. 2013 EMBO molecular medicine 5(1) 18-37
	Rahimi, G., et al. Re-vascularisation in human ovarian tissue after conventional freezing or vitrification and xenotransplantation. 2010 European journal of obstetrics, gynecology, and reproductive biology 149(1) 63-7
	Rath, K.S., et al. HO-3867, a safe STAT3 inhibitor, is selectively cytotoxic to ovarian cancer. 2014 Cancer research 74(8) 2316-27
	Rocco, A., et al. CD133 and CD44 cell surface markers do not identify cancer stem cells in primary human gastric tumors. 2012 Journal of cellular physiology 227(6) 2686-93

	Singh, S.K., et al. Identification of human brain tumour initiating cells. 2004 Nature 432(7015) 396-401
	Steinstraesser, L., et al. Establishment of a primary human sarcoma model in athymic nude mice. 2010 Human cell 23(2) 50-7
	Sun, K., et al. Dual HDAC and PI3K Inhibitor CUDC-907 Downregulates MYC and Suppresses Growth of MYC-dependent Cancers. 2017 Molecular cancer therapeutics 16(2) 285-299
	Taetle, R., et al. Use of nude mouse xenografts as preclinical screens. Characterization of xenograft-derived melanoma cell lines. 1987 Cancer 60(8) 1836-41
	Tilkorn, D., et al. Establishing efficient xenograft models with intrinsic vascularisation for growing primary human low-grade sarcomas. 2011 Anticancer research 31(12) 4061-6
	Tilkorn, D-J., et al. A novel xenograft model with intrinsic vascularisation for growing undifferentiated pleomorphic sarcoma NOS in mice. 2012 Journal of cancer research and clinical oncology 138(5) 877-84
	Ueno, M., et al. Establishment of a testicular carcinoma cell line producing alpha-fetoprotein. 2001 BJU international 88(6) 611-21
	van Haaften-Day, C., et al. Flow cytometric and morphological studies of ovarian carcinoma cell lines and xenografts. 1983 Cancer research 43(8) 3725-31
	Wagner, W. The heterogeneity of solid human xenotransplant tumours. 1991 Journal of cancer research and clinical oncology 117(6) 568-74
	Wang, CYY., et al. Hedgehog and Notch signaling regulate self-renewal of undifferentiated pleomorphic sarcomas. 2012 Cancer research 72(4) 1013-22
	Waters, A.M., et al. A Preclinical Evaluation of UAB30 in Pediatric Renal and Hepatic Malignancies. 2016 Molecular cancer therapeutics 15(5) 911-21
	Wu, C-P., et al. Identification of cancer stem-like side population cells in purified primary cultured human laryngeal squamous cell carcinoma epithelia. 2013 PloS one 8(6) e65750
	Yapp, D.T., et al. The differential effects of metronomic gemcitabine and antiangiogenic treatment in patient-derived xenografts of pancreatic cancer: treatment effects on metabolism, vascular function, cell proliferation, and tumor growth. 2016 Angiogenesis 19(2) 229-44
	Yi, D., et al. EGFR gene overexpression retained in an invasive xenograft model by solid orthotopic transplantation of human glioblastoma multiforme into nude mice. 2011 Cancer investigation 29(3) 229-39
	Zamboni, W C., et al. Relationship between topotecan systemic exposure and tumor response in human neuroblastoma xenografts. 1998 Journal of the National Cancer Institute 90(7) 505-11
	Zhu, G., et al. Comparison of two kinds of orthotopic xenograft models for human ovarian cancer. 2014 European journal of gynaecological oncology 35(6) 724-7
**Include [no validation]**	Airley, R. E., et al. Hypoxia-regulated glucose transporter Glut-1 may influence cheity to some alkylating: results of EORTC (First Translational Award) study of the relevance of tumour hypoxia to the outcome of chemotherapy in human tumour-derived xenografts 2005 International journal of oncology 26(6) 1477-1484
	Aloia, A., et al. The sialyl-glycolipid stage-specific embryonic antigen 4 marks a subpopulation of chemotherapy-resistant breast cancer cells with mesenchymal features 2015 Breast Cancer Research 17: doi: 10.1186/s13058-015-0652-6
	Baba, Y., et al. Panitumumab interaction with TAS-102 leads to combinational anticancer effects via blocking of EGFR-mediated tumor response to trifluridine 2017 Molecular Oncology 11(8) 1065-1077
	Benetatos, C. A., et al. Birinapant (TL32711), a bivalent SMAC mimetic, targets TRAF2-associated cIAPs, abrogates TNF-induced NF-kappabeta activation, and is active in patient-derived xenograft models 2014 Molecular Cancer Therapeutics 13(4) 867-879
	Bertolini, G., et al. Microenvironment-modulated metastatic CD133⁺/CXCR4⁺/EpCAM⁻lung cancer-initiating cells sustain tumor dissemination and correlate with poor prognosis 2015 Cancer Research 75(17) 3636-3649

	Bieche, I., et al. Vasculature analysis of patient derived tumor xenografts using species-specific PCR assays: Evidence of tumor endothelial cells and atypical VEGFA-VEGFR1/2 signalings 2014 BMC Cancer 14: 178
	Blakely, C. M., et al. NF-kappaB-Activating complex engaged in response to EGFR oncogene inhibition drives tumor cell survival and residual disease in lung cancer 2015 Cell Reports 11(1) 98-110
	Bonanno, L., et al. LKB1 Expression Correlates with Increased Survival in Patients with Advanced Non-Small Cell Lung Cancer Treated with Chemotherapy and Bevacizumab 2017 Clinical Cancer Research 23(13) 3316-3324
	Bosch, A., et al. PI3K inhibition results in enhanced estrogen receptor function and dependence in hormone receptor-positive breast cancer 2015 Science Translational Medicine 7(283) 283ra51. doi:10.1126/scitranslmed.aaa4442.
	Bradford, J. R., et al. Whole transcriptome profiling of patient-derived xenograft models as a tool to identify both tumor and stromal specific biomarkers 2016 Oncotarget 7(15) 20773-20787
	Bradshaw-Pierce, E. L., et al. Tumor P-glycoprotein correlates with efficacy of PF-3758309 in in vitro and in vivo models of colorectal cancer 2013 Frontiers in Pharmacology 4(22) doi:10.3389/fphar.2013.00022.
	Braso-Maristany, F., et al. PIM1 kinase regulates cell death, tumor growth and chemotherapy response in triple-negative breast cancer 2016 Nature Medicine 22(11) 1303-1313
	Breij, E. C., et al. An antibody-drug conjugate that targets tissue factor exhibits potent therapeutic activity against a broad range of solid tumors 2014 Cancer Research 74(4) 1214-26
	Brunker, P., et al. RG7386, a novel tetravalent FAP-DR5 antibody, effectively triggers FAP-dependent, avidity-driven DR5 hyperclustering and tumor cell apoptosis 2016 Molecular Cancer Therapeutics 15(5) 946-957
	Butler, D. E., et al. Inhibition of the PI3K/AKT/mTOR pathway activates autophagy and compensatory Ras/Raf/MEK/ERK signalling in prostate cancer 2017 Oncotarget 23(8) 56698-56713
	Chao, C., et al. Cystathionine-beta-synthase inhibition for colon cancer: Enhancement of the efficacy of aminooxyacetic acid via the prodrug approach 2016 Molecular Medicine 22: 361-379
	Chen, E. C., et al. KIT Signaling Promotes Growth of Colon Xenograft Tumors in Mice and Is Up-Regulated in a Subset of Human Colon Cancers 2015 Gastroenterology 149(3) 705e2-717e2
	Chen, H. J., et al. Comprehensive models of human primary and metastatic colorectal tumors in immunodeficient and immunocompetent mice by chemokine targeting 2015 Nature Biotechnology 33(6) 656-660
	Chen, X., et al. Preclinical activity of the rational combination of apatinib in combination with CPT-11 in KRAS-mutant colorectal cancer patient-derived xenograft model 2017 International Journal of Clinical and Experimental Medicine 10(2) 2274-2284
	Chiron, M., et al. Differential antitumor activity of aflibercept and bevacizumab in patient-derived xenograft models of colorectal cancer 2014 Molecular Cancer Therapeutics 13(6) 1636-1644
	Coussy, F., et al. Clinical value of R-spondins in triple-negative and metaplastic breast cancers 2017 British Journal of Cancer 116(12) 1595-1603
	Dalm, S. U., et al. SSTR-mediated Breast Cancer Imaging: Is There A Role For Radiolabeled SSTR Antagonists? 2017 Journal of Nuclear Medicine 58(10) 1609-1614
	Damelin, M., et al. A PTK7-targeted antibody-drug conjugate reduces tumor-initiating cells and induces sustained tumor regressions 2017 Science Translational Medicine 9(372) eaag2611 DOI: 10.1126/scitranslmed.aag2611
	Damelin, M., et al. Delineation of a cellular hierarchy in lung cancer reveals an oncofetal antigen expressed on tumor-initiating cells 2011 Cancer Research 71(12) 4236-4246
	Dave, B., et al. Role of RPL39 in Metaplastic Breast Cancer 2017 Journal of the National Cancer Institute 109(6) djw292, https://doi.org/10.1093/jnci/djw292

	Dave, B., et al. Targeting RPL39 and MLF2 reduces tumor initiation and metastasis in breast cancer by inhibiting nitric oxide synthase signaling 2014 Proceedings of the National Academy of Sciences of the United States of America 111(24) 8838-8843
	Delgado San Martin, J. A., et al. Tumour stromal morphology impacts nanomedicine cytotoxicity in patient-derived xenografts 2015 Nanomedicine: Nanotechnology, Biology, and Medicine 11(5) 1247-1252
	Ding, X. F., et al. Validation of p27KIP1 expression levels as a candidate predictive biomarker of response to rapalogs in patient-derived breast tumor xenografts 2015 Tumor Biology 36: 1463. https://doi.org/10.1007/s13277-014-2580-y
	Dong, X., et al. Antitumor effect of FP3 in combination with cetuximab on patient-derived tumor tissue xenograft models of primary colon carcinoma and related lymphatic and hepatic metastases 2012 International Journal of Molecular Medicine 30(1) 126-132
	Du, L., et al. Role of SUMO activating enzyme in cancer stem cell maintenance and self-renewal 2016 Nature Communications 7: 12326. doi:10.1038/ncomms12326.
	Elsarraj, H. S., et al. NEMO, a Transcriptional Target of Estrogen and Progesterone, Is Linked to Tumor Suppressor PML in Breast Cancer 2017 Cancer Research 77(14) 3802-3813
	Faber, A. C., et al. Assessment of ABT-263 activity across a cancer cell line collection leads to a potent combination therapy for small-cell lung cancer 2015 Proceedings of the National Academy of Sciences of the United States of America 112(11) E1288-E1296
	Fan, F., et al. The requirement for freshly isolated human colorectal cancer (CRC) cells in isolating CRC stem cells 2015 British Journal of Cancer 112(3) 539-546
	Fichtner, I., et al. Establishment of patient-derived non-small cell lung cancer xenografts as models for the identification of predictive biomarkers 2008 Clinical Cancer Research 14(20) 6456-6468
	Forest, A., et al. Intrinsic resistance to cixutumumab is conferred by distinct isoforms of the insulin receptor 2015 Molecular Cancer Research 13(12) 1615-1626
	Friese-Hamim, M., et al. The selective c-Met inhibitor tepotinib can overcome epidermal growth factor receptor inhibitor resistance mediated by aberrant c-Met activation in NSCLC models 2017 American Journal of Cancer Research 7(4) 962-972
	Fu, C. H., et al. A novel oncogenic role of inositol phosphatase SHIP2 in ER-negative breast cancer stem cells: Involvement of JNK/Vimentin activation 2014 Stem Cells 32(8) 2048-2060
	Fujii, E., et al. The status of donor cancer tissues affects the fate of patient-derived colorectal cancer xenografts in NOG mice 2014 Experimental Animals 64(2) 181-190
	Gao, M., et al. Expression analysis and clinical significance of eIF4E, VEGF-C, E-cadherin and MMP-2 in colorectal adenocarcinoma 2016 Oncotarget 7(51) 85502-85514
	Gaponova, A. V., et al. A novel HSP90 inhibitor-drug conjugate to SN38 is highly effective in small cell lung cancer 2016 Clinical Cancer Research 22(20) 5120-5129
	Goetz, M. P., et al. Tumor Sequencing and Patient-Derived Xenografts in the Neoadjuvant Treatment of Breast Cancer 2017 Journal of the National Cancer Institute 109(7) djw306. doi:10.1093/jnci/djw306.
	Guan, Z., et al. Optimizing dosage regimen for FP3 based on vascular normalization window monitored by ratio of Ang-1 to Ang-2 2017 International Journal of Clinical and Experimental Medicine 10(2) 2156-2164
	Guerrera, F., et al. The influence of tissue ischemia time on RNA integrity and patient-derived xenografts (PDX) engraftment rate in a non-small cell lung cancer (NSCLC) Biobank 2016 PLoS ONE 11(1) e0145100. doi:10.1371/journal.pone.0145100.
	Guo, S., et al. Cetuximab response in CRC patient-derived xenografts seems predicted by an expression-based Ras pathway signature 2016 Oncotarget 7(31) 50575-50581
	Guo, S., et al. Molecular pathology of patient tumors, patient-derived xenografts, and cancer cell lines 2016 Cancer Research 76(16) 4619-4626
	Hall, A. B., et al. Potentiation of tumor responses to DNA damaging therapy by the selective ATR inhibitor VX-970 2014 Oncotarget 5(14) 5674-5685
	Han, Z., et al. Inhibition of STAT3 signaling targets both tumor-initiating and differentiated cell populations in prostate cancer 2014 Oncotarget 5(18) 8416-8428

	Herrera-Abreu, M. T., et al. Early adaptation and acquired resistance to CDK4/6 inhibition in estrogen receptor-positive breast cancer 2016 Cancer Research 76(8) 2301-2313
	Hiroshima, Y., et al. Successful fluorescence-guided surgery on human colon cancer patient-derived orthotopic xenograft mouse models using a fluorophore-conjugated anti-CEA antibody and a portable imaging system 2014 Journal of Laparoendoscopic and Advanced Surgical Techniques 24(4) 241-247
	Holton, A. B., et al. Microfluidic biopsy trapping device for the real-time monitoring of tumor microenvironment 2017 PLoS ONE 12(1) e0169797. doi:10.1371/journal.pone.0169797.
	Hu, S., et al. Antagonism of EGFR and Notch limits resistance to EGFR inhibitors and radiation by decreasing tumor-initiating cell frequency 2017 Science Translational Medicine 9(380) eaag0339 DOI: 10.1126/scitranslmed.aag0339
	Hu, Y., et al. PARP1-driven poly-ADP-ribosylation regulates BRCA1 function in homologous recombination-mediated DNA repair 2014 Cancer Discovery 4(12) 1430-1447
	Hu, Y., et al. Fibroblast-derived exosomes contribute to chemoresistance through priming cancer stem cells in colorectal cancer 2015 PLoS ONE 10(5) e0125625. https://doi.org/10.1371/journal.pone.0125625
	Imamura, Y., et al. Comparison of 2D- and 3D-culture models as drug-testing platforms in breast cancer 2015 Oncology Reports 33(4) 1837-1843
	Ionkina, A. A., et al. Efficacy and molecular mechanisms of differentiated response to the Aurora and angiogenic kinase inhibitor ENMD-2076 in preclinical models of p53-mutated triple-negative breast cancer 2017 Frontiers in Oncology 7: 94
	Isella, C., et al. Selective analysis of cancer-cell intrinsic transcriptional traits defines novel clinically relevant subtypes of colorectal cancer 2017 Nature Communications 8: 15107
	Jia, Y., et al. EGF816 exerts anticancer effects in non-small cell lung cancer by irreversibly and selectively targeting primary and acquired activating mutations in the EGF receptor 2016 Cancer Research 76(6) 1591-1602
	Johnson, S. F., et al. CDK12 Inhibition Reverses De Novo and Acquired PARP Inhibitor Resistance in BRCA Wild-Type and Mutated Models of Triple-Negative Breast Cancer 2016 Cell Reports 17(9) 2367-2381
	Karagiannis, G. S., et al. Neoadjuvant chemotherapy induces breast cancer metastasis through a TMEM-mediated mechanism 2017 Science Translational Medicine 9(397) 5
	Ketao, J., et al. Differential response to EGFR- And VEGF-targeted therapies in patient-derived tumor tissue xenograft models of colon carcinoma and related metastases 2012 International Journal of Oncology 41(2) 583-588
	Kim, H. Y., et al. Evaluation of anti-tumorigenic activity of BP3B against colon cancer with patient-derived tumor xenograft model 2016 BMC Complementary and Alternative Medicine 16: 473
	Kim, J., et al. Heterogeneous perivascular cell coverage affects breast cancer metastasis and response to chemotherapy 2016 Jci Insight 1(21) e90733
	Kroon, P., et al. JAK-STAT blockade inhibits tumor initiation and clonogenic recovery of prostate cancer stem-like cells 2013 Cancer Research 73(16) 5288-5298
	Kung, P. P., et al. Chemogenetic evaluation of the mitotic kinesin CENP-E reveals a critical role in triple-negative breast cancer 2014 Molecular Cancer Therapeutics 13(8) 2104-2115
	Lee, K. J., et al. Application of peptide displaying phage as a novel diagnostic probe for human lung adenocarcinoma 2016 Amino Acids 48(4) 1079-1086
	Lee, M. S., et al. Efficacy of the combination of MEK and CDK4/6 inhibitors in vitro and in vivo in KRAS mutant colorectal cancer models 2016 Oncotarget 7(26) 39595-39608
	Lee, S. C., et al. Essential role of insulin-like growth factor 2 in resistance to histone deacetylase inhibitors 2016 Oncogene 35(42) 5515-5526
	Li, C., et al. CD54-NOTCH1 axis controls tumor initiation and cancer stem cell functions in human prostate cancer 2017 Theranostics 7(1) 67-80
	Li, J., et al. Comparing Patient-Derived Xenograft and Computational Response Prediction for Targeted Therapy in Patients of Early-Stage Large Cell Lung Cancer 2016 Clinical Cancer Research 22(9) 2167-2176
	Li, R., et al. Modulation of Bax and mTOR for cancer therapeutics 2017 Cancer Research 77(11) 3001-3012

	Lieu, C. H., et al. Antitumor activity of a potent MEK inhibitor, TAK-733, against colorectal cancer cell lines and patient derived xenografts 2015 Oncotarget 6(33) 34561-34572
	Lim, D. Y., et al. A natural small molecule, catechol, induces c-Myc degradation by directly targeting ERK2 in lung cancer 2016 Oncotarget 7(23) 35001-35014
	Liu, J., et al. Lung cancer tumorigenicity and drug resistance are enhanced through ALDHN^{hi}CD44^{hi} tumor initiating cells 2013 Oncotarget 4(10) 1686-1699
	Liu, R., et al. Mifepristone suppresses basal triple-negative breast cancer stem cells by down-regulating KLF5 expression 2016 Theranostics 6(4) 533-544
	Lopez-Albaitero, A., et al. Overcoming resistance to HER2-targeted therapy with a novel HER2/CD3 bispecific antibody 2017 OncoImmunology 6(3) DOI: 10.1080/2162402X.2016.1267891
	Manuel, C. A., et al. Procedure for horizontal transfer of patient-derived xenograft tumors to eliminate corynebacterium bovis 2017 Journal of the American Association for Laboratory Animal Science 56(2) 166-172
	Mattern, J., et al. Occurrence of a multidrug-resistant phenotype in human lung xenografts 1987 British Journal of Cancer 56(4) 407-411
	Mattie, M., et al. The discovery and preclinical development of ASG-5ME, an antibody-drug conjugate targeting SLC44A4-positive epithelial tumors including pancreatic and prostate cancer 2016 Molecular Cancer Therapeutics 15(11) 2679-2687
	Metildi, C. A. and Kaushal, S. and Luiken, G. A. and Talamini, M. A. and Hoffman, R. M. and Bouvet, M. Fluorescently labeled chimeric anti-CEA antibody improves detection and resection of human colon cancer in a patient-derived orthotopic xenograft (PDOX) nude mouse model 2014 Journal of Surgical Oncology 109(5) 451-458
	Min, H. Y., et al. Essential role of DNA methyltransferase 1-mediated transcription of insulin-like growth factor 2 in resistance to histone deacetylase inhibitors 2017 Clinical Cancer Research 23(5) 1299-1311
	Min, H. Y., et al. Targeting the insulin-like growth factor receptor and Src signaling network for the treatment of non-small cell lung cancer 2015 Molecular Cancer 14: 113. doi:10.1186/s12943-015-0392-3.
	Misale, S., et al. Vertical suppression of the EGFR pathway prevents onset of resistance in colorectal cancers 2015 Nature Communications 6: e0135817. https://doi.org/10.1371/journal.pone.0135817
	Moro, M., et al. Combination treatment with all-trans retinoic acid prevents cisplatin-induced enrichment of CD133⁺ tumor-initiating cells and reveals heterogeneity of cancer stem cell compartment in lung cancer 2015 Journal of Thoracic Oncology 10(7) 1027-1036
	Murone, M., et al. 0617B inhibits growth of STAT3-driven solid tumors through combined inhibition of JAK, SRC, and class III/V receptor tyrosine kinases 2016 Molecular Cancer Therapeutics 15(10) 2334-2343
	Nwogu, C., et al. Photodynamic therapy of human lung cancer xenografts in mice 2016 Journal of Surgical Research 200(1) 8-12
	Paquet, C., et al. Effect of anti-cancer drugs on microglia in patient-derived breast cancer xenografted mouse models 2017 Neuropathology 37(1) 91-93
	Pearson, A., et al. High-level clonal FGFR amplification and response to FGFR inhibition in a translational clinical trial 2016 Cancer Discovery 6(8) 838-851
	Perera, T. P. S., et al. Discovery & pharmacological characterization of JNJ-42756493 (Erdafitinib), a functionally selective small-molecule FGFR family inhibitor 2017 Molecular Cancer Therapeutics 16(6) 1010-1020
	Pitts, T. M., et al. Dual pharmacological targeting of the map kinase and pi3k/mtor pathway in preclinical models of colorectal cancer 2014 PLoS ONE 9(11) e113037. https://doi.org/10.1371/journal.pone.0113037
	Pitts, T. M., et al. Development of an integrated genomic classifier for a novel agent in colorectal cancer: Approach to individualized therapy in early development 2010 Clinical Cancer Research 16(12) 3193-3204

	Qiu, M., et al. Specific inhibition of Notch1 signaling enhances the antitumor efficacy of chemotherapy in triple negative breast cancer through reduction of cancer stem cells 2013 Cancer Letters 328(2) 261-270
	Quackenbush, K. S., et al. The novel tankyrase inhibitor (AZ1366) enhances irinotecan activity in tumors that exhibit elevated tankyrase and irinotecan resistance 2016 Oncotarget 7(19) 28273-28285
	Reno, T. A., et al. The triptolide derivative MRx102 inhibits Wnt pathway activation and has potent anti-tumor effects in lung cancer 2016 BMC Cancer 16(439) https://dx.doi.org/10.1186%2Fs12885-016-2487-7
	Rolff, J., et al. Preclinical Study of a Combination of Erlotinib and Bevacizumab in Early Stages of Unselected Non-Small Cell Lung Cancer Patient-Derived Xenografts 2016 Targeted Oncology 11(4) 507-514
	Rothwell, D. G., et al. Evaluation and validation of a robust single cell RNA-amplification protocol through transcriptional profiling of enriched lung cancer initiating cells 2014 BMC Genomics 15(1129) https://doi.org/10.1186/1471-2164-15-1129
	Ruggles, K. V., et al. An analysis of the sensitivity of proteogenomic mapping of somatic mutations and novel splicing events in cancer 2016 Molecular and Cellular Proteomics 15(3) 1060-1071
	Sai, J., et al. PI3K Inhibition Reduces Mammary Tumor Growth and Facilitates Antitumor Immunity and Anti-PD1 Responses 2017 Clinical Cancer Research 23(13) 3371-3384
	Salvador, M. A. et al., et al. The histone deacetylase inhibitor abexinostat induces cancer stem cells differentiation in breast cancer with low Xist expression 2013 Clinical Cancer Research 19(23) 6520-31
	Sandercock, A. M., et al. Identification of anti-tumour biologics using primary tumour models, 3-D phenotypic screening and image-based multi-parametric profiling 2015 Molecular Cancer 14: 147
	Santag, S., et al. BAY 1143269, a novel MNK1 inhibitor, targets oncogenic protein expression and shows potent anti-tumor activity 2017 Cancer Letters 390: 21-29
	Sapra, P., et al. Long-term tumor regression induced by an antibody-drug conjugate that targets 5T4, an oncofetal antigen expressed on tumor-initiating cells 2013 Molecular Cancer Therapeutics 12(1) 38-47
	Schmieder, R., et al. Regorafenib (BAY 73-4506): Antitumor and antimetastatic activities in preclinical models of colorectal cancer 2014 International Journal of Cancer 135(6) 1487-1496
	Seguin, L., et al. An integrin beta 3-KRAS-RalB complex drives tumour stemness and resistance to EGFR inhibition 2014 Nature Cell Biology 16(5) 457-468
	Shao, H., et al. Improved response to nab-paclitaxel compared with cremophor-solubilized paclitaxel is independent of secreted protein acidic and rich in cysteine expression in non-small cell lung cancer 2011 Journal of Thoracic Oncology 6(6) 998-1005
	Sharma, S. K., et al. Noninvasive Interrogation of DLL3 Expression in Metastatic Small Cell Lung Cancer 2017 Cancer Research 77(14) 3931-3941
	Spreafico, A., et al. Rational combination of a MEK inhibitor, selumetinib, and the wnt/calcium pathway modulator, cyclosporin a, in preclinical models of colorectal cancer 2013 Clinical Cancer Research 19(15) 4149-4162
	Streeter, J. E., et al. A comparative evaluation of ultrasound molecular imaging, perfusion imaging, and volume measurements in evaluating response to therapy in patient-derived xenografts 2013 Technology in Cancer Research and Treatment 12(4) 311-321
	Tentler, J. J., et al. P53 family members regulate phenotypic response to Aurora kinase a inhibition in Triple-negative breast cancer 2015 Molecular Cancer Therapeutics 14(5) 1117-1129
	Thomas, M. L., et al. Citral reduces breast tumor growth by inhibiting the cancer stem cell marker ALDH1A3 2016 Molecular Oncology 10(9) 1485-1496
	Tignanelli, C. J., et al. KRAS and PIK3CA mutation frequencies in patient-derived xenograft models of pancreatic and colorectal cancer are reflective of patient tumors and stable across passages 2014 American Surgeon 80(9) 873-877
	Tominaga, K., et al. Addiction to the IGF2-ID1-IGF2 circuit for maintenance of the breast cancer stem-like cells 2017 Oncogene 36(9) 1276-1286

	Ugolkov, A., et al. GSK-3 inhibition overcomes chemoresistance in human breast cancer 2016 Cancer Letters 380(2) 384-392
	Wang, K., et al. PEST domain mutations in Notch receptors comprise an oncogenic driver segment in triple-negative breast cancer sensitive to a gamma-secretase inhibitor 2015 Clinical Cancer Research 21(6) 1487-1496
	Wong, H., et al. Pharmacokinetic-pharmacodynamic analysis of vismodegib in preclinical models of mutational and ligand-dependent Hedgehog pathway activation 2011 Clinical Cancer Research 17(14) 4682-4692
	Wright, P. K., et al. 17beta-estradiol regulates giant vesicle formation via estrogen receptor-alpha in human breast cancer cells 2014 Oncotarget 5(10) 3055-3065
	Xiao, Y., et al. Dependence of tumor cell lines and patient-derived tumors on the NAD salvage pathway renders them sensitive to NAMPT inhibition with GNE-618 2013 Neoplasia (New York) 15(10) 1151-60
	Xu, H., et al. CX-5461 is a DNA G-quadruplex stabilizer with selective lethality in BRCA1/2 deficient tumours 2017 Nature Communications 8: http://dx.doi.org/10.1038/ncomms14432
	Xu, Y. C., et al. Integration of receptor tyrosine kinases determines sensitivity to PI3Kalpha-selective inhibitors in breast cancer 2017 Theranostics 7(4) 974-986
	Ye, X. S., et al. A novel Eg5 inhibitor (LY2523355) causes mitotic arrest and apoptosis in cancer cells and shows potent antitumor activity in xenograft tumor models 2015 Molecular Cancer Therapeutics 14(11) 2463-2472
	Zhang, C. G., et al. Nepetaefolins A-J, Cytotoxic Chinane and Abietane Diterpenoids from Caryopteris nepetaefolia 2017 Journal of Natural Products 80(6) 1742-1749
	Zhang, P., et al. Upregulation of programmed cell death ligand 1 promotes resistance response in non-small-cell lung cancer patients treated with neo-adjuvant chemotherapy 2016 Cancer Science 107(11) 1563-1571
	Zhang, Y., et al. PP2AC Level Determines Differential Programming of p38-TSC-mTOR Signaling and Therapeutic Response to p38-Targeted Therapy in Colorectal Cancer 2015 EBioMedicine 2(12) 1944-1956
	Zhang, Y., et al. 244-MPT overcomes gefitinib resistance in non-small cell lung cancer cells 2015 Oncotarget 6(42) 44274-44288
	Zhou, X., et al. Galectin-1 is overexpressed in CD133⁺ human lung adenocarcinoma cells and promotes their growth and invasiveness 2015 Oncotarget 6(5) 3111-3122
	Zhu, H., et al. Suppressing the growth of rectal cancer xenografts derived from patient tumors by an adenovector expressing small hairpin RNA targeting Bcl-XL 2012 Journal of Gene Medicine 14(12) 761-768
	Zhu, Q, et al. Pathway activation strength is a novel independent prognostic biomarker for cetuximab sensitivity in colorectal cancer patients 2015 Human Genome Variation 2() http://dx.doi.org/10.1038/hgv.2015.9
	Hindupur, Sravanth K., et al. Identification of a novel AMPK-PEA15 axis in the anoikis-resistant growth of mammary cells. 2014 Breast cancer research: BCR 16(4) 420
	Meetze, K., et al. Neuregulin 1 expression is a predictive biomarker for response to AV-203, an ERBB3 inhibitory antibody, in human tumor models. 2015 Clinical cancer research: an official journal of the American Association for Cancer Research 21(5) 1106-14
	Shirakawa, K., et al. Inflammatory breast cancer: vasculogenic mimicry and its hemodynamics of an inflammatory breast cancer xenograft model. 2003 Breast cancer research: (3) 136-9
**Unobtainable**	Abe, J., et al. A novel vitamin D3 analog, 22-oxa-1,25-dihydroxyvitamin D3, inhibits the growth of human breast cancer in vitro and in vivo without causing hypercalcemia 1991 Endocrinology 129(2) 832-837
	Balkwill, F. R., et al. Human interferon inhibits the growth of established human breast tumours in the nude mouse 1982 International Journal of Cancer 30(2) 231-235
	Brand M., T., et al. The receptor tyrosine kinase AXL mediates nuclear translocation of the epidermal growth factor receptor 2017 Science Signaling 10(460) eaag1064 DOI: 10.1126/scisignal.aag106
	Decaudin, D. Primary human tumor xenografted models ('tumorgrafts') for good management of patients with cancer 2011 Anti-Cancer Drugs 22(9) 827-841

	Eigenmann, M. J., et al. Combining nonclinical experiments with translational PKPD modeling to differentiate erlotinib and gefitinib 2016 Molecular Cancer Therapeutics 15(12) 3110-3119
	Esmaeili, M., et al. In vivo ³¹P Magnetic Resonance Spectroscopic Imaging (MRSI) for metabolic profiling of human breast cancer xenografts 2015 Journal of Magnetic Resonance Imaging 41(3) 601-609
	Jackson, H., et al. Novel bispecific domain antibodyto LRP6 inhibits wnt and r-spondin ligand-induced wnt signaling and tumor growth 2016 Molecular Cancer Research 14(9) 859-868
	Katsiampoura, A., et al. Modeling of Patient-Derived Xenografts in Colorectal Cancer 2017 Molecular Cancer Therapeutics 16(7) 1435-1442
	Kuesters, S., et al. Correlation of ErbB2 gene status, mRNA and protein expression in a panel of >100 human tumor xenografts of different origin 2006 Onkologie 29(6) 249-256
	Lloyd, I. E., et al. X-Ray of Excised Cancerous Breast Tissue Does Not Affect Clinical Biomarker Expression 2017 Applied Immunohistochemistry and Molecular Morphology: DOI: 10.1097/PAI.0000000000000454
	Lu, J., et al. Reactivation of p53 by MDM2 inhibitor MI-77301 for the treatment of endocrine-resistant breast cancer 2016 Molecular Cancer Therapeutics 15(12) 2887-2893
	Mukohyama, J., et al. Effect of xenotransplantation site on microrna expression of human colon cancer stem cells 2016 Anticancer Research 36(7) 3679-3686
	Pereira, C., et al. Genomic profiling of patient-derived xenografts for lung cancer identifies B2M inactivation impairing immunorecognition 2017 Clinical Cancer Research 23(12) 3203-3213
	Raimbourg, J., et al. Sensitization of EGFR wild-type non-small cell lung cancer cells to EGFR-tyrosine kinase inhibitor erlotinib 2017 Molecular Cancer Therapeutics 16(8) 1634-1644
	Sargent, J. M., et al. Ex vivo effects of the dual topoisomerase inhibitor tafluposide (F 11782) on cells isolated from fresh tumor samples taken from patients with cancer 2003 Anti-Cancer Drugs 14(6) 467-473
	Wei, X., et al. PSCA and MUC1 in non-small-cell lung cancer as targets of chimeric antigen receptor T cells 2017 OncoImmunology 6(3) DOI: 10.1080/2162402X.2017.1284722
	Weiswald, L. B., et al. Inactivation of the kinase domain of CDK10 prevents tumor growth in a preclinical model of colorectal cancer, and is accompanied by downregulation of Bcl-2 2017 Molecular Cancer Therapeutics 29: doi: 10.1158/1535-7163.MCT-16-0666
	Wiedemann, G., et al. Hyperthermia enhances mitoxantrone cytotoxicity on human breast carcinoma and sarcoma xenografts in nude mice 1992 International Journal of Radiation Oncology Biology Physics 24(4) 669-673
	Zhang, J. S., et al. Experimental radiotherapy and metastasis of human lung cancer xenografts in nude mice 1994 Chinese medical journal 107(7) 491-495
	Balkwill, F R., et al. Effects of tumour necrosis factor on human tumour xenografts in nude mice. 1987 Ciba Foundation symposium 131: 154-69
