# Supplementary material for: A systematic review of the validity of patient derived xenograft (PDX) models: the implications for translational research and personalised medicine
Source: PeerJ. 2018 Nov 21;6:e5981. doi: 10.7717/peerj.5981 (PMC6252062; doi:10.7717/peerj.5981)
Supplement: Table S5 [file peerj-06-5981-s005.pdf]

Supplemental Table S5. List of included studies and records.

A. Breast

Country	Primary Laboratory location	PDX ID	First Author Surname and Year	Associated refs	ARRIVE guidelines?
Canada	BC Cancer Agency	SA-xxx	Eirew 2015*(1)*		no
France	INSERM	CRCM-xxx	Charafe-Jauffret 2013*(2)*		no
	Institute Curie, Paris	HBCx-3, 21, 22, 29, 34, 36	Cottu 2012*(3)*	Marangoni 2007*(4)*; Reyal 2012*(5)*	yes
	University of Montpellier	B-xxxx	du Manoir 2014*(6)*		no
Germany	Max-Delbruick-Centrum fur Molekulare Medizin, Berlin	3366	Naundorf 1992*(7)*		no
Italy	Istituto Europeo di Oncologia, Milan	T1 to T4; TA to TD.	Tosoni 2017*(8)*		no
Japan	National Cancer Center Research Institute, Tokyo	WIBC-9	Shirakawa 2001*(9)*		no
		tMK-x, tHK-x, tYK-x, tTY-x	Wakasugi 1995*(10)*		no
Korea	Seoul National University College of Medicine	NR	Moon 2015*(11)*		no
Norway	Oslo University Hospital	MAS98.06, MAS98.12	Bergamaschi 2009*(12)*		no
Spain	Vall d'Hebron Institute of Oncology, Barcelona	PDX-xxx	Morancho 2016*(13)*		no
UK	University of Cambridge	AB-xxx; HCI-xxx; IC006; IC007; STG-xxx; VHIO-xxx	Bruna 2016*(14)*		no
	Manchester Cancer Research Centre	BBxRCxxx	Eyre 2016*(15)*		no
USA	Howard Hughes Medical Institute, University of Michigan	T1 to T9	Al-Hajj 2003*(16)*		no
	University of Iowa	IOWA-1T	Bogachek 2015*(17)*		no
	Pfizer, Stemcentrx	BR-xx	Damelin 2015*(18)*		no
	University of Utah Health Sciences Center	HCI001-004.	DeRose 2011*(19)*	Bruna 2016*(14)*	no
	University of Colorado Denver Anschutz	PT 12, 15, 16, 18	Kabos 2012*(20)*		no
	Department of Cancer Biology, Duarte	COH-xxxx	Kanaya 2017*(21)*		no
	Washington University in St. Louis	WHIM-xx	Li 2013*(22)*	Huang 2017*(23)*	no
	The University of Texas MD Anderson Cancer Center, Houston	BCX-xxx	McAuliffe 2015*(24)*		no
		BCM-xxxx.	Zhang 2013*(25)*		no
	Albert Einstein College of Medicine, NY	HT17, HT39	Patsialou 2012*(26)*		no
	The University of Kansas Medical Center	NR	Valdez 2011*(27)*		no
	The Wistar Institute of Anatomy and Biology	HUP-1 to 3; CHTN-1 to 8; MHBC-1 to 4	Visonneau 1998*(28)*		no
	Stanford University School of Medicine	SUTI-097; 103; 110; 151; 319; 368.	Zhang 2014*(29)*		no

1. P. Eirew, A. Steif, J. Khattra, G. Ha, D. Yap, H. Farahani, K. Gelmon, S. Chia, C. Mar, A. Wan, E. Laks, J. Biele, K. Shumansky, J. Rosner, A. McPherson, C. Nielsen, A. J. L. Roth, C. Lefebvre, A. Bashashati, C. de Souza, C. Siu, R. Aniba, J. Brimhall, A. Oloumi, T. Osako, A. Bruna, J. L. Sandoval, T. Algara, W. Greenwood, K. Leung, H. Cheng, H. Xue, Y. Wang, D. Lin, A. J. Mungall, R. Moore, Y. Zhao, J. Lorette, L. Nguyen, D. Huntsman, C. J. Eaves, C. Hansen, M. A. Marra, C. Caldas, S. P. Shah, S. Aparicio, Dynamics of genomic clones in breast cancer patient xenografts at single-cell resolution, *Nature* **518**, 422–426 (2014).

2. E. Charafe-Jauffret, C. Ginestier, F. Bertucci, O. Cabaud, J. Wicinski, P. Finetti, E. Josselin, J. Adelaide, T. T. Nguyen, F. Monville, J. Jacquemier, J. Thomassin-Piana, G. Pinna, A. Jalaguier, E. Lambaudie, G. Houvenaeghel, L. Xerri, A. Harel-Bellan, M. Chaffanet, P. Viens, D. Birnbaum, ALDH1-positive cancer stem cells predict engraftment of primary breast tumors and are governed by a common stem cell program, *Cancer Research* **73**, 7290–7300 (2013).

3. P. Cottu, E. Marangoni, F. Assayag, P. De Cremoux, A. Vincent-Salomon, C. Guyader, L. De Plater, C. Elbaz, N. Karboul, J. J. Fontaine, S. Chateau-Joubert, P. Boudou-Rouquette, S. Alran, V. Dangles-Marie, D. Gentien, M. F. Poupon, D. Decaudin, Modeling of response to endocrine therapy in a panel of human luminal breast cancer xenografts, *Breast Cancer Research and Treatment* **133**, 595–606 (2012).

4. E. Marangoni, A. Vincent-Salomon, N. Auger, A. Degeorges, F. Assayag, P. De Cremoux, L. De Plater, C. Guyader, G. De Pinieux, J. G. Judde, M. Rebucci, C. Tran-Perennou, X. Sastre-Garau, B. Sigal-Zafrani, O. Delattre, V. Diéras, M. F. Poupon, A new model of patient tumor-derived breast cancer xenografts for preclinical assays, *Clinical Cancer Research* **13**, 3989–3998 (2007).

5. F. Reyal, C. Guyader, C. Decraene, C. Lucchesi, N. Auger, F. Assayag, L. De Plater, D. Gentien, M.-F. Poupon, P. Cottu, P. De Cremoux, P. Gestraud, A. Vincent-Salomon, J.-J. Fontaine, S. Roman-Roman, O. Delattre, D. Decaudin, E. Marangoni, Molecular profiling of patient-derived breast cancer xenografts., *Breast cancer research : BCR* **14**, R11 (2012).

6. S. du Manoir, B. Orsetti, R. Bras-Gonçalves, T. T. Nguyen, L. Lasorsa, F. Boissière, B. Massemin, P. E. Colombo, F. Bibeau, W. Jacot, C. Theillet, Breast tumor PDXs are genetically plastic and correspond to a subset of aggressive cancers prone to relapse, *Molecular Oncology* **8**, 431–443 (2014).

7. H. Naundorf, I. Fichtner, B. Biittner, J. Frege, Establishment and characterization of a new human oestradiol-and progesterone-receptor-positive mammary carcinoma serially transplantable in nude mice *, *J Cancer Res Clin Oncol* **119**, 35–40 (1992).

8. D. Tosoni, S. Pambianco, B. Ekalle Soppo, S. Zecchini, G. Bertalot, G. Pruneri, G. Viale, P. P. Di Fiore, S. Pece, Pre-clinical validation of a selective anti-cancer stem cell therapy for Numb-deficient human breast cancers, *EMBO Molecular Medicine* **9**, 655–671 (2017).

9. K. Shirakawa, H. Tsuda, Y. Heike, K. Kato, R. Asada, M. Inomata, H. Sasaki, F. Kasumi, M. Yoshimoto, T. Iwanaga, F. Konishi, M. Terada, H. Wakasugi, Absence of endothelial cells, central necrosis, and fibrosis are associated with aggressive inflammatory breast cancer, *Cancer Research* **61**, 445–451 (2001).

10. H. Wakasugi, K. Koyama, M. Gyotoku, M. Yoshimoto, S. Hirohashi, T. Sugimura, M. Terada, Frequent Development of Murine T-Cell Lymphomas with TcRα/β+, CD4-/8 Phenotype after Implantation of Human Inflammatory Breast Cancer Cells in BALB/c Nude Mice*Japanese Journal of Cancer Research* **86**, 1086–1096 (1995).

11. H. G. Moon, K. Oh, J. Lee, M. Lee, J. Y. Kim, T. K. Yoo, M. W. Seo, A. K. Park, H. S. Ryu, E. J. Jung, N. Kim, S. Jeong, W. Han, D. S. Lee, D. Y. Noh, Prognostic and functional importance of the engraftment-associated genes in the patient-derived xenograft models of triple-negative breast cancers, *Breast Cancer Research and Treatment* **154**, 13–22 (2015).

12. A. Bergamaschi, G. O. Hjortland, T. Triulzi, T. Sørlie, H. Johnsen, A. H. Ree, H. G. Russnes, S. Tronnes, G. M. Mælandsmo, O. Fodstad, A. L. Borresen-Dale, O. Engebraaten, Molecular profiling and characterization of luminal-like and basal-like in vivo breast cancer xenograft models, *Molecular Oncology* **3**, 469–482 (2009).

13. B. Morancho, M. Zacarias-Fluck, A. Esgueva, C. Bernado-Morales, S. Di Cosimo, A. Prat, J. Cortes, J. Arribas, I. T. Rubio, Modeling anti-IL-6 therapy using breast cancer patient-derived xenografts, *Oncotarget* **7**, 67956–67965 (2016).

14. A. Bruna, O. M. Rueda, W. Greenwood, A. S. Batra, M. Callari, R. N. Batra, K. Pogrebniak, J. Sandoval, J. W. Cassidy, A. Tufegdzic-Vidakovic, S. J. Sammut, L. Jones, E. Provenzano, R. Baird, P. Eirew, J. Hadfield, M. Eldridge, A. McLaren-Douglas, A. Barthorpe, H. Lightfoot, M. J. O'Connor, J. Gray, J. Cortes, J. Baselga, E. Marangoni, A. L. Welm, S. Aparicio, V. Serra, M. J. Garnett, C. Caldas, A Biobank of Breast Cancer Explants with Preserved Intra-tumor Heterogeneity to Screen Anticancer Compounds, *Cell* **167**, 260–274.e22 (2016).

15. R. Eyre, D. G. Alférez, K. Spence, M. Kamal, F. L. Shaw, B. M. Simões, A. Santiago-Gómez, A. Sarmiento-Castro, M. Bramley, M. Absar, Z. Saad, S. Chatterjee, C. Kirwan, A. Gandhi, A. C. Armstrong, A. M. Wardley, C. S. O'Brien, G. Farnie, S. J. Howell, R. B. Clarke, Patient-derived Mammosphere and Xenograft Tumour Initiation Correlates with Progression to Metastasis, *Journal of Mammary Gland Biology and Neoplasia* **21**, 99–109 (2016).

16. M. Al-Hajj, M. S. Wicha, A. Benito-Hernandez, S. J. Morrison, M. F. Clarke, Prospective identification of tumorigenic breast cancer cells, *Proceedings of the National Academy of Sciences* **100**, 3983–3988 (2003).

17. M. V. Bogachek, J. M. Park, J. P. De Andrade, M. V. Kulak, J. R. White, T. Wu, P. M. Spanheimer, T. B. Bair, A. K. Olivier, R. J. Weigel, A Novel Animal Model for Locally Advanced Breast Cancer, *Annals of Surgical Oncology* **22**, 866–873 (2015).

18. M. Damelin, A. Bankovich, A. Park, J. Aguilar, W. Anderson, M. Santaguida, M. Aujay, S. Fong, K. Khandke, V. Pulito, E. Ernstoff, P. Escarpe, J. Bernstein, M. Pysz, W. Zhong, E. Upeslacis, J. Lucas, J. Lucas, T. Nichols, K. Loving, O. Foord, J. Hampl, R. Stull, F. Barletta, H. Falahatpisheh, P. Sapra, H. P. Gerber, S. J. Dylla, Anti-EFNA4 calicheamicin conjugates effectively target triple-negative breast and ovarian tumor-initiating cells to result in sustained tumor regressions, *Clinical Cancer Research* **21**, 4165–4173 (2015).

19. Y. S. DeRose, G. Wang, Y. Lin, P. S. Bernard, S. S. Buys, M. T. W. Ebbert, R. Factor, C. Matsen, B. A. Milash, E. Nelson, L. Neumayer, R. L. Randall, I. J. Stijleman, B. E. Welm, A. L. Welm, Tumor grafts derived from women with breast cancer authentically reflect tumor pathology, growth, metastasis and disease outcomes, *Nature Medicine* **17**, 1514–1520 (2011).

20. P. Kabos, J. Finlay-Schultz, C. Li, E. Kline, C. Finlayson, J. Wisell, C. A. Manuel, S. M. Edgerton, J. C. Harrell, A. Elias, C. A. Sartorius, Patient-derived luminal breast cancer xenografts retain hormone receptor heterogeneity and help define unique estrogen-dependent gene signatures, *Breast Cancer Research and Treatment* **135**, 415–432 (2012).

21. N. Kanaya, G. Somlo, J. Wu, P. Frankel, M. Kai, X. Liu, S. V. Wu, D. Nguyen, N. Chan, M. Y. Hsieh, M. Kirschenbaum, L. Kruper, C. Vito, B. Badie, J. H. Yim, Y. Yuan, A. Hurria, C. Peiguo, J. Mortimer, S. Chen, Characterization of patient-derived tumor xenografts (PDXs) as models for estrogen receptor positive (ER+HER2− and ER+HER2+) breast cancers, *Journal of Steroid Biochemistry and Molecular Biology* **170**, 65–74 (2017).

22. S. Li, D. Shen, J. Shao, R. Crowder, W. Liu, A. Prat, X. He, S. Liu, J. Hoog, C. Lu, L. Ding, O. L. Griffith, C. Miller, D. Larson, R. S. Fulton, M. Harrison, T. Mooney, J. F. McMichael, J. Luo, Y. Tao, R. Goncalves, C. Schlosberg, J. F. Hiken, L. Saied, C. Sanchez, T. Giuntoli, C. Bumb, C. Cooper, R. T. Kitchens, A. Lin, C. Phommaly, S. R. Davies, J. Zhang, M. S. Kavuri, D. McEachern, Y. Y. Dong, C. Ma, T. Pluard, M. Naughton, R. Bose, R. Suresh, R. McDowell, L. Michel, R. Aft, W. Gillanders, K. DeSchryver, R. K. Wilson, S. Wang, G. B. Mills, A. Gonzalez-Angulo, J. R. Edwards, C. Maher, C. M. Perou, E. R. Mardis, M. J. Ellis, Endocrine-Therapy-Resistant ESR1 Variants Revealed by Genomic Characterization of Breast-Cancer-Derived Xenografts, *Cell Reports* **4**, 1116–1130 (2013).

23. K. L. Huang, S. Li, P. Mertins, S. Cao, H. P. Gunawardena, K. V. Ruggles, D. R. Mani, K. R. Clauser, M. Tanioka, J. Usary, S. M. Kavuri, L. Xie, C. Yoon, J. W. Qiao, J. Wrobel, M. A. Wyczalkowski, P. Erdmann-Gilmore, J. E. Snider, J. Hoog, P. Singh, B. Niu, Z. Guo, S. Q. Sun, S. Sanati, E. Kawaler, X. Wang, A. Scott, K. Ye, M. D. McLellan, M. C. Wendl, A. Malovannaya, J. M. Held, M. A. Gillette, D. Fenyö, C. R. Kinsinger, M. Mesri, H. Rodriguez, S. R. Davies, C. M. Perou, C. Ma, R. Reid Townsend, X. Chen, S. A. Carr, M. J. Ellis, L. Ding, Proteogenomic integration reveals therapeutic targets in breast cancer xenografts, *Nature Communications* **8** (2017), doi:10.1038/ncomms14864.

24. P. F. McAuliffe, K. W. Evans, A. Akcakanat, K. Chen, X. Zheng, H. Zhao, A. K. Eterovic, T. Sangai, A. M. Holder, C. Sharma, H. Chen, K. A. Do, E. Tarco, M. Gagea, K. A. Naff,

A. Sahin, A. S. Multani, D. M. Black, E. A. Mittendorf, I. Bedrosian, G. B. Mills, A. M. Gonzalez-Angulo, F. Meric-Bernstam, Ability to generate patient-derived Breast cancer xenografts is enhanced in chemoresistant disease and predicts poor patient outcomes, *PLoS ONE* **10**, 1–20 (2015).

25. X. Zhang, S. Claerhout, A. Prat, L. E. Dobrolecki, I. Petrovic, Q. Lai, M. D. Landis, L. Wiechmann, R. Schiff, M. Giuliano, H. Wong, S. W. Fuqua, A. Contreras, C. Gutierrez, J. Huang, S. Mao, A. C. Pavlick, A. M. Froehlich, M. F. Wu, A. Tsimelzon, S. G. Hilsenbeck, E. S. Chen, P. Zuloaga, C. A. Shaw, M. F. Rimawi, C. M. Perou, G. B. Mills, J. C. Chang, M. T. Lewis, A renewable tissue resource of phenotypically stable, biologically and ethnically diverse, patient-derived human breast cancer xenograft models, *Cancer Research* **73**, 4885–4897 (2013).

26. A. Patsialou, Y. Wang, J. Lin, K. Whitney, S. Goswami, P. a Kenny, J. S. Condeelis, Selective gene-expression profiling of migratory tumor cells in vivo predicts clinical outcome in breast cancer patients., *Breast cancer research : BCR* **14**, R139 (2012).

27. K. E. Valdez, F. Fan, W. Smith, D. C. Allred, D. Medina, F. Behbod, Human primary ductal carcinoma in situ (DCIS) subtype-specific pathology is preserved in a mouse intraductal (MIND) xenograft model, *The Journal of Pathology* **225**, 565–573 (2011).

28. S. Visonneau, A. Cesano, M. H. Torosian, E. J. Miller, D. Santoli, Growth Characteristics and Metastatic Properties of Human Breast Cancer Xenografts in Immunodeficient Mice, *American Journal of Pathology* **152**, 1299–1311 (1998).

29. H. Zhang, A. L. Cohen, S. Krishnakumar, I. L. Wapnir, S. Veeriah, G. Deng, M. A. Coram, C. M. Piskun, T. A. Longacre, M. Herrler, D. O. Frimannsson, M. L. Telli, F. M. Dirbas, A. C. Matin, S. H. Dairkee, B. Larijani, G. V. Glinsky, A. H. Bild, S. S. Jeffrey, Patient-derived xenografts of triple-negative breast cancer reproduce molecular features of patient tumors and respond to mTOR inhibition, *Breast Cancer Research* **16**, 1–16 (2014).

B. Colon

Country	Primary Laboratory location	PDX ID	First Author Surname and Year	Associated refs	ARRIVE guidelines?
China	Key laboratory of Carcinogenesis and Translational Research, Department of Surgery, Peking University Cancer Hospital and Institute	BCCxxxx	Zhang 2015*(30)*		no
	Department of Colorectal Surgery, Zhejiang University, Hangzhou	16670x 18657x, 21835x	Zhou 2011*(31)*		no
	Department of Surgical Oncology, Zhejiang University	NR	Guan 2016*(32)*		no
			Jin 2011*(33)*		no
France	Laboratoire de Physiopathologie Hépatique, Centre National de la Recherche Scientifique, Université René Descartes-Paris	CT320, CT329 + others	Dangles-Marie 2007*(34)*		no
	Oncodesign, Dijon	CR-IC-xxxx, CR-IGR-xxxx, CR-LRB-xxxx	Julien 2012*(35)*		no
Germany	Department of General, Vascular, Thoracic and Transplantation Surgery, University of Rostock	HROC107, 108, 119, 122, 123, 125, 129, 130, 131, 135	Gock 2016*(36)*		no
	Department of General Surgery, Division of Molecular Oncology and Immunotherapy, Clinic for Surgery, Rostock	HROC 24 to 89; 183	Linnebacher 2010*(37)*	Kuehn 2016*(38)*; Maletzki 2015*(39)*	no
Japan	Division of Molecular and Cellular Biology, Kobe University Graduate School of Medicine	NR	Mukohyama 2016*(40)*		no
Korea	Department of Hemato-Oncology, Gil Medical Center, Gachon University	Numerical	Lee 2014*(41)*		no
	Department of Surgery, Samsung Medical Center, Sungkyunkwan University School of Medicine, Seoul	NR	Oh 2015*(42)*		no
Singapore	Institute of Bioengineering and Nanotechnology Singapore	FIT-CRC-086, FIT-CRC-104	Mohamed Suhaimi 2017*(43)*		no
Spain	Institute for Research in Biomedicine (IRB Barcelona)	CCR-038, CCR-010	Gupta 2015*(44)*		no
	Translational Program, Stem Cells and Cancer Laboratory; Passeig Vall d'Hebron University, Barcelona	NR	Puig 2013*(45)*		no
	Vall d'Hebrón Institut d'Oncología (VHIO), Stem Cell and Cancer Laboratory, Barcelona	NR	Tenbaum 2012*(46)*		no
UK	Department of Surgery, St George's Hospital Medical School, London	NR	Davies 1981*(47)*		no
USA	Department of Immunology, Roswell Park Cancer Institute, NY	NR	Naka 2002*(48)*		no
	Department of Surgery, University of Texas Medical Branch	NR	Chao 2017*(49)*		no

	Program in Immunology, Clinical Research Division, Fred Hutchinson Cancer Research Center, Seattle	D55949, D60789, D61211, D61540, WD2112, WD2713, P2822, P2827, P2842	Chou 2013*(50)*		no
	Division of Medical Oncology, Duke University, North Carolina	CRC105, CRC093, CRC066, CRC030, CRC028	Uronis 2012*(51)*		no
	Laboratory of Systems Biology, Van Andel Institute, MI	CRC02, CRC06, CRC09, CDC10, CRC12, CRC14, CRC17, CRC18, CRC19, REC02, REC09, REC12, REC16, REC19	Burgenske 2014*(52)*		no
	Department of Medicine, Division of Medical Oncology, University of Colorado Cancer Center	CRC001, 006, 007, 010, 012, 020, 021, 026, 027, 034, 035, 036, 040, 042, 052, 065.	Arcaroli 2013*(53)*		no
	Oncology Discovery Research, Eli Lilly and Company, Indianapolis	CTGxxxx	Yao 2017*(54)*		no
	Translational Oncology Program, University of Michigan Medical School, Ann Arbor, Michigan	UM-CRC-xx-xxxx	Ziemke 2015*(55)*		no
	Department of Anesthesiology, University of Texas Medical Branch	NR	Szabo 2013*(56)*		no
	The Jackson Laboratory for Genomic Medicine, CT	NR	Cho 2017*(57)*		no
	Crown Bioscience, Inc., CA	CRxxxx	Chen 2015*(58)*		no

30. L. Zhang, Y. Liu, X. Wang, Z. Tang, S. Li, Y. Hu, X. Zong, X. Wu, Z. Bu, A. Wu, Z. Li, Z. Li, X. Huang, L. Jia, Q. Kang, Y. Liu, D. Sutton, L. Wang, L. Luo, J. Ji, The extent of inflammatory infiltration in primary cancer tissues is associated with lymphomagenesis in immunodeficient mice, *Scientific Reports* **5**, 1–6 (2015).

31. W. Zhou, H. Zhu, W. Chen, X. Hu, X. Pang, J. Zhang, X. Huang, B. Fang, C. He, Treatment of patient tumor-derived colon cancer xenografts by a TRAIL gene-armed oncolytic adenovirus, *Cancer Gene Therapy* **18**, 336–345 (2011).

32. Z. Guan, X. Chen, X. Jiang, Z. Li, X. Yu, K. Jin, J. Cao, L. Teng, Establishing a patient-derived colorectal cancer xenograft model for translational research, *Int J Clin Exp Med* **9**, 21346–21357 (2016).

33. K. Jin, G. Li, B. Cui, J. Zhang, H. Lan, N. Han, B. Xie, F. Cao, K. He, H. Wang, Z. Xu, L. Teng, T. Zhu, Assessment of a novel vegf targeted agent using patient-derived tumor tissue xenograft models of colon carcinoma with lymphatic and hepatic metastases, *PLoS ONE* **6** (2011), doi:10.1371/journal.pone.0028384.

34. V. Dangles-Marie, M. Pocard, S. Richon, L. B. Weiswald, F. Assayag, P. Saulnier, J. G. Judde, J. L. Janneau, N. Auger, P. Validire, B. Dutrillaux, F. Praz, D. Bellet, M. F. Poupon, Establishment of human colon cancer cell lines from fresh tumors versus xenografts: Comparison of success rate and cell line features, *Cancer Research* **67**, 398–407 (2007).

35. S. Julien, A. Merino-Trigo, L. Lacroix, M. Pocard, D. Goeŕé, P. Mariani, S. Landron, L. Bigot, F. Nemati, P. Dartigues, L. B. Weiswald, D. Lantuas, L. Morgand, E. Pham, P. Gonin, V. Dangles-Marie, B. Job, P. Dessen, A. Bruno, A. Pierré, H. De Thé, H. Soliman, M. Nunes, G. Lardier, L. Calvet, B. Demers, G. Prévost, P. Vrignaud, S. Roman-Roman, O. Duchamp, C. Berthet, Characterization of a large panel of patient-derived tumor xenografts representing the clinical heterogeneity of human colorectal cancer, *Clinical*

Cancer Research **18**, 5314–5328 (2012).

36. M. Gock, F. Kühn, C. S. Mullins, M. Krohn, F. Prall, E. Klar, M. Linnebacher, Tumor Take Rate Optimization for Colorectal Carcinoma Patient-Derived Xenograft Models, *BioMed Research International* **2016** (2016), doi:10.1155/2016/1715053.

37. M. Linnebacher, C. Maletzki, C. Ostwald, U. Klier, M. Krohn, E. Klar, F. Prall, Cryopreservation of human colorectal carcinomas prior to xenografting, *BMC Cancer* **10**, 362 (2010).

38. F. Kuehn, C. S. Mullins, M. Krohn, C. Harnack, R. Ramer, O. H. Krämer, E. Klar, M. Huehns, M. Linnebacher, Establishment and characterization of HROC69 - A Crohn's related colonic carcinoma cell line and its matched patient-derived xenograft, *Scientific Reports* **6**, 1–10 (2016).

39. C. Maletzki, M. Huehns, P. Knapp, N. Waukosin, E. Klar, F. Prall, M. Linnebacher, Functional characterization and drug response of freshly established patient-derived tumor models with cpg island methylator phenotype, *PLoS ONE* **10**, 1–14 (2015).

40. J. Mukohyama, D. Iwakiri, Y. Zen, T. Mukohara, H. Minami, Y. Kakeji, Y. Shimono, Evaluation of the risk of lymphomagenesis in xenografts by the PCR-based detection of EBV BamHI W region in patient cancer specimens., *Oncotarget* **7** (2016), doi:10.18632/oncotarget.10322.

41. W.-S. Lee, H.-Y. Kim, J. Y. Seok, H. H. Jang, Y. H. Park, S.-Y. Kim, D. B. Shin, S. Hong, Genomic Profiling of Patient-Derived Colon Cancer Xenograft Models, *Medicine* **93**, e298 (2014).

42. B. Y. Oh, W. Y. Lee, S. Jung, H. K. Hong, D.-H. Nam, Y. A. Park, J. W. Huh, S. H. Yun, H. C. Kim, H.-K. Chun, Y. B. Cho, Correlation between tumor engraftment in patient-derived xenograft models and clinical outcomes in colorectal cancer patients., *Oncotarget* **6**, 16059–68 (2015).

43. N.-A. Mohamed Suhaimi, W. M. Phyo, H. Y. Yap, S. H. Y. Choy, X. Wei, Y. Choudhury, W. J. Tan, L. A. P. Y. Tan, R. S. Y. Foo, S. H. S. Tan, Z. Tiang, C. F. Wong, P. K. Koh, M.-H. Tan, Metformin Inhibits Cellular Proliferation and Bioenergetics in Colorectal Cancer Patient-Derived Xenografts., *Molecular cancer therapeutics* **16**, 2035–2044 (2017).

44. J. Gupta, A. Igea, M. Papaioannou, P. P. Lopez-Casas, E. Llonch, M. Hidalgo, V. G. Gorgoulis, A. R. Nebreda, Pharmacological inhibition of p38 MAPK reduces tumor growth in patient-derived xenografts from colon tumors, *Oncotarget* **6**, 8539–8551 (2015).

45. I. Puig, I. Chicote, S. P. Tenbaum, O. Arqués, J. R. Herance, J. D. Gispert, J. Jimenez, S. Landolfi, K. Caci, H. Allende, L. Mendizabal, D. Moreno, R. Charco, E. Espín, A. Prat, M. E. Elez, G. Argilés, A. Vivancos, J. Tabernero, S. Rojas, H. G. Palmer, A personalized preclinical model to evaluate the metastatic potential of patient-derived colon cancer initiating cells, *Clinical Cancer Research* **19**, 6787–6801 (2013).

46. S. P. Tenbaum, P. Ordóñez-Morán, I. Puig, I. Chicote, O. Arqués, S. Landolfi, Y. Fernández, J. R. Herance, J. D. Gispert, L. Mendizabal, S. Aguilar, S. Ramón Y Cajal, S. Schwartz, A. Vivancos, E. Espín, S. Rojas, J. Baselga, J. Tabernero, A. Muñoz, H. G. Palmer, β-Catenin confers resistance to PI3K and AKT inhibitors and subverts FOXO3a to promote metastasis in colon cancer, *Nature Medicine* **18**, 892–901 (2012).

47. G. Davies, D. Duke, A. G. Grant, S. A. Kelly, J. Hermon-Taylor, Growth of human digestive-tumour xenografts in athymic nude rats, *British Journal of Cancer* **43**, 53–58 (1981).

48. T. Naka, K. Sugamura, B. L. Hylander, M. B. Widmer, Y. M. Rustum, E. A. Repasky, Effects of tumor necrosis factor-related apoptosis-inducing ligand alone and in combination with chemotherapeutic agents on patients' colon tumors grown in SCID mice, *Cancer Res* **62**, 5800–5806 (2002).

49. C. Chao, S. G. Widen, T. G. Wood, J. R. Zatarain, P. Johnson, A. Gajjar, G. Gomez, S. Qiu, J. Thompson, H. Spratt, M. R. Hellmich, Patient-derived Xenografts from Colorectal Carcinoma: A Temporal and Hierarchical Study of Murine Stromal Cell Replacement, *Anticancer Research* **37**, 3405–3412 (2017).

50. J. Chou, M. P. Fitzgibbon, C. L. L. Mortales, A. M. H. Towlerton, M. P. Upton, R. S. Yeung, M. W. McIntosh, E. H. Warren, Phenotypic and transcriptional fidelity of patient-

Derived colon cancer xenografts in immune-deficient mice, *PLoS ONE* **8** (2013), doi:10.1371/journal.pone.0079874.

51. J. M. Uronis, T. Osada, S. McCall, X. Y. Yang, C. Mantyh, M. A. Morse, H. K. Lyerly, B. M. Clary, D. S. Hsu, Histological and molecular evaluation of patient-derived colorectal cancer explants, *PLoS ONE* **7** (2012), doi:10.1371/journal.pone.0038422.

52. D. M. Burgenske, D. J. Monsma, D. Dylewski, S. B. Scott, A. D. Sayfie, D. G. Kim, M. Luchtefeld, K. R. Martin, P. Stephenson, G. Hostetter, N. Dujovny, J. P. MacKeigan, Establishment of genetically diverse patient-derived xenografts of colorectal cancer., *American journal of cancer research* **4**, 824–37 (2014).

53. J. J. Arcaroli, K. S. Quackenbush, A. Purkey, R. W. Powell, T. M. Pitts, S. Bagby, A. C. Tan, B. Cross, K. McPhillips, E. K. Song, W. M. Tai, R. A. Winn, K. Bikkavilli, M. Vanscoyk, S. G. Eckhardt, W. A. Messersmith, Tumours with elevated levels of the Notch and Wnt pathways exhibit efficacy to PF-03084014, a γ-secretase inhibitor, in a preclinical colorectal explant model, *British Journal of Cancer* **109**, 667–675 (2013).

54. Y. M. M. Yao, G. P. Donoho, P. W. Iversen, Y. Zhang, R. D. Van Horn, A. Forest, R. D. Novosiadly, Y. W. Webster, P. Ebert, S. Bray, J. C. Ting, A. Aggarwal, J. R. Henry, R. V. Tiu, G. D. Plowman, S. Bin Peng, Mouse PDX trial suggests synergy of concurrent inhibition of RAF and EGFR in colorectal cancer with BRAF or KRAS mutations, *Clinical Cancer Research* **23**, 5547–5560 (2017).

55. E. K. Ziemke, J. S. Dosch, J. D. Maust, A. Shettigar, A. Sen, T. H. Welling, K. M. Hardiman, J. S. Sebolt-Leopold, Sensitivity of KRAS-Mutant colorectal cancers to combination therapy that cotargets MEK and CDK4/6, *Clinical Cancer Research* **22**, 405–414 (2015).

56. C. Szabo, C. Coletta, C. Chao, K. Modis, B. Szczesny, A. Papapetropoulos, M. R. Hellmich, Tumor-derived hydrogen sulfide, produced by cystathionine- -synthase, stimulates bioenergetics, cell proliferation, and angiogenesis in colon cancer, *Proceedings of the National Academy of Sciences* **110**, 12474–12479 (2013).

57. S.-Y. Cho, J. Y. Han, D. Na, W. Kang, A. Lee, J. Kim, J. Lee, S. Min, J. Kang, J. Chae, J.-I. Kim, H. Park, W.-S. Lee, C. Lee, A Novel Combination Treatment Targeting BCL-X$_{L}$ and MCL1 for *KRAS/BRAF* -mutated and *BCL2L1* -amplified Colorectal Cancers, *Molecular Cancer Therapeutics* **16**, 2178–2190 (2017).

58. D. Chen, X. Huang, J.-P. Wery, Q.-X. Li, J. Cai, S. Guo, W. Qian, J.-P. Wery, Q.-X. Li, A set of defined oncogenic mutation alleles seems to better predict the response to cetuximab in CRC patient-derived xenograft than KRAS 12/13 mutations, *Oncotarget* **6**, 40815–40821 (2015).

C. Lung

Country	Laboratory location	PDX ID	First Author Surname and Year	Associated refs	ARRIVE guidelines?
Australia	Monash University, Clayton, Victoria	**LX -101 to 112**	Leong 2014*(59)*		no
Australia; Canada	Ludwig Institute for Cancer Research., Melbourne and Ontario Cancer Institute, Toronto	**NR**	John 2011*(60)*	John 2012*(61)*	no
Australia; USA	Monash Institute of Medical Research and John Hopkins University, Baltimore	**LX22, LX33, LX36**	Hann 2008*(62)*	Daniel 2009*(63)*	no
Canada	Dept. Cancer Endocrinology, Vancouver General Hospital	**LU-1 to 18**	Cutz 2006*(64)*		no
	Dept. Cancer Endocrinology, BC Cancer Agency, University of British Columbia; and The Living Tumor Laboratory, Vancouver Prostate Centre	**NR**	Dong 2010*(65)*		no
	Princess Margaret Cancer Centre, University Health Network, University of Toronto	**PHLC 137, 148,164,192**	Stewart 2015*(66)*		no
China	Discovery Services, Shanghai	**LU-01-xxxx**	Fang 2014*(67)*		no
	Dept. Thoracic Surgery, Peking University Cancer Hospital and Institute	**NR**	Ma 2016*(68)*		no
	Medical Research Center of Guangdong General Hospital & Guangdong Academy of Medical Sciences, Guangdong Lung Cancer Institute	**L004-L140**	Zhang 2013*(25)*		no
	Dept. Radiology, Jiangsu Cancer Institute and Hospital, Nanjing, Jiangsu	**NR**	Zhuang 2017*(69)*		no
China; USA	Crown Bioscience, CA, USA and China	**LU-0858, 1868, 1235, 1901, 2503 (HuPrime)**	Yang 2013*(70)*		no
France	Laboratory of Clinical and Experimental Pathology, Louis Pasteur Hospital, Nice	**LUN-NIC-XXXX**	Llie 2015*(71)*		no
Germany	Max Delbruck Center for Molecular Medicine, Berlin	**PLCxxxx, SQCxxxx, LCCxxxx, ADCxxxx**	Fichtner 2008*(72)*	Merk 2009*(73)*	no
Italy	Division of Pathology, Fondazione IRCCS Ca' Granda Ospedale Maggiore Policlinico, Milan	**NR**	Russo 2015*(74)*		no
	Fondazione Istituto di Ricovero e Cura a Carattere Scientifico, Istituto Nazionale dei Tumori, Milan	**NR**	Bertolini 2009*(75)*		no
South Korea	Samsung Genome Institute, Samsung Medical Center, Seoul	**NR**	Kim 2015*(76)*		no
	Asan Institute for Life Science, University of Ulsan College of Medicine, Seoul	**NR**	Seol 2013*(77)*		no
Spain	Experimental Oncology, Centro Nacional de Investigaciones Oncológicas (CNIO), Madrid	**PDX 1, 2, 3**	Ambrogio 2016*(78)*		no
USA	Virginia Tech Carilion School of Medicine, Virginia	**LU-064, 073, 086, 095, 117, 124**	Anderson 2015*(79)*		no
	Dept. Microbiology & Immunology & Witebsky Center for Microbial Pathogenesis & Immunology, State University of New York	**NR**	Simpson-Abelson 2009*(80)*		no

	Dept. Thoracic and Cardiovascular Surgery, The University of Texas MD Anderson Cancer Center, Houston	**NR**	Hao 2015*(81)*		no
	Dept. Surgical Oncology, University of Texas M. D. Anderson Cancer Center, Houston	**NR**	Roife 2017*(82)*		no

59. T. L. Leong, K. D. Marini, F. J. Rossello, S. N. Jayasekara, P. A. Russell, Z. Prodanovic, B. Kumar, V. Ganju, M. Alamgeer, L. B. Irving, D. P. Steinfort, C. D. Peacock, J. E. Cain, A. Szczepny, D. Neil Watkins, Genomic characterisation of small cell lung cancer patient-derived xenografts generated from endobronchial ultrasound-guided transbronchial needle aspiration specimens, *PLoS ONE* **9**, 1–11 (2014).

60. T. John, D. Kohler, M. Pintilie, N. Yanagawa, N. A. Pham, M. Li, D. Panchal, F. Hui, F. Meng, F. A. Shepherd, M. S. Tsao, The ability to form primary tumor xenografts is predictive of increased risk of disease recurrence in early-stage non-small cell lung cancer, *Clinical Cancer Research* **17**, 134–141 (2011).

61. T. John, N. Yanagawa, D. Kohler, K. J. Craddock, B. Bandarchi-Chamkhaleh, M. Pintilie, J. Sykes, C. To, M. Li, D. Panchal, W. Chen, F. A. Shepherd, M. S. Tsao, Characterization of lymphomas developing in immunodeficient mice implanted with primary human non-small cell lung cancer, *Journal of Thoracic Oncology* **7**, 1101–1108 (2012).

62. C. L. Hann, V. C. Daniel, E. A. Sugar, I. Dobromilskaya, S. C. Murphy, L. Cope, X. Lin, J. S. Hierman, D. L. Wilburn, D. N. Watkins, C. M. Rudin, Therapeutic efficacy of ABT-737, a selective inhibitor of BCL-2, in small cell lung cancer, *Cancer Research* **68**, 2321–2328 (2008).

63. V. C. Daniel, L. Marchionni, J. S. Hierman, J. T. Rhodes, W. L. Devereux, C. M. Rudin, R. Yung, G. Parmigiani, M. Dorsch, C. D. Peacock, D. N. Watkins, A Primary Xenograft Model of Small-Cell Lung Cancer Reveals Irreversible Changes in Gene Expression Imposed by Culture In vitro, *Cancer Research* **69**, 3364–3373 (2009).

64. J. C. Cutz, J. Guan, J. Bayani, M. Yoshimoto, H. Xue, M. Sutcliffe, J. English, J. Flint, J. LeRiche, J. Yee, J. A. Squire, P. W. Gout, S. Lam, Y. Z. Wang, Establishment in severe combined immunodeficiency mice of subrenal capsule xenografts and transplantable tumor lines from a variety of primary human lung cancers: Potential models for studying tumor progression-related changes, *Clinical Cancer Research* **12**, 4043–4054 (2006).

65. X. Dong, J. Guan, J. C. English, J. Flint, J. Yee, K. Evans, N. Murray, C. MacAulay, R. T. Ng, P. W. Gout, W. L. Lam, J. Laskin, V. Ling, S. Lam, Y. Wang, Patient-derived first generation xenografts of non-small cell lung cancers: Promising tools for predicting drug responses for personalized chemotherapy, *Clinical Cancer Research* **16**, 1442–1451 (2010).

66. E. L. Stewart, C. Mascaux, N. A. Pham, S. Sakashita, J. Sykes, L. Kim, N. Yanagawa, G. Allo, K. Ishizawa, D. Wang, C. Q. Zhu, M. Li, C. Ng, N. Liu, M. Pintilie, P. Martin, T. John, I. Jurisica, N. B. Leighl, B. G. Neel, T. K. Waddell, F. A. Shepherd, G. Liu, M. S. Tsao, Clinical utility of patient-derived xenografts to determine biomarkers of prognosis and map resistance pathways in EGFR-mutant lung adenocarcinoma, *Journal of Clinical Oncology* **33**, 2472–2480 (2015).

67. D. D. Fang, B. Zhang, Q. Gu, M. Lira, Q. Xu, H. Sun, M. Qian, W. Sheng, M. Ozeck, Z. Wang, C. Zhang, X. Chen, K. X. Chen, J. Li, S. H. Chen, J. Christensen, M. Mao, C. C. Chan, HIP1-ALK, a novel ALK fusion variant that responds to crizotinib, *Journal of Thoracic Oncology* **9**, 285–294 (2014).

68. Y. Ma, P. Zhang, G. An, X. Zhang, L. Zhang, J. Si, J. Zhang, Y. Yang, Induction of Patient-Derived Xenograft Formation and Clinical Significance of Programmed Cell Death Ligand 1 (PD-L1) in Lung Cancer Patients, *Medical Science Monitor* **22**, 4017–4025 (2016).

69. Y. P. Zhuang, Y. P. Zhu, H. Y. Wang, L. Sun, J. Zhang, Y. P. Hao, L. Wang, Establishment of patient-derived tumor xenograft (PDTX) models using samples from CT-guided percutaneous biopsy, *Brazilian Journal of Medical and Biological Research* **50**, 1–7 (2017).

70. M. Yang, B. Shan, Q. Li, X. Song, J. Cai, J. Deng, L. Zhang, Z. Du, J. Lu, T. Chen, J.-P. Wery, Y. Chen, Q. Li, Overcoming erlotinib resistance with tailored treatment regimen in patient-derived xenografts from naïve Asian NSCLC patients, *International Journal of Cancer* **132**, E74–E84 (2013).

71. M. Ilie, M. Nunes, L. Blot, V. Hofman, E. Long-Mira, C. Butori, E. Selva, A. Merino-Trigo, N. Vénissac, J. Mouroux, P. Vrignaud, P. Hofman, Setting up a wide panel of patient-derived tumor xenografts of non-small cell lung cancer by improving the preanalytical steps, *Cancer Medicine* **4**, 201–211 (2015).

72. I. Fichtner, J. Rolff, R. Soong, J. Hoffmann, S. Hammer, A. Sommer, M. Becker, J. Merk, Establishment of Patient-Derived Non-Small Cell Lung Cancer Xenografts as Models for the Identification of Predictive Biomarkers, *Clinical Cancer Research* **14**, 6456–6468 (2008).

73. J. Merk, J. Rolff, M. Becker, G. Leschber, I. Fichtner, Patient-derived xenografts of non-small-cell lung cancer: a pre-clinical model to evaluate adjuvant chemotherapy?☆, *European Journal of Cardio-Thoracic Surgery* **36**, 454–459 (2009).

74. M. V. Russo, A. Faversani, S. Gatti, D. Ricca, A. Del Gobbo, S. Ferrero, A. Palleschi, V. Vaira, S. Bosari, A New Mouse Avatar Model of Non-Small Cell Lung Cancer, *Frontiers in Oncology* **5**, 1–11 (2015).

75. G. Bertolini, L. Roz, P. Perego, M. Tortoreto, E. Fontanella, L. Gatti, G. Pratesi, A. Fabbri, F. Andriani, S. Tinelli, E. Roz, R. Caserini, S. Lo Vullo, T. Camerini, L. Mariani, D. Delia, E. Calabrò, U. Pastorino, G. Sozzi, Highly tumorigenic lung cancer CD133+ cells display stem-like features and are spared by cisplatin treatment., *Proceedings of the National Academy of Sciences of the United States of America* **106**, 16281–6 (2009).

76. K.-T. Kim, H. W. Lee, H.-O. Lee, S. C. Kim, Y. J. Seo, W. Chung, H. H. Eum, D.-H. Nam, J. Kim, K. M. Joo, W.-Y. Park, Single-cell mRNA sequencing identifies subclonal heterogeneity in anti-cancer drug responses of lung adenocarcinoma cells, *Genome Biology* **16**, 127 (2015).

77. H. S. Seol, Y. A. Suh, Y. J. Ryu, H. J. Kim, S. M. Chun, D. C. Na, H. Fukamachi, S. Y. Jeong, E. K. Choi, S. J. Jang, A patient-derived xenograft mouse model generated from primary cultured cells recapitulates patient tumors phenotypically and genetically, *Journal of Cancer Research and Clinical Oncology* **139**, 1471–1480 (2013).

78. C. Ambrogio, G. Gómez-López, M. Falcone, A. Vidal, E. Nadal, N. Crosetto, R. B. Blasco, P. J. Fernández-Marcos, M. Sánchez-Céspedes, X. Ren, Z. Wang, K. Ding, M. Hidalgo, M. Serrano, A. Villanueva, D. Santamaráa, M. Barbacid, Combined inhibition of DDR1 and Notch signaling is a therapeutic strategy for KRAS-driven lung adenocarcinoma, *Nature Medicine* **22**, 270–277 (2016).

79. W. C. Anderson, M. B. Boyd, J. Aguilar, B. Pickell, A. Laysang, M. A. Pysz, S. Bheddah, J. Ramoth, B. C. Slingerland, S. J. Dylla, E. R. Rubio, Initiation and characterization of small cell lung cancer patient-derived xenografts from ultrasound-guided transbronchial needle aspirates, *PLoS ONE* **10**, 1–13 (2015).

80. M. R. Simpson-Abelson, V. S. Purohit, W. M. Pang, V. Iyer, K. Odunsi, T. L. Demmy, S. J. Yokota, J. L. Loyall, R. J. Kelleher, S. Balu-Iyer, R. B. Bankert, IL-12 delivered intratumorally by multilamellar liposomes reactivates memory T cells in human tumor microenvironments., *Clinical immunology (Orlando, Fla.)* **132**, 71–82 (2009).

81. C. Hao, L. Wang, S. Peng, M. Cao, H. Li, J. Hu, X. Huang, W. Liu, H. Zhang, S. Wu, A. Pataer, J V Heymach, A. K. Eterovic, Q. Zhang, K. R. Shaw, K. Chen, A. Futreal, M. Wang, W. Hofstetter, R. Mehran, D. Rice, J. A. Roth, B. Sepesi, S. G. Swisher, A. Vaporciyan, G. L. Walsh, F. M. Johnson, B. Fang, Gene mutations in primary tumors and corresponding patient-derived xenografts derived from non-small cell lung cancer., *Cancer letters* **357**, 179–185 (2015).

82. D. Roife, Y. Kang, L. Wang, B. Fang, S. G. Swisher, J. E. Gershenwald, S. Pretzsch, C. P. Dinney, M. H. G. Katz, J. B. Fleming, Generation of patient-derived xenografts from fine needle aspirates or core needle biopsy, *Surgery* **161**, 1246–1254 (2017).

D. Prostate

Country	Primary Laboratory location	PDX ID	First Author Surname and Year	Associated refs	ARRIVE guidelines?
Australia	Australian Prostate Cancer Research Centre	**UCRU-PR-1, -2, -4**	Russell 2015*(83)*	Jelbart 1988*(84)*; Pittman 1987*(85)*	no
	Dept. of Anatomy & Developmental Biology, Monash University	**NR**	Lawrence 2015*(86)*		no
			Risbridger 2015*(87)*	Toivanen 2013	no
	Prostate and Breast Cancer Research Group, Monash University	**NR**	Toivanen 2011*(88)*		no
Canada	Dept. Cancer Endocrinology, BC Cancer Agency, Vancouver	**PCa1**	Wang 2005*(89)*		no
	Vancouver Prostate Centre & Department of Urologic Sciences, University of British Columbia	**LTLxxx**	Lin 2013*(90)*		no
Japan	Dept. Urology, Kyoto University Graduate School of Medicine	**KUCaP-2**	Terada 2010*(91)*		no
		**KUCaP-3**	Yoshikawa 2016*(92)*		no
Switzerland	Dept. Urology, University Hospital Basel	**NR**	Wetterauer 2015*(93)*		no
Netherlands	Depts. of Urology and Pathology, Erasmus University	**PC-XXX**	van Weerden 1996*(94)*		no
USA	Dept. Genitourinary Medical Oncology, The University of Texas M. D. Anderson Cancer Center	**MDA 144-2; -4; -6; -9;**	Aparicio 2011*(95)*	Aparicio 2016*(96)*, Tzelepi 2012*(97)*	no
		**MDA 163, MDA 166, MDA 177, MDA 189, MDA 205**	Aparicio 2016*(96)*		no
		**MDA 79, MDA 117, MDA 130, MDA 146, MDA 155, MDA 170, MDA 180**	Tzelepi 2012*(97)*	Aparicio 2011*(95)*	no
	Dept. of Medical Oncology, Dana Farber Cancer Institute, Brigham and Women's Hospital, Harvard Medical School	**NR**	Priolo 2010*(98)*		no
	Dept. Urology, Case Western Reserve University School of Medicine	**CWR21, 22, 31, 91**	Pretlow 1993*(99)*	Wainstein 1994*(100)*	no
	Depts. Medicine, University of California	**LAPC3, -6, -7**	Klein 1997*(101)*		no
	Depts. Pathology and Laboratory Medicine and Surgery, University of North Carolina-Lineberger Comprehensive Cancer Center, University of North Carolina	**xT**	Presnell 2001*(102)*		no
	Dept. Urology, University of California Irvine	**GM0308, RC0309**	Li 2012*(103)*		no
	MD Anderson Cancer Centre	**HPCa xx**	Chen 2013*(104)*		no

83. P. J. Russell, P. Russell, C. Rudduck, B. W. C. Tse, E. D. Williams, D. Raghavan, Establishing prostate cancer patient derived xenografts: Lessons learned from older studies, *Prostate* **75**, 628–636 (2015).

84. M. E. Jelbart, P. J. Russell, M. Fullerton, P. Russell, J. Funder, D. Raghavan, Ectopic hormone production by a prostatic small cell carcinoma xenograft line., *Molecular and cellular endocrinology* **55**, 167–72 (1988).

85. S. Pittman, P. J. Russell, M. E. Jelbart, J. Wass, D. Raghavan, Flow cytometric and karyotypic analysis of a primary small cell carcinoma of the prostate: A xenografted cell line, *Cancer Genetics and Cytogenetics* **26**, 165–169 (1987).

86. M. G. Lawrence, D. W. Pook, H. Wang, L. H. Porter, M. Frydenberg, J. Kourambas, S. Appu, C. Poole, E. K. Beardsley, A. Ryan, S. Norden, M. M. Papargiris, G. P. Risbridger, R. A. Taylor, Establishment of primary patient-derived xenografts of palliative TURP specimens to study castrate-resistant prostate cancer, *The Prostate* **75**, 1475–1483 (2015).

87. G. P. Risbridger, R. A. Taylor, D. Clouston, A. Sliwinski, H. Thorne, S. Hunter, J. Li, G. Mitchell, D. Murphy, M. Frydenberg, D. Pook, J. Pedersen, R. Toivanen, H. Wang, M. Papargiris, M. G. Lawrence, D. M. Bolton, Patient-derived xenografts reveal that intraductal carcinoma of the prostate is a prominent pathology in brca2 mutation carriers with prostate cancer and correlates with poor prognosis, *European Urology* **67**, 496–503 (2015).

88. R. Toivanen, D. M. Berman, H. Wang, J. Pedersen, M. Frydenberg, A. K. Meeker, S. J. Ellem, G. P. Risbridger, R. A. Taylor, Brief report: A bioassay to identify primary human prostate cancer repopulating cells, *Stem Cells* **29**, 1310–1314 (2011).

89. Y. Wang, H. Xue, J.-C. Cutz, J. Bayani, N. R. Mawji, W. G. Chen, L. J. Goetz, S. W. Hayward, M. D. Sadar, C. B. Gilks, P. W. Gout, J. A. Squire, G. R. Cunha, Y.-Z. Wang, An orthotopic metastatic prostate cancer model in SCID mice via grafting of a transplantable human prostate tumor line, *Laboratory Investigation* **85**, 1392–1404 (2005).

90. D. Lin, A. W. Wyatt, H. Xue, Y. Wang, X. Dong, A. Haegert, R. Wu, S. Brahmbhatt, F. Mo, L. Jong, R. H. Bell, S. Anderson, A. Hurtado-Coll, L. Fazli, M. Sharma, H. Beltran, M. Rubin, M. Cox, P. W. Gout, J. Morris, L. Goldenberg, S. V. Volik, M. E. Gleave, C. C. Collins, Y. Wang, High Fidelity Patient-Derived Xenografts for Accelerating Prostate Cancer Discovery and Drug Development, *Cancer Research* **74**, 1272–1283 (2014).

91. N. Terada, Y. Shimizu, T. Kamba, T. Inoue, A. Maeno, T. Kobayashi, E. Nakamura, T. Kamoto, T. Kanaji, T. Maruyama, Y. Mikami, Y. Toda, T. Matsuoka, Y. Okuno, G. Tsujimoto, S. Narumiya, O. Ogawa, Identification of EP4 as a potential target for the treatment of castration-resistant prostate cancer using a novel xenograft model, *Cancer Research* **70**, 1606–1615 (2010).

92. T. Yoshikawa, G. Kobori, T. Goto, S. Akamatsu, N. Terada, T. Kobayashi, Y. Tanaka, G. Jung, T. Kamba, O. Ogawa, T. Inoue, An original patient-derived xenograft of prostate cancer with cyst formation, *Prostate* **76**, 994–1003 (2016).

93. C. Wetterauer, T. Vlajnic, J. Schüler, J. R. Gsponer, G. N. Thalmann, M. Cecchini, J. Schneider, T. Zellweger, H. Pueschel, A. Bachmann, C. Ruiz, S. Dirnhofer, L. Bubendorf, C. A. Rentsch, Early development of human lymphomas in a prostate cancer xenograft program using triple knock-out Immunocompromised mice, *Prostate* **75**, 585–592 (2015).

94. W. M. van Weerden, C. M. de Ridder, C. L. Verdaasdonk, J. C. Romijn, T. H. van der Kwast, F. H. Schröder, G. J. van Steenbrugge, Development of seven new human prostate tumor xenograft models and their histopathological characterization., *The American journal of pathology* **149**, 1055–62 (1996).

95. A. Aparicio, V. Tzelepi, J. C. Araujo, C. C. Guo, S. Liang, P. Troncoso, C. J. Logothetis, N. M. Navone, S. N. Maity, Neuroendocrine prostate cancer xenografts with large-cell and small-cell features derived from a single patient's tumor: Morphological, immunohistochemical, and gene expression profiles, *The Prostate* **71**, 846–856 (2011).

96. A. M. Aparicio, L. Shen, E. L. N. Tapia, J.-F. Lu, H.-C. Chen, J. Zhang, G. Wu, X. Wang, P. Troncoso, P. Corn, T. C. Thompson, B. Broom, K. Baggerly, S. N. Maity, C. J. Logothetis, Combined Tumor Suppressor Defects Characterize Clinically Defined Aggressive Variant Prostate Cancers, *Clinical Cancer Research* **22**, 1520–1530 (2016).

97. V. Tzelepi, J. Zhang, J.-F. Lu, B. Kleb, G. Wu, X. Wan, A. Hoang, E. Efstathiou, K. Sircar, N. M. Navone, P. Troncoso, S. Liang, C. J. Logothetis, S. N. Maity, A. M. Aparicio, Modeling a Lethal Prostate Cancer Variant with Small-Cell Carcinoma Features, *Clinical Cancer Research* **18**, 666–677 (2012).

98. C. Priolo, M. Agostini, N. Vena, A. H. Ligon, M. Fiorentino, E. Shin, A. Farsetti, A. Pontecorvi, E. Sicinska, M. Loda, Establishment and Genomic Characterization of Mouse Xenografts of Human Primary Prostate Tumors, *The American Journal of Pathology* **176**, 1901–1913 (2010).

99. T. G. Pretlow, S. R. Wolman, M. a Micale, R. J. Pelley, E. D. Kursh, M. I. Resnick, D. R. Bodner, J. W. Jacobberger, C. M. Delmoro, J. M. Giaconia, Xenografts of primary human prostatic carcinoma., *Journal of the National Cancer Institute* **85**, 394–8 (1993).

100. M. A. Wainstein, F. He, D. Robinson, H. J. Kung, S. Schwartz, J. M. Giaconia, N. L. Edgehouse, T. P. Pretlow, D. R. Bodner, E. D. Kursh, CWR22: androgen-dependent xenograft model derived from a primary human prostatic carcinoma., *Cancer research* **54**, 6049–52 (1994).

101. K. A. Klein, R. E. Reiter, J. Redula, H. Moradi, X. L. Zhu, A. R. Brothman, D. J. Lamb, M. Marcelli, A. Belldegrun, O. N. Witte, C. L. Sawyers, Progression of metastatic human prostate cancer to androgen independence in immunodeficient SCID mice., *Nature medicine* **3**, 402–8 (1997).

102. S. C. Presnell, E. S. Werdin, S. Maygarden, J. L. Mohler, G. J. Smith, Establishment of short-term primary human prostate xenografts for the study of prostate biology and cancer, *American Journal of Pathology* **159**, 855–860 (2001).

103. X. Li, Z. Liu, X. Xu, C. A. Blair, Z. Sun, J. Xie, M. B. Lilly, X. Zi, C. Creighton, Ed. Kava Components Down-Regulate Expression of AR and AR Splice Variants and Reduce Growth in Patient-Derived Prostate Cancer Xenografts in Mice, *PLoS ONE* **7**, e31213 (2012).

104. X. Chen, B. Liu, Q. Li, S. Honorio, X. Liu, C. Liu, A. S. Multani, T. Calhoun-Davis, D. G. Tang, Dissociated Primary Human Prostate Cancer Cells Coinjected with the Immortalized Hs5 Bone Marrow Stromal Cells Generate Undifferentiated Tumors in NOD/SCID-γ Mice, *PLoS ONE* **8** (2013), doi:10.1371/journal.pone.0056903.

Breast, colon, lung, prostate

Study Tumour Type	Country	Laboratory location	PDX ID	First Author Surname and Year	Associated refs	ARRIVE guidelines?
Breast; Colon; Lung; Prostate	Singapore	PharmaLogicals Research Pte. Ltd.	NR	Fujii 2008*(105)*		no
Breast; Colon	USA	Center for Developmental Therapeutics, Northwestern University	NR	Bondarenko 2015*(106)*		no
Colon; Lung	USA	Department of Immunology, Roswell Park Cancer Institute, NY	Colo205; NR	Hylander 2013*(107)*		no
Breast; Colon; Lung	France	Institut de Cancerologieet d'Immunogenetique, Paris	NR	Levi 1984*(108)*		no

105. E. Fujii, M. Suzuki, K. Matsubara, M. Watanabe, Y. J. Chen, K. Adachi, Y. Ohnishi, M. Tanigawa, M. Tsuchiya, N. Tamaoki, Establishment and characterization of in vivo human tumor models in the NOD/SCID/gamma(c)(null) mouse., *Pathology international* **58**, 559–567 (2008).

106. G. Bondarenko, A. Ugolkov, S. Rohan, P. Kulesza, O. Dubrovskyi, D. Gursel, J. Mathews, T. V. O'Halloran, J. J. Wei, A. P. Mazar, Patient-derived tumor xenografts are susceptible to formation of human lymphocytic tumors, *Neoplasia* **17**, 735–741 (2015).

107. B. L. Hylander, N. Punt, H. Tang, J. Hillman, M. Vaughan, W. Bshara, R. Pitoniak, E. A. Repasky, Origin of the vasculature supporting growth of primary patient tumor xenografts, *Journal of Translational Medicine* **11**, 1–14 (2013).

108. F. A. Levi, J. P. Blum, C. Bourut, G. Mathá, G. Lemaigre, A. Reinberg, A Four-Day Subrenal Capsule Assay for Testing the Effectiveness of Anticancer Drugs Against Human Tumors, *Cancer Research* **44**, 2660–2667 (1984).
