# Supplementary material for: A systematic review of the validity of patient derived xenograft (PDX) models: the implications for translational research and personalised medicine
Source: PeerJ. 2018 Nov 21;6:e5981. doi: 10.7717/peerj.5981 (PMC6252062; doi:10.7717/peerj.5981)
Supplement: Table S6 [file peerj-06-5981-s006.pdf]

Supplemental Table S6. Studies that did not validate all models

Tissue	No. of validated models (justification)	Study
Breast	5 of 7	Wakasugi 1995*(1)*
	21 of 35	Cottu 2012*(2)*
	8 of 14	Valdez 2011*(3)*
	5 of 8	Visonneau 1998*(4)*
	5 of 17	Morancho 2016*(5)*
Colon	1 of 12	Jin 2011*(6)*
	2 of 5. Results not reported for any validation	Davies 1981*(7)*
	4 of 9	Dangles-Marie 2007*(8)*
	3 of 9	Hylander 2013*(9)*
	2 of 80 PDX or 2 of 10 tissues	Gock 2016*(10)*
	4 of 17	Chou 2013*(11)*
	9 of 150	Oh 2015*(12)*
	14 of 16	Burgenske 2014*(13)*
	27 of 198	Linnebacher 2010*(14)*
	1 of 10	Szabo 2013*(15)*
	26 of 27	Chen 2015*(16)*
Lung	11 of 1573	Dong 2010*(17)*
	1 of 20	Simpson-Abelson 2009*(18)*
	4 of 29	Russo 2015*(19)*
	4 of 6	Stewart 2015*(20)*
	1 of 9	Roife 2017*(21)*
	5 of 10	Bertolini 2009*(22)*
	2 of 5	Hylander 2013*(9)*
Prostate	8 of 19	Aparicio 2016*(23)*
	10 of 24	Lawrence 2015*(24)*

1. H. Wakasugi, K. Koyama, M. Gyotoku, M. Yoshimoto, S. Hirohashi, T. Sugimura, M. Terada, Frequent Development of Murine T-Cell Lymphomas with TcRα/β+, CD4-/8 Phenotype after Implantation of Human Inflammatory Breast Cancer Cells in BALB/c Nude Mice*Japanese Journal of Cancer Research* **86**, 1086–1096 (1995).

2. P. Cottu, E. Marangoni, F. Assayag, P. De Cremoux, A. Vincent-Salomon, C. Guyader, L. De Plater, C. Elbaz, N. Karboul, J. J. Fontaine, S. Chateau-Joubert, P. Boudou-Rouquette, S. Alran, V. Dangles-Marie, D. Gentien, M. F. Poupon, D. Decaudin, Modeling of response to endocrine therapy in a panel of human luminal breast cancer xenografts, *Breast Cancer Research and Treatment* **133**, 595–606 (2012).

3. K. E. Valdez, F. Fan, W. Smith, D. C. Allred, D. Medina, F. Behbod, Human primary ductal carcinoma in situ (DCIS) subtype-specific pathology is preserved in a mouse intraductal (MIND) xenograft model, *The Journal of Pathology* **225**, 565–573 (2011).

4. S. Visonneau, A. Cesano, M. H. Torosian, E. J. Miller, D. Santoli, Growth Characteristics and Metastatic Properties of Human Breast Cancer Xenografts in Immunodeficient Mice, *American Journal of Pathology* **152**, 1299–1311 (1998).

5. B. Morancho, M. Zacarias-Fluck, A. Esgueva, C. Bernado-Morales, S. Di Cosimo, A. Prat, J. Cortes, J. Arribas, I. T. Rubio, Modeling anti-IL-6 therapy using breast cancer patient-derived xenografts,

Oncotarget **7**, 67956–67965 (2016).

6. K. Jin, G. Li, B. Cui, J. Zhang, H. Lan, N. Han, B. Xie, F. Cao, K. He, H. Wang, Z. Xu, L. Teng, T. Zhu, Assessment of a novel vegf targeted agent using patient-derived tumor tissue xenograft models of colon carcinoma with lymphatic and hepatic metastases, *PLoS ONE* **6** (2011), doi:10.1371/journal.pone.0028384.

7. G. Davies, D. Duke, A. G. Grant, S. A. Kelly, J. Hermon-Taylor, Growth of human digestive-tumour xenografts in athymic nude rats, *British Journal of Cancer* **43**, 53–58 (1981).

8. V. Dangles-Marie, M. Pocard, S. Richon, L. B. Weiswald, F. Assayag, P. Saulnier, J. G. Judde, J. L. Janneau, N. Auger, P. Validire, B. Dutrillaux, F. Praz, D. Bellet, M. F. Poupon, Establishment of human colon cancer cell lines from fresh tumors versus xenografts: Comparison of success rate and cell line features, *Cancer Research* **67**, 398–407 (2007).

9. B. L. Hylander, N. Punt, H. Tang, J. Hillman, M. Vaughan, W. Bshara, R. Pitoniak, E. A. Repasky, Origin of the vasculature supporting growth of primary patient tumor xenografts, *Journal of Translational Medicine* **11**, 1–14 (2013).

10. M. Gock, F. Kühn, C. S. Mullins, M. Krohn, F. Prall, E. Klar, M. Linnebacher, Tumor Take Rate Optimization for Colorectal Carcinoma Patient-Derived Xenograft Models, *BioMed Research International* **2016** (2016), doi:10.1155/2016/1715053.

11. J. Chou, M. P. Fitzgibbon, C. L. L. Mortales, A. M. H. Towlerton, M. P. Upton, R. S. Yeung, M. W. McIntosh, E. H. Warren, Phenotypic and transcriptional fidelity of patient-Derived colon cancer xenografts in immune-deficient mice, *PLoS ONE* **8** (2013), doi:10.1371/journal.pone.0079874.

12. B. Y. Oh, W. Y. Lee, S. Jung, H. K. Hong, D.-H. Nam, Y. A. Park, J. W. Huh, S. H. Yun, H. C. Kim, H.-K. Chun, Y. B. Cho, Correlation between tumor engraftment in patient-derived xenograft models and clinical outcomes in colorectal cancer patients., *Oncotarget* **6**, 16059–68 (2015).

13. D. M. Burgenske, D. J. Monsma, D. Dylewski, S. B. Scott, A. D. Sayfie, D. G. Kim, M. Luchtefeld, K. R. Martin, P. Stephenson, G. Hostetter, N. Dujovny, J. P. MacKeigan, Establishment of genetically diverse patient-derived xenografts of colorectal cancer., *American journal of cancer research* **4**, 824–37 (2014).

14. M. Linnebacher, C. Maletzki, C. Ostwald, U. Klier, M. Krohn, E. Klar, F. Prall, Cryopreservation of human colorectal carcinomas prior to xenografting, *BMC Cancer* **10**, 362 (2010).

15. C. Szabo, C. Coletta, C. Chao, K. Modis, B. Szczesny, A. Papapetropoulos, M. R. Hellmich, Tumor-derived hydrogen sulfide, produced by cystathionine- -synthase, stimulates bioenergetics, cell proliferation, and angiogenesis in colon cancer, *Proceedings of the National Academy of Sciences* **110**, 12474–12479 (2013).

16. D. Chen, X. Huang, J.-P. Wery, Q.-X. Li, J. Cai, S. Guo, W. Qian, J.-P. Wery, Q.-X. Li, A set of defined oncogenic mutation alleles seems to better predict the response to cetuximab in CRC patient-derived xenograft than KRAS 12/13 mutations, *Oncotarget* **6**, 40815–40821 (2015).

17. X. Dong, J. Guan, J. C. English, J. Flint, J. Yee, K. Evans, N. Murray, C. MacAulay, R. T. Ng, P. W. Gout, W. L. Lam, J. Laskin, V. Ling, S. Lam, Y. Wang, Patient-derived first generation xenografts of non-small cell lung cancers: Promising tools for predicting drug responses for personalized chemotherapy, *Clinical Cancer Research* **16**, 1442–1451 (2010).

18. M. R. Simpson-Abelson, V. S. Purohit, W. M. Pang, V. Iyer, K. Odunsi, T. L. Demmy, S. J. Yokota, J. L. Loyall, R. J. Kelleher, S. Balu-Iyer, R. B. Bankert, IL-12 delivered intratumorally by multilamellar liposomes reactivates memory T cells in human tumor microenvironments., *Clinical immunology (Orlando, Fla.)* **132**, 71–82 (2009).

19. M. V. Russo, A. Faversani, S. Gatti, D. Ricca, A. Del Gobbo, S. Ferrero, A. Palleschi, V. Vaira, S. Bosari,

A New Mouse Avatar Model of Non-Small Cell Lung Cancer, *Frontiers in Oncology* **5**, 1–11 (2015).

20. E. L. Stewart, C. Mascaux, N. A. Pham, S. Sakashita, J. Sykes, L. Kim, N. Yanagawa, G. Allo, K. Ishizawa, D. Wang, C. Q. Zhu, M. Li, C. Ng, N. Liu, M. Pintilie, P. Martin, T. John, I. Jurisica, N. B. Leighl, B. G. Neel, T. K. Waddell, F. A. Shepherd, G. Liu, M. S. Tsao, Clinical utility of patient-derived xenografts to determine biomarkers of prognosis and map resistance pathways in EGFR-mutant lung adenocarcinoma, *Journal of Clinical Oncology* **33**, 2472–2480 (2015).

21. D. Roife, Y. Kang, L. Wang, B. Fang, S. G. Swisher, J. E. Gershenwald, S. Pretzsch, C. P. Dinney, M. H. G. Katz, J. B. Fleming, Generation of patient-derived xenografts from fine needle aspirates or core needle biopsy, *Surgery* **161**, 1246–1254 (2017).

22. G. Bertolini, L. Roz, P. Perego, M. Tortoreto, E. Fontanella, L. Gatti, G. Pratesi, A. Fabbri, F. Andriani, S. Tinelli, E. Roz, R. Caserini, S. Lo Vullo, T. Camerini, L. Mariani, D. Delia, E. Calabrò, U. Pastorino, G. Sozzi, Highly tumorigenic lung cancer CD133+ cells display stem-like features and are spared by cisplatin treatment., *Proceedings of the National Academy of Sciences of the United States of America* **106**, 16281–6 (2009).

23. C. Ambrogio, G. Gómez-López, M. Falcone, A. Vidal, E. Nadal, N. Crosetto, R. B. Blasco, P. J. Fernández-Marcos, M. Sánchez-Céspedes, X. Ren, Z. Wang, K. Ding, M. Hidalgo, M. Serrano, A. Villanueva, D. Santamaráa, M. Barbacid, Combined inhibition of DDR1 and Notch signaling is a therapeutic strategy for KRAS-driven lung adenocarcinoma, *Nature Medicine* **22**, 270–277 (2016).

24. M. G. Lawrence, D. W. Pook, H. Wang, L. H. Porter, M. Frydenberg, J. Kourambas, S. Appu, C. Poole, E. K. Beardsley, A. Ryan, S. Norden, M. M. Papargiris, G. P. Risbridger, R. A. Taylor, Establishment of primary patient-derived xenografts of palliative TURP specimens to study castrate-resistant prostate cancer, *The Prostate* **75**, 1475–1483 (2015).
