# Supplementary material for: A systematic review of the validity of patient derived xenograft (PDX) models: the implications for translational research and personalised medicine
Source: PeerJ. 2018 Nov 21;6:e5981. doi: 10.7717/peerj.5981 (PMC6252062; doi:10.7717/peerj.5981)
Supplement: Table S7 [file peerj-06-5981-s007.pdf]

Supplemental Table S7: Primary Outgrowth Rates for PDX Models

A. Breast PDX (primary tumours only)

Study	Sample type	Mouse strain	Engraftment site	Donor pathology	Donor treatment	N sample	Outgrowth rate (%)	N	Definition of outcome
**Shirakawa 2001**	**TF**	**Balb/c nude, SCID**	**subcutaneous**	**High grade inv DC (100%)**	**NR**	**10**	**10**	**patient**	**exponential growth**
Zhang 2013	TF	SCID/Bg	orthotopic	IDC (89%)	Previous treatment	169	41	patient	A surviving tissue fragment > 1 mm in diameter and shown to be proliferative (Ki67+)
Valdez 2011*	IC	NSG	orthotopic	DC (100%)	NR	8	75	patient	8 weeks growth
Valdez 2011*	IC	NSG	orthotopic	DC (100%)	NR	34	42	tissue	8 weeks growth
Bergamaschi 2009*	TF	SCID	subcutaneous	IDC (76%); ILC (14%); MUC (10%)	NR	30	20	PDX	P0-P1. A diameter of approximately 15 mm.
Charafe-Jauffret 2013	IC	NSG	orthotopic	IDC (83.2%); ILC (7%); mixed (1.4%); MED (1.4%); other (7%)	CT (76%); IR (80%); HT (55%)	74	27	tissue	12 mm in the largest diameter (<1000 mm3),
Visonneau 1998	TF	SCID, NOD/SCID	subcutaneous	Infiltrating carcinoma (13%); IDC (74%); mixed (13%)	NR	16	50	patient	Signs of heavy tumor burden (> 10% body weight), ulceration of the skin with bleeding, and/or signs of severe respiratory distress, weakness and lethargy.
**Patsialou 2012**	**TF**	**SCID**	**orthotopic**	**ILC (13%), IDC (87%)**	**NR**	**29**	**27.6**	**tissue**	**samples that grew from 1st implantation**
**McAuliffe 2015**	**TF**	**Balb/c nude**	**subcutaneous**	**NR**	**Neoadjuvant therapy (50%)**	**48**	**27**	**tissue**	**tumor growth at the site of implantation**
Cottu 2012	TF	Swiss nude	orthotopic	NR	NR	164	15	patient	NR
**du Manoir 2014***	**TF**	**Swiss nude**	**subcutaneous**	**NR**	**NR**	**130**	**30**	**patient**	**A maximum of 2000 mm3**
Eyre 2016*	TF	NSG	subcutaneous	NR	RT (2%), CT (4%), HT (13%)	120	38	tissue	1.3cm3

*possible oestrogen supplementation. TF = tissue fragments; IC = isolated cells. IDC = invasive ductal carcinoma; ILC = invasive lobular carcinoma; DC = ductal carcinoma; mets = metastases; AC = adenocarcinoma; inv= invasive; MUC = mucinous; MED = medullary. CT = chemotherapy; IR = irradiation; HT = hormone therapy. Bold = studies with unclear concerns for model validity; grey shade = studies with high risk of concern for model validity.

B. Colon PDX (primary tumours only, all subcutaneous using tissue fragments unless otherwise indicated)

Study	Mouse strain	Donor pathology	Donor Treatment	N sample	Outgrowth rate (%)	N	Definition of outcome
**Davies 1981**	**nude mice**	**AC (100%)**	**NR**	**9**	**44**	**PDX**	**NR**
**Lee 2014**	**Balb/c nude or NOD/SCID**	**AC (100%)**	**TN**	**10**	**100**	**patient**	**1000 mm3**
**Mukohyama 2016**	**NOD/SCID**	**AC (100%)**	**NR**	**5**	**60**	**patient**	**1-2 cm**
**Mukohyama 2016****	**NSG**	**AC (100%)**	**NR**	**8**	**50**	**patient**	**1-2 cm**
**Zhou 2011**	**Balb/c nude**	**AC (100%)**	**NR**	**21**	**14**	**patient**	**reached 1 cm diameter**
**Jin 2011**	**Balb/c nude**	**mAC (100%)**	**NR**	**20**	**60**	**PDX**	**>1500 mm3**
**Chou 2013***	**NSG**	**AC (65%), mAC (23%), AC/NE (5%), NE (5%)**	**CT (18%)**	**28**	**61**	**patient**	**2 cm**
**Guan 2016**	**Balb/c nude**	**AC (89%), mAC (11%)**		**85**	**58.5**	**patient**	**150mm3**
**Linnebacher 2010**	**NOD/SCID**	**AC (98%); LNEC (2%)**	**TN**	**48**	**27**	**patient**	**reached 1-1.5cm3**
Puig 2013*	NOD/SCID	AC (75%); mAC (25%)	CT (50%)	32	84	patient	reached 1cm3
**Zhang 2015**	**NOD/SCID**	**AC (97%), mAC (3%)**	**CT (3%)**	**43**	**70**	**patient**	**500-1000 mm3**
**Dangles-Marie 2007**	**Swiss nude**	**NR**	**NR**	**11**	**82**	**patient**	**NR**
**Gock 2016**	**NMRI nude**	**NR**	**CT naïve**	**20**	**40**	**Tissue**	**1-1.5cm3**
**Julien 2012**	**Swiss nude**	**NR**	**CT (23%)**	**58**	**60**	**patient**	**800 -1500 mm3**

*Isolated cells, subrenal and subcutaneous. ** minced tissue. AC = adenocarcinoma; mAC=mucinous adenocarcinoma; CaG=carcinoma gingiva; Ca= carcinoid; LNEC = large cell neuroendocrine carcinoma; NE = neuroendocrine ; squamous cell carcinoma =SCC, combined small cell carcinoma =SCC; denosquamous =DS, pleomorphic carcinoma =PC . CT = chemotherapy; TN = treatment naïve. Bold = studies with unclear concerns for model validity; grey shade = studies with high risk of concern for model validity.

C. Lung PDX (primary tumours only, all tissue fragments)

Study	Mouse strain	Engraftment site	Donor pathology	Donor treatment	N sample	Outgrowth rate (%)	N	Definition of outcome
Anderson	NOD/SCID	orthotopic	100% SCC	NR	10	60	patient	confirmed SCLC tumours (800-1500mm3)
Russo 2015	CD1 nude	subrenal	100% SQCC	TN	12	92	PDX	first generation xenografts which closely resembled the parental tumour by H &E
Russo 2015	CD1 nude	subrenal	100% AC	TN	14	29	PDX	first generation xenografts which closely resembled the parental tumour by H &E
**Dong 2010**	**NOD/SCID**	**subrenal**	**100% NSCLC**	**TN**	**527**	**90**	**PDX**	**49 days growth**

**Roife 2017**	**NOD/SCID**	**subcutaneous**	**100% NSCLC**	**Treatment (25%)**	**16**	**50**	**patient**	**1.5cm3 tumour**
**Cutz, 2006**	**NOD/SCID**	**subrenal**	**7% squamous metaplasia; 7% carcinoid; 35% SQCC; 14% AC; 21% SCC; 7% carcinosarcoma**	**21% CT, 14% RT**	**14**	**78**	**patient**	**First-generation implant growth was assessed by palpation and harvested at 30 days or up to 60 days postgrafting**
**Hao 2015**	**NOD/SCID**	**subcutaneous**	**48% AC, 39% SQCC, 13% NE**	**NR**	**88**	**26**	**patient**	**Tumours which reached 1.5cm3**
**Zhang 2013**	**SCID, nude**	**subcutaneous**	**52% AC; 3% AC/SCC; 45% SCC**	**TN**	**31**	**45**	**patient**	**first generation of mice**
**Zhuang 2017**	**NCG**	**subcutaneous**	**44% AC; 33% SCC; 22% SQCC**	**NR**	**9**	**33**	**patient**	**$500m^3$ tumour**
**Bertolini 2009**	**CD1 nude**	**subcutaneous**	**80% AC; 20% LCC**		**29**	**34**	**tissue**	**NR**

SCC = Small cell carcinoma; NSCLC = non-small cell lung cancer; AC = Adenocarcinoma; LCC = Large cell carcinoma; ASQC = Adenosquamous carcinoma; DC = ductal carcinoma; SQCC = squamous cell carcinoma; NE = neuroendocrine. CT = chemotherapy; RT = radiotherapy. TN = treatment naïve. Bold = studies with unclear concerns for model validity; grey shade = studies with high risk of concern for model validity.

D. Prostate PDX (primary tumours only, all tissue fragments)

Study	Mouse strain	Engraftment site	Donor pathology	Donor treatment	N sample	Outgrowth rate (%)	N	Definition of outcome
**Priolo 2010**	**nude, NOD/SCID**	**subrenal**	**100% AC**	**TN**	**23**	**56**	**patient**	**grafts evaluated 3 months after grafting**
**Priolo 2010**	**nude, NOD/SCID**	**orthotopic**	**100% AC**	**TN**	**15**	**0**	**patient**	**grafts evaluated 3 months after grafting**
Wang 2005	NOD/SCID	subrenal	100% AC		1	100	patient	60 or 90 days of growth
Russell 2015	nude	subcutaneous	66% AC, 33% AC/SCC	66% HT; 33% TN	29	17	patient	5–10 mm in diameter
Lawrence 2015	NOD/SCID, NSG	subrenal	20% HR, 80% CRPC	ADT (100%) and either RT, CT, abiratarone or enzalutamide	112	21	PDX	6-16 weeks growth
Lawrence 2015	NOD/SCID, NSG	subrenal	20% HR, 80% CRPC	100% had ADT and combinations of either RT, CT , abiratarone or enzalutamide	10	70	patient	6-16 weeks growth
Risbridger 2015	NSG	subrenal	100% G7	HN	62	8	PDX	Grafts with intraductal CAP evaluated between 6-14 wks after grafting.
Risbridger 2015	NSG	subrenal	100% G7	HN	12	17	patient	Grafts with intraductal CAP evaluated between 6-14 wks after grafting

Risbridger 2015	NSG	subrenal	100% G7	HN	62	71	PDX	Grafts with AC evaluated between 6-14 wks after grafting.
Risbridger 2015	NSG	subrenal	100% G7	HN	12	100	patient	Grafts with AC evaluated between 6-14 wks after grafting
Risbridger 2015*	NOD/SCID, NSG	subrenal	100% G7	HN	191	43	PDX	grafts containing tumor foci
Risbridger 2015*	NOD/SCID, NSG	subrenal	100% Gleason 7	HN	12	100	patient	grafts containing tumor foci
**Toivanen 2011**	**NOD/SCID**	**subrenal**	**100% G7**	**NR**	**41**	**41**	**PDX**	**grafts containing tumor foci**
**Toivanen 2011**	**NOD/SCID**	**subrenal**	**100% G7**	**NR**	**6**	**67**	**patient**	**grafts containing tumor foci**
**Wetterauer 2015**	**NSG, NOG**	**subcutaneous, subrenal**	**70% G7, 20% G9, 10% G8**	**NR**	**27**	**37**	**patient**	**Assessed 3 months after grafting. Rate includes lymphoma development**
**Pretlow 1993**	**nude**	**subcutaneous**	**50% G9, 25% G7, 25% G8**	**NR**	**49**	**33**	**PDX**	**Tumors reaching a diameter of 4mm**
**Pretlow 1993**	**nude**	**subcutaneous**	**50% G9, 25% G7, 25% G8**	**NR**	**20**	**30**	**patient**	**Tumors reaching a diameter of 4mm**
**Presnell 2001**	**nude**	**subcutaneous**	**60% G6, 40% G8**	**NR**	**5**	**100**	**PDX**	**Assessed 1 month after grafting**
**Klein 1997**	**SCID**	**subcutaneous**	**66% T3, 33% T4**	**66% ADT**	**3**	**66**	**patient**	**2 to 3-fold increase in size**
Chen 2013	NOD/SCID	subrenal	NR	TN	112	30	PDX	palpable tumour
Chen 2013	Rag2	subrenal	NR	TN	2	100	PDX	palpable tumour
Chen 2013	NOD/SCID	subcutaneous	NR	TN	228	69	PDX	palpable tumour
Chen 2013	Rag2	subcutaneous	NR	TN	32	63	PDX	palpable tumour
Chen 2013	NSG	subcutaneous	NR	TN	89	75	PDX	palpable tumour
Chen 2013	NOD/SCID	orthotopic	NR	TN	55	18	PDX	palpable tumour
**Yoshikawa 2016**	**SCID**	**subcutaneous, subrenal**	**NR**	**100% ADT, CT, RT**	**5**	**25**	**PDX**	**NR**
van Weerden 1996	NMRI nude	subcutaneous	NR	80% TN, 20% ADT	13	38.5	patient	primary take rate
van Weerden 1996	BALB/c nude	subcutaneous	NR	80% TN, 20% ADT	25	0	patient	primary take rate

*isolated cells. AC = adenocarcinoma; SCC = small cell carcinoma. G = Gleason grade; HR = hormone responsive; CRPC = castrate resistant prostate cancer; HN = hormone naïve; ADT = androgen deprivation therapy. TN = Treatment naïve. Bold = studies with unclear concerns for model validity; grey shade = studies with high risk of concern for model validity.
