# Supplementary material for: A systematic review of the validity of patient derived xenograft (PDX) models: the implications for translational research and personalised medicine
Source: PeerJ. 2018 Nov 21;6:e5981. doi: 10.7717/peerj.5981 (PMC6252062; doi:10.7717/peerj.5981)
Supplement: Table S8 [file peerj-06-5981-s008.pdf]

Supplemental Table S8: Outgrowth rates for established PDX lines

A. Breast PDX (primary tumours only. Tissue fragments engrafted unless otherwise indicated)

Study	Mouse strain	Engraftment site	Donor pathology	Donor treatment	Take rate (%)	N stable lines	Definition of outcome
**Fujii 2008**	**NSG**	**subcutaneous**	**100% DC**	**NR**	**5**	**3**	**>P3**
**Shirakawa 2001**	**Balb/c nude, SCID**	**subcutaneous**	**High grade invasive DC (100%)**	**NR**	**10**	**1**	**reached p15**
Zhang 2013	SCID/Bg	orthotopic	IDC (89%)	Previous treatment	13.6	23	NR
Bergamaschi 2009	SCID	subcutaneous	IDC (76%); ILC (14%); MUC (10%)	NR	10	3	P1-P2. A diameter of approximately 15 mm.
Charafe-Jauffret 2013*	NSG	orthotopic	IDC (83.2%); ILC (7%); mixed (1.4%); MED (1.4%); other (7%)	CT (76%); IR (80%); HT (55%)	17.5	13	maintained 13 PDXs through serial passages (>P4)
**Patsialou 2012**	**SCID**	**orthotopic**	**ILC (13%), IDC (87%)**	**NR**	**17**	**5**	**>P1**
**du Manoir 2014**	**Swiss nude**	**subcutaneous**	**NR**	**NR**	**15**	**19**	**P3**
Eyre 2016	NSG	subcutaneous	NR	RT (2%), CT (4%), HT (13%)	9	11	>P2
**McAuliffe 2015**	**Balb/c nude**	**subcutaneous**	**NR**	**Neoadjuvant therapy (50%)**	**27**	**13**	**P3**
Engrafted isolated cells. IDC = invasive ductal carcinoma; ILC = invasive lobular carcinoma; DC = ductal carcinoma; mets = metastases; AC = adenocarcinoma; inv= invasive; MUC = mucinous; MED = medullary. Bold = studies with unclear concerns for model validity; grey shade = studies with high risk of concern for model validity.							

B. Colon PDX (primary tumours only. Tissue fragments engrafted unless otherwise indicated)

Study	Mouse strain	Engraftment site	Donor pathology	Donor treatment	Take rate (%)	N stable lines	Definition of outcome
**Fujii 2008**	**NSG**	**subcutaneous**	**100% AC**	**NR**	**35**	**17**	**>P3**
**Chou 2013***	**NSG**	**subrenal / subcutaneous**	**AC (65%), mAC (23%), AC/NE (5%), NE (5%)**	**CT (18%)**	**25**	**7**	**>P2**
**Guan 2016**	**Balb/c nude**	**subcutaneous**	**AC (89%), mAC (11%)**		**10**	**9**	**reached P6**
**Linnebacher 2010**	**NOD/SCID**	**subcutaneous**	**AC (98%); LNEC (2%)**	**TN**	**23**	**11**	**>P2**
**Julien 2012**	**Swiss nude**	**subcutaneous**	**NR**	**CT (23%)**	**41**	**24**	**late pass**
**Zhou 2011**	**Balb/c nude**	**subcutaneous**	**AC (100%)**	**NR**	**14**	**3**	**Reached P10**
* isolated cells. AC = adenocarcinoma; mAC=mucinous adenocarcinoma; CaG=carcinoma gingiva; Ca= carcinoid; LNEC = large cell neuroendocrine carcinoma; NE = neuroendocrine ; squamous cell carcinoma =SCC, combined small cell carcinoma =SCC; denosquamous =DS, pleomorphic carcinoma = PC. CT = chemotherapy. TN = treatment naïve. Bold = studies with unclear concerns for model validity.							

C. Lung PDX (primary tumours only. Tissue fragments engrafted unless otherwise indicated)

Study	Mouse strain	Engraftment site	Donor pathology	Donor treatment	Take rate (%)	N Stable lines	Definition of outcome
**Leong 2014***	**NSG**	**subcutaneous**	**100% SCLC**	**NR**	**83**	**10**	**Reached P2**
**Fujii 2008**	**NSG**	**subcutaneous**	**100% SQCC**	**NR**	**50**	**1**	**>P3**
**Stewart 2015**	**NOD/SCID**	**subcutaneous**	**100% AC**	**CT, EGFR Tyrosine Kinase inhibitors**	**18**	**6**	**P3**
**Cutz, 2006**	**NOD/SCID**	**subrenal**	**7% squamous metaplasia; 7% carcinoid; 35% SQCC; 14% AC; 21% SCC; 7% carcinosarcoma**	**21% CT, 14% RT**	**77**	**4**	**>P5**
**Hao 2015**	**NOD/SCID**	**subcutaneous**	**48% AC, 39% SQCC, 13% NE**	**NR**	**9**	**8**	**P3**
**Ma 2016**	**NOD/SCID**	**subcutaneous**	**72.1% AC; 21% SQCC; 2.3% LCC; 4.7% SCC**		**37**	**16**	**>P2**
**Zhang 2013**	**SCID, nude**	**subcutaneous**	**52% AC; 3% AC/SCC; 45% SCC**	**Treatment naive**	**32**	**10**	**second generation mice**
Zhuang 2017	NCG	subcutaneous	44% AC; 33% SCC; 22% SQCC	NR	22	2	P2
Fichtner 2008	NOD/SCID, NMRI nude	subcutaneous	48% SQCC; 24% AC; 16% carcinoma with pleimorphic features, 12% LCC/SCC	16% CT or RT	23	25	passagable xenografts
**John 2011**	**NOD/SCID**	**subcutaneous**	**48% AC; 46% SQCC; 5% LCC; 1% sarcomatoid**	**NR**	**40**	**63**	**passaged at least once in vivo**

* isolated cells. TF = tissue fragments. Small cell carcinoma (SCC); non-small cell lung cancer (NSCLC); Adenocarcinoma (AC); Large cell carcinoma (LCC); Adenosquamous carcinoma (ASQC); ductal carcinoma (DC); squamous cell carcinoma (SQCC); neuroendocrine (NE). Chemotherapy (CT); radiotherapy (RT). Bold = studies with unclear concerns for model validity; grey shade = studies with high risk of concern for model validity.

D. Prostate PDX (primary tumours only, all tissue fragments)

Study	Mouse strain	Engraftment site	Donor pathology	Take rate (%)	N Stable lines	Definition of outcome
Lin 2013	NOD/SCID	subrenal	100% AC	31	5	Serial transplantation (excludes lymphomas)
Wang 2005	NOD/SCID	subrenal	100% AC	100	1	up to p5
Russell 2015	nude	subcutaneous	66% AC, 33% AC/SCC	10	3	>p2 (includes fibrosarcoma)
**Pretlow 1993**	**nude**	**subcutaneous**	**50% G9, 25% G7, 25% G8**	**20**	**4**	**P3**
**Klein 1997**	**SCID**	**subcutaneous**	**66% T3, 33% T4**	**33**	**1**	**>P4**
**Yoshikawa 2016**	**SCID**	**subcutaneous, subrenal**	**NR**	**25**	**1**	**Serial transplantation**
van Weerden 1996	NMRI nude	subcutaneous	NR	38.5	5	stable lines

van Weerden 1996	BALB/c nude	subcutaneous	NR	0	0	stable lines
**Fujii 2008**	**NSG**	**subcutaneous**	**NR**	**0**	**0**	**>P3**

AC = Adenocarcinoma; SCC = small cell carcinoma; G = Gleason grade. Bold = studies with unclear concerns for model validity; grey shade = studies with high risk of concern for model validity.
