# Supplementary material for: A systematic review of the validity of patient derived xenograft (PDX) models: the implications for translational research and personalised medicine
Source: PeerJ. 2018 Nov 21;6:e5981. doi: 10.7717/peerj.5981 (PMC6252062; doi:10.7717/peerj.5981)
Supplement: Supplemental Information 1 [file peerj-06-5981-s009.docx]

| 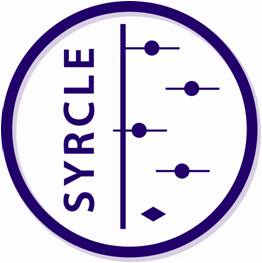 **Systematic Review Protocol for Animal Intervention Studies**  **Format by SYRCLE (**[**www.syrcle.nl**](http://www.syrcle.nl)**)**  **Version 2.0 (December 2014)** | | | | |
| --- | --- | --- | --- | --- |
| **Item #** | **Section/Subsection/Item** | **Description** | | **Check for approval** |
|  | A. General | | | |
| 1. | Title of the review | **A critique of the validation of patient derived xenograft (PDX) models: a systematic review** | |  |
| 2. | Authors (names, affiliations, contributions) | Dr SH Lang (Independent) review design and screening, extraction, synthesis)  Dr A Collins (Dept. Biology, University of York) searching, screening, extraction, synthesis) | |  |
| 3. | Other contributors (names, affiliations, contributions) | None | |  |
| 4. | Contact person + e-mail address | Dr SH Lang, shlang@hotmail.co.uk | |  |
| 5. | Funding sources/sponsors | None | |  |
| 6. | Conflicts of interest | None | |  |
| 7. | Date and location of protocol registration | 17.7.17 York, UK | |  |
| 8. | Registration number (if applicable) |  | |  |
| 9. | Stage of review at time of registration | Scoping searches performed. No other tasks have commenced. | |  |
|  | B. Objectives | | | |
|  | Background | | | |
| 10. | What is already known about this disease/model/intervention? Why is it important to do this review? | Patient derived xenografts are models which allow preclinical testing of human tissue within a mouse. Cells or tissue are derived from a human tumour and inoculated into mice (usually immunocompromised). The tumour outgrowth from these models can be used to test the effects of potential drugs and represent an important preclinical test for personalised medicine. However recent evidence suggests that the models are not always fully validated to ensure the outgrowth within the mouse are a) human b) tumour c) not lymphomas d) derived from the tissue of interest. We aim to review how well validated current published models of patient derived xenografts are. | |  |
|  | Research question | | | |
| 11. | Specify the disease/health problem of interest | Human carcinoma tumours. | |  |
| 12. | Specify the population/species studied | Mouse xenografts derived from human carcinoma tissues (fragments or freshly isolated primary cells ≤3 passages) | |  |
| 13. | Specify the intervention/exposure | NA | |  |
| 14. | Specify the control population | NA | |  |
| 15. | Specify the outcome measures | 1. Engraftment rate (primary outgrowth); percentage of biopsies which develop xenografts. 2. Latency 3. Percentage of biopsies resulting in stable lines 4. Percentage of biopsies resulting in lymphomas. 5. Clinical characteristics of patient tumour tissue   Validation   1. Comparison of xenograft and tumour histology 2. Confirmation of human tumour cells exclusion of mouse cells and normal human cells for primary outgrowth only) 3. Confirmation of tissue type 4. Confirmation of cellular type 5. Confirmation of tumour markers 6. Confirmation of lymphoma or EBV (T cell, B cell, NK cell) | |  |
| 16. | State your research question (based on items 11-15) | How well validated are mouse xenografts derived from human carcinoma tissues | |  |
|  | C. Methods | | | |
|  | Search and study identification | | | |
| 17. | Identify literature databases to search (*e.g.* Pubmed, Embase, Web of science) | XMEDLINE via PubMed □Web of Science  □SCOPUS XEMBASE  □Other, namely:  □Specific journal(s), namely: | |  |
| 18. | Define electronic search strategies (*e.g.* use the [step by step search guide^15^](http://www.ncbi.nlm.nih.gov/pmc/articles/PMC3265183/pdf/LA-11-087.pdf) and animal search filters[^20,^](http://www.ncbi.nlm.nih.gov/pmc/articles/PMC3104815/pdf/LA-09-117.pdf) [^21^](http://lan.sagepub.com/content/48/1/88.full.pdf+html)) | When available, please add a supplementary file containing your search strategy: [insert file name] | |  |
| 19. | Identify other sources for study identification | XReference lists of included studies □Books  □Reference lists of relevant reviews  □Conference proceedings, namely:  □Contacting authors/ organisations, namely:  □Other, namely: | |  |
| 20. | Define search strategy for these other sources | NA | |  |
|  | Study selection | | | |
| 21. | Define screening phases (*e.g.* pre-screening based on title/abstract, full text screening, both) | Search results will be loaded onto the systematic review web app, Rayyan, to allow title and abstract screening. Titles and abstracts identified through electronic database and web searching will be independently screened by two reviewers. Title and abstract screening will be based on population, language and publication type only.  Articles meeting the inclusion criteria will be obtained as full paper copies and examined independently by two reviewers to determine whether the full paper meets the inclusion criteria of the review. All papers excluded at this second stage of the screening process will be documented along with the reasons for exclusion. | |  |
| 22. | Specify (a) the number of reviewers per screening phase and (b) how discrepancies will be resolved | Two independent reviewers will be used at all stages of the review process. Discrepancies will be resolved by consensus. | |  |
|  | *Define all inclusion and exclusion criteria based on:* | | | |
| 23. | Type of study (design) | Inclusion criteria: Original research papers  Exclusion criteria: Reviews, conference abstracts, commentaries. | |  |
| 24. | Type of animals/population (*e.g.* age, gender, disease model) | Inclusion criteria: Mouse xenografts derived from human carcinoma tissues (fragments or freshly isolated primary cells ≤3 passages)  Exclusion criteria: Metastatic tumours.  Lymphoma, leukaemia, sarcomas, melanoma, brain tumours (glioma, medullablastoma etc.), squamous carcinoma, cystic carcinoma, teratoma. Established xenograft models. Xenografts derived from cell lines or primary samples ≥4 passages. Rat, canine xenografts. | |  |
| 25. | Type of intervention (*e.g.* dosage, timing, frequency) | Inclusion criteria: NA  Exclusion criteria: NA | |  |
| 26. | Outcome measures | Inclusion criteria: Percentage of biopsies which develop lymphomas; Latency; Percentage of biopsies resulting in stable lines; Percentage of biopsies resulting in lymphomas; Clinical characteristics of patient tumour tissue; Xenograft and tumour histology; Confirmation of human tumour cells exclusion of mouse cells and normal human cells for primary outgrowth only); Confirmation of tissue type; Confirmation of cellular type; Confirmation of tumour markers; Confirmation of lymphoma or EBV (T cell, B cell, NK cell)  Exclusion criteria: NA | |  |
| 27. | Language restrictions | Inclusion criteria: English language  Exclusion criteria: NA | |  |
| 28. | Publication date restrictions | Inclusion criteria: None  Exclusion criteria: None | |  |
| 29. | Other | Inclusion criteria:  Exclusion criteria: Purchased models or models validated in other publications (original will be sought for inclusion).  If the review is too large we will restrict the tissue types. Order of importance will be: prostate, breast, colon, pancreas, lung, ovary, bladder, gastric, all others.  We will aim to include all publications, but those not validating the models will only have study details extracted. | |  |
| 30. | Sort and prioritize your exclusion criteria per selection phase | All exclusion criteria were applied at the inclusion screening process but not during searches. | |  |
|  | Study characteristics to be extracted (for assessment of external validity, reporting quality) | | | |
| 31. | Study ID (*e.g.* authors, year) | First author and publication year | |  |
| 32. | Study design characteristics (*e.g.* experimental groups, number of animals) | Clinical characteristics of patient tumour tissue;  number of animals, number of cells inoculated, size of tissue inoculated, other factors inoculated | |  |
| 33. | Animal model characteristics (*e.g.* species, gender, disease induction) | Type of mouse (eg NOD SCID) | |  |
| 34. | Intervention characteristics (*e.g.* intervention, timing, duration) | NA | |  |
| 35. | Outcome measures | Confirmation of human tumour cells (exclusion of mouse cells and normal human cells for primary outgrowth only); Confirmation of tissue type; Confirmation of cellular type; Confirmation of tumour markers; Confirmation of lymphoma or EBV (T cell, B cell, NK cell) | |  |
| 36. | Other (*e.g.* drop-outs) | Percentage of biopsies which develop lymphomas; Latency; Percentage of biopsies resulting in stable lines; Percentage of biopsies resulting in lymphomas; Xenograft and tumour histology; | |  |
|  | Assessment risk of bias (internal validity) or study quality | | | |
| 37. | Specify (a) the number of reviewers assessing the risk of bias/study quality in each study and (b) how discrepancies will be resolved | Two independent reviewers will perform risk of bias. Discrepancies will be resolved by consensus. | |  |
| 38. | Define criteria to assess (a) the internal validity of included studies (*e.g.* selection, performance, detection and attrition bias) and/or (b) other study quality measures (*e.g.* reporting quality, power) | XBy use of [SYRCLE's Risk of Bias tool^4^](http://www.biomedcentral.com/1471-2288/14/43/abstract)  □By use of SYRCLE’s Risk of Bias tool, adapted as follows:  □By use of [CAMARADES' study quality checklist, e.g ^22^](http://www.ncbi.nlm.nih.gov/pubmed/15060322)  □By use of CAMARADES' study quality checklist, adapted as follows:  XOther criteria, namely: model validity and imprecision using the tools published in Collins et al 2017 https://doi.org/10.1371/journal.pone.0178645 | |  |
|  | Collection of outcome data | | | |
| 39. | For each outcome measure, define the type of data to be extracted (*e.g.* continuous/dichotomous, unit of measurement) | Confirmation analyses will be yes/no for each study; accompanied by description of methods if there is a yes.  Percentage of biopsies = dichotomous data  Latency = continuous data in days or months | |  |
| 40. | Methods for data extraction/retrieval (*e.g.* first extraction from graphs using a digital screen ruler, then contacting authors) | Data will be taken from graphs, but not if measurements are required. Authors will not be contacted for additional data or clarifications. | |  |
| 41. | Specify (a) the number of reviewers extracting data and (b) how discrepancies will be resolved | Two independent reviewers will perform extractions. Discrepancies will be resolved by consensus. | |  |
|  | Data analysis/synthesis | | | |
| 42. | Specify (per outcome measure) how you are planning to combine/compare the data (*e.g.* descriptive summary, meta-analysis) | Narrative summary for all confirmation analyses.  Percentage of biopsies will be narrative or meta-analysis (dependent on number of studies for outcome). Subgroup analysis will be performed for tumour grade and type of mouse.  Latency will be narrative or meta-analysis (dependent on number of studies for outcome). Subgroup analysis will be performed for tumour grade and type of mouse. | |  |
| 43. | Specify (per outcome measure) how it will be decided whether a meta-analysis will be performed | Meta-analysis will only be performed if more than 3 studies provide data for the outcome. | |  |
|  | *If a meta-analysis seems feasible/sensible, specify (for each outcome measure):* | | | |
| 44. | The effect measure to be used (*e.g.* mean difference, standardized mean difference, risk ratio, odds ratio) | Percentage of biopsies will be risk ratio.  Latency will be mean difference. | | All effect measures if presented. |
| 45. | The statistical model of analysis (*e.g.* random or fixed effects model) | Both random and fixed effects will be considered | | All statistical methods will be extracted and reported |
| 46. | The statistical methods to assess heterogeneity (*e.g.* I^2^, Q) | I^2^ | | NA |
| 47. | Which study characteristics will be examined as potential source of heterogeneity (subgroup analysis) | Subgroup analysis will be performed for tissue type, tumour grade and type of mouse. | | NA |
| 48. | Any sensitivity analyses you propose to perform | No | | NA |
| 49. | Other details meta-analysis (*e.g.* correction for multiple testing, correction for multiple use of control group) | NA | | NA |
| 50. | The method for assessment of publication bias | Eggers (if more than 10 studies only) | | NA |
|  | | | | |
| Final approval by (names, affiliations): | |  | Date: | |
