# Supplementary material for: A systematic review of the validity of patient derived xenograft (PDX) models: the implications for translational research and personalised medicine
Source: PeerJ. 2018 Nov 21;6:e5981. doi: 10.7717/peerj.5981 (PMC6252062; doi:10.7717/peerj.5981)
Supplement: Supplemental Information 2 [file peerj-06-5981-s010.docx]

Systematic Review/Meta Analysis

1. The rationale for conducting the Systematic review/meta analysis:

We sought to objectively assess the validity and reliability of PDX models as a platform for preclinical research in the four most common cancers: breast, prostate, colon and lung.

1. The contribution that the systematic review/meta analysis makes to knowledge in light of previously published related reports:

This is the first systematic review of PDX models to provide a comprehensive assessment of their validity by developing a structured tool (Collins & Lang, 2017) to assess quality based on empirical evidence. We identified three other systematic reviews of PDX models; one followed PRISMA guidance (Brown et al.,2016) and two did not follow PRISMA (Jin et al., 2010; Lopez-Barcons, 2010). None of the systematic reviews formally presented any quality assessment of the primary studies. Our review represents a major step forward following approved systematic review reporting guidelines (PRISMA); presenting a formal assessment (rather than subjective) of the validity/quality of the included studies. This work provides high quality evidence of the validity of PDX models, which are frequently used for translational studies and personalised medicine. This evidence aids decision making in the selection of the best cancer models and identifies evidence gaps for future research.

Searches were performed by the authors (SHL and ATC). Manuscript p5, line 17

Any discrepancies between reviewers were resolved through consensus. Manuscript p6, lines 20-21.

References

1. Collins, A., Ross, J. & Lang, S. H. A systematic review of the asymmetric inheritance of cellular organelles in eukaryotes: A critique of basic science validity and imprecision. *PLOS ONE* **12**, e0178645 (2017).
2. Brown, K. M. *et al.* Patient-derived xenograft models of colorectal cancer in pre-clinical research: a systematic review. *Oncotarget* **7,** 66212-66225 (2016).
3. Jin, K. *et al.* Patient-derived human tumour tissue xenografts in immunodeficient mice: A systematic review. *Clinical and Translational Oncology* **12,** 473–480 (2010).
4. Lopez-Barcons, L. A. Human prostate cancer heterotransplants: A review on this experimental model. *Asian Journal of Andrology* **12,** 509–518 (2010).
