# Supplementary material for: A systematic review of the validity of patient derived xenograft (PDX) models: the implications for translational research and personalised medicine
Source: PeerJ. 2018 Nov 21;6:e5981. doi: 10.7717/peerj.5981 (PMC6252062; doi:10.7717/peerj.5981)
Supplement: Figure S1 — Each study was assessed to determine if PDX models were at a high risk of concern for each signalling question related to reporting. The graph indicates the percentage of studies that provided a: clear and full description (white bars), no reported details (black bars), partial description (grey bars). Breast (n = 29 studies). Colon (n = 31 studies). Lung (n = 25 studies). Prostate (n = 20 studies). [file peerj-06-5981-s012.pdf]

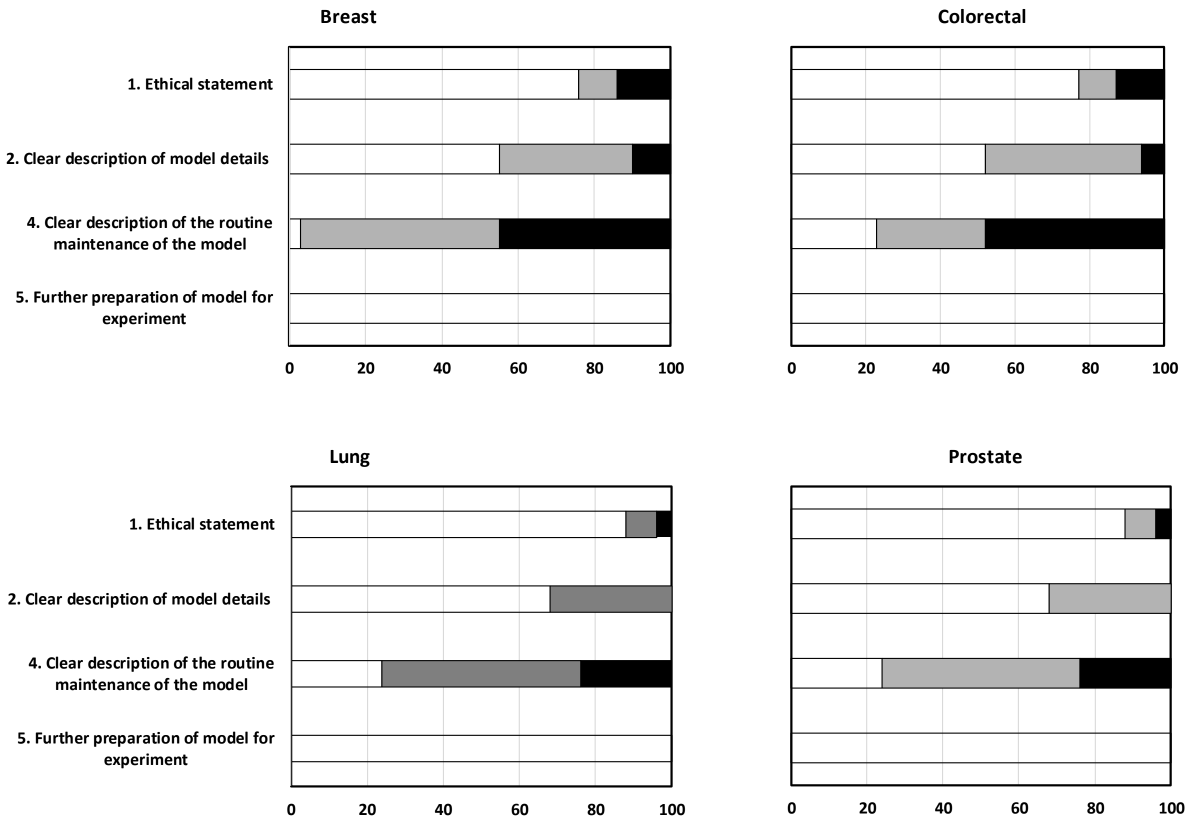

Supplemental Figure S1. **Model validity (reporting questions).** Each study was assessed to determine if PDX models were at a high risk of concern for each signalling question related to reporting. The graph indicates the percentage of studies that provided a: clear and full description (white bars), no reported details (black bars), partial description (grey bars). Breast (n=29 studies). Colon (n=31 studies). Lung (n=25 studies). Prostate (n= 20 studies).
