# Supplementary material for: A systematic review of the validity of patient derived xenograft (PDX) models: the implications for translational research and personalised medicine
Source: PeerJ. 2018 Nov 21;6:e5981. doi: 10.7717/peerj.5981 (PMC6252062; doi:10.7717/peerj.5981)
Supplement: Figure S2 — Each study (n = 105) was assessed to determine if authors performed any experiments to answer a given question 8 criterion. a. was tissue of origin proven? b. confirmation that the PDX was derived from a given patient, c. was the cell lineage proven? d. confirmation that the PDX was derived from tumour and not normal cells, e. absence of murine overgrowth, f. was there comparable histopathology? g. concordance for standard of care agents? h. was the absence of lymphoma proven? The graph indicates the percentage of studies that: performed experiments to clearly answer the question (white bars), did not perform any experiments to answer the question (black bars), provided partial results to answer the question (grey). [file peerj-06-5981-s013.pdf]

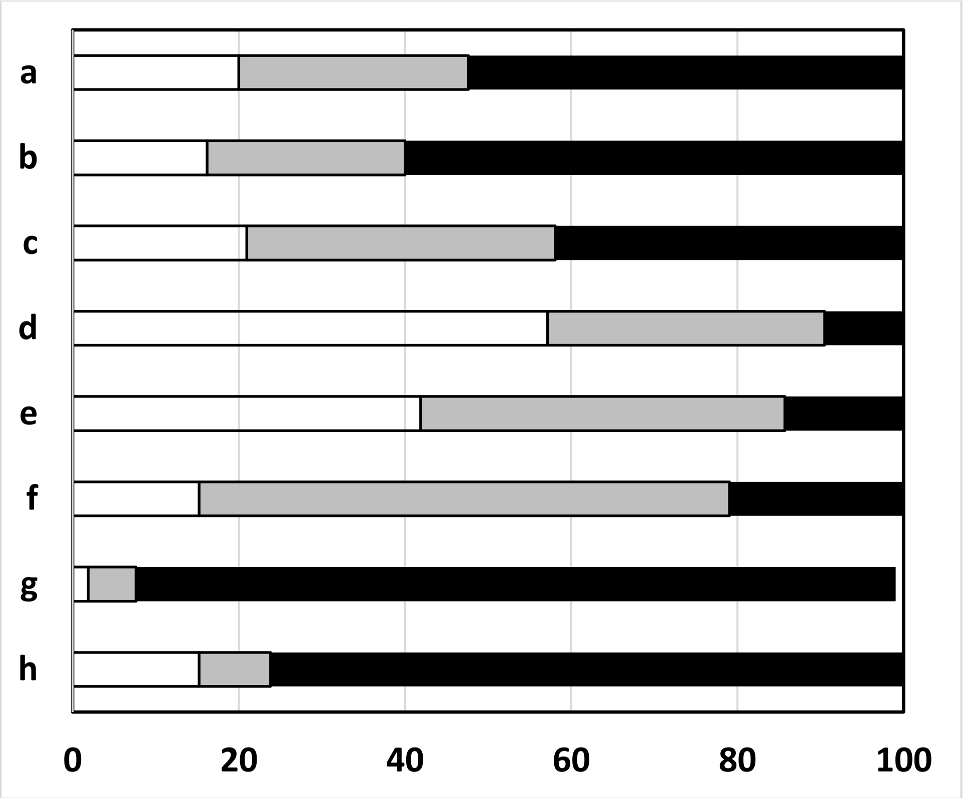

Supplemental Figure S2. Assessment of studies that carried out experiments to answer validity questions 8. Each study (n=105) was assessed to determine if authors performed any experiments to answer a given question 8 criterion. a. was tissue of origin proven? b. confirmation that the PDX was derived from a given patient, c. was the cell lineage proven? d. confirmation that the PDX was derived from tumour and not normal cells, e. absence of murine overgrowth, f. was there comparable histopathology? g. concordance for standard of care agents? h. was the absence of lymphoma proven? The graph indicates the percentage of studies that: performed experiments to clearly answer the question (white bars), did not perform any experiments to answer the question (black bars), provided partial results to answer the question (grey).
